# Assessment of the Presence of Transformation Products of Certain Pharmaceutical Products (Psychotropic Family) by Suspect and Non-Targeted HRMS Screening in Wastewater Treatment Plants

**DOI:** 10.3390/toxics11080713

**Published:** 2023-08-18

**Authors:** Solenne Reverbel, Marie-Hélène Dévier, Valentin Dupraz, Emmanuel Geneste, Hélène Budzinski

**Affiliations:** 1 CNRS, Bordeaux INP, University of Bordeaux, EPOC, UMR 5805, LPTC, F-33600 Pessac, France; 2 Régie de l’Eau Bordeaux Métropole, Direction de la Recherche, de l’Innovation et de la Transition Ecologique, F-33081 Bordeaux, France

**Keywords:** high resolution mass spectrometry, psychotropic drugs, transformation products, wastewater treatment plants

## Abstract

Aquatic environments are the final receptors of human emissions and are therefore contaminated by molecules, such as pharmaceuticals. After use, these compounds and their metabolites are discharged to wastewater treatment plants (WWTPs). During wastewater treatment, compounds may be eliminated or degraded into transformation products (TPs) or may be persistent. The aim of this study was to develop an analytical method based on high resolution mass spectrometry (HRMS) for the identification of six psychotropic drugs that are widely consumed in France and present in WWTPs, as well as their potential associated metabolites and TPs. Four out of six psychotropic drugs and between twenty-five and thirty-seven potential TPs were detected in wastewater, although this was based on full scan data. TPs not reported in the literature and specific to the study sites and therefore to the wastewater treatment processes were tentatively identified. For the selected drugs, most known and present TPs were identified, such as desmethylvenlafaxine or norcitalopram. Moreover, the short fragmentation study led rather to the identification of several TPs of carbamazepine as ubiquitous persistent TPs.

## 1. Introduction

A wide range of pharmaceutical compounds are currently used around the world and are released into the aquatic environment [1,2,3]. These pharmaceuticals, such as antibiotics, analgesics, hormones, lipid regulators, beta blockers, and psychotropic drugs, are found in surface waters at concentrations ranging from ng L^−1^ to µg L^−1^ [1,4,5,6,7,8,9,10]. The discharge of these compounds into the aquatic environment can be attributed to aerial sources, agriculture, industry, and hospital and domestic discharges via WWTPs [1,11]. WWTPs have been identified as the primary source of pharmaceutical compound emissions to water bodies [2,12,13,14]. After consumption, drugs may or may not be metabolized [2,3,13]. Then, urine and feces containing parent compounds and formed metabolites are transported to the WWTPs. Thus, numerous drugs are found not only in influent but also in effluent at concentrations of up to µg L^−1^ for certain pharmaceutical compounds [15,16,17,18]. Metabolites from drugs are also detected both in influent and effluent from WWTPs [19,20,21,22].

Psychotropic drugs are among the drugs and metabolites found in wastewater. In France, the consumption of psychotropic drugs is relatively high. In 2021, three anxiolytics and three antidepressants appeared in very high ranks according to the ranking of the number of boxes of drugs (1210 listed medicines) purchased in France (and therefore, presumably, consumed); alprazolam, oxazepam, venlafaxine, fluoxetine, diazepam and citalopram came in 12th, 18th, 40th, 55th, 145th, and 267th place, respectively [23]. These six highly consumed drugs are largely discharged into the WWTP. They have been detected in the influent and the effluent of WWTPs at concentrations within the ng L^−1^ range, sometimes up to µg L^−1^ [24,25,26,27,28]. For instance, venlafaxine was detected between 23 and 567 ng L^−1^ in the influent and between 2 and 429 ng L^−1^ in the effluent [28]. The metabolites associated with these six psychotropic drugs have also been detected in raw and treated wastewaters during targeted analysis [6,24,26,29,30]. O-desmethyl venlafaxine, a metabolite of venlafaxine, was reported at a mean of 420 ng L^−1^ in the influent and 109 ng L^−1^ in the effluent during the autumn [26]. Furthermore, desmethylcitalopram, a metabolite of citalopram, has been detected in WWTP influent at concentrations between 55 and 133 ng L^−1^ and in WWTP effluent at concentrations between 36 and 310 ng L^−1^ [31].

Pharmaceutical compounds, such as the psychotropic drugs mentioned above and their metabolites that are present in the effluents and therefore not totally eliminated during the water treatment processes, are then discharged into the natural water bodies. Wastewater treatment processes such as primary treatments, secondary treatments (conventional activated sludge (CAS), membrane bioreactors (MBR), sequencing batch reactors (SBRs), and constructed wetlands) and tertiary treatments (UV disinfection, ozonation, and activated carbon) can more or less efficiently remove some pharmaceutical compounds. The elimination of molecules in WWTPs is achieved by several main reactions such as biotransformation, sorption, biodegradation [13,32,33] or abiotic oxidation taking place during tertiary treatments. Photodegradation, hydrolysis and volatilization reactions, although less important than those mentioned above, may also occur [32,33]. The elimination rates of WWTP are very variable. It depends on several parameters such as operating conditions (water temperature, pH, hydraulic retention time, and solid retention time) and the type of treatment [33,34,35,36]. For instance, several studies have shown that CAS—a secondary biological treatment—has the lowest removal rates for pharmaceutical compounds [34,37,38,39]. Currently, based on several studies, it appears that the combination of biological treatment such as MBR followed by treatment with advanced oxidation processes or activated carbon would be most effective in removing pharmaceutical compounds [33,40,41,42]. In addition, the removal rate of pharmaceutical compounds also depends on their physicochemical properties (polarity, water solubility, and log K_ow_, K_d_) [13,33]. For instance, in the case of psychotropic drugs, they are more or less refractory to wastewater treatment. Citalopram and alprazolam are eliminated by about 10%, oxazepam by about 13%, fluoxetine by about 33%, venlafaxine by about 40%, and diazepam by about 45% in a WWTP using CAS [32,43,44].

However, the degradation of pharmaceuticals such as psychotropic drugs during wastewater treatment does not necessarily mean their complete elimination. In fact, some are not completely degraded and are transformed into new molecules called TPs during the wastewater treatment process [45,46,47,48]. TPs are produced from various reactions that the parent compound may undergo in the WWTP (i.e., biological transformation, chemical transformation, or disinfection byproducts) [33,49,50]. Venlafaxine, for example, is a compound that can be transformed into TPs not only by biotransformation [45] but also by chemical pathways [51,52]. Furthermore, in the context of a WWTP that applies CAS treatment, ozonation treatment, and post-treatment, for example, a compound may undergo 10 different behaviors and thus produce different types of TPs during the different wastewater treatment processes [47]. Occasionally, these TPs may be more bioaccumulative, toxic, and persistent than the compound from which they are derived [20,33,36,49,53,54,55].

Moreover, TPs generated during wastewater treatment are therefore added to the compounds and metabolites already present in the water as they reach the WWTP. In fact, after the ingestion of psychotropic drugs, part of the ingested dose of active compounds is metabolized by the human organism before being excreted in urine and feces to the WWTP. For instance, 19% of the citalopram dose is excreted as desmethylcitalopram (i.e., norcitalopram) [30]. These metabolites, already present at the WWTP inlet, can also be degraded into TPs during wastewater treatment and thus be added to the TPs discharged into the aquatic environment via the WWTP effluents.

Currently, the vast majority of TPs remain unknown. Their identification can be performed using analytical techniques based on HRMS [56,57,58,59,60,61]. HRMS provides the ability to analyze a large number of both known and unknown compounds such as TPs/metabolites in complex matrices [56,62]. This technique is mostly based on hybrid instruments such as quadrupole time-of-flight (Q-TOF) or linear ion trap/orbitrap (LTQ Orbitrap). Thus, the study of TPs is possible with these instruments, allowing us to detect compounds in various matrices with high mass accuracy and resolution [56]. There are three methodologies of analysis in HRMS: targeted analysis, suspect screening, and non-target screening [56]. Targeted analysis is a quantitative and very specific method used to search for a limited list of compounds using reference standards. Suspect screening is a technique designed to find a list of “suspect” compounds in samples. After acquisition and data processing, a list of likely present suspects is generated. In this study, we have chosen to use this type of analysis because it is the one that best meets our objectives. Indeed, it is relevant and is used to not only search for a large number of compounds but also to detect and attempt to identify new relevant compounds in aqueous matrices [58,63]. Non-targeted analysis is a non-specific and qualitative method. All the molecular masses present in the samples are detected and these features can be classified with different confidence levels [59]. There are five levels ranging from 1 to 5, level 1 being the highest. To reach level 1 (confirmed structure), the data obtained for the proposed structure (MS, MS^2^, RT) must be similar to those obtained after injection of the analytical standard. Level 2 is applied to compounds for which a probable structure has been determined by library spectrum match or by diagnostic evidence. Then, level 3 is applied to molecules for which several possible structures have been determined; level 4 is used for compounds for which a formula could be assigned; finally, level 5 is applied to molecules for which only the *m*/*z* could be obtained.

The aim of this study was, therefore, (i) to develop a methodology to monitor alprazolam, citalopram, diazepam, fluoxetine, oxazepam, venlafaxine, and their potential associated TPs in raw and treated water using a suspect screening method based on liquid chromatography coupled with high resolution mass spectrometry (LC-QTOF), (ii) to test the applicability of this method to complex matrices (WWTPs), and (iii) to tentatively identify potential TPs and finally classify the behavior of the TPs according to different wastewater treatment processes. This classification allowed us to select TPs according to their relevance. Indeed, TPs that were both not eliminated or potentially generated by wastewater treatment were considered relevant because they might be discharged to surface water via WWTP effluents. Thus, these selected potentials TPs were searched and analyzed in MS and MS/MS mode in order to increase the confident level in the identification and to confirm their presence.

## 2. Materials and Methods

### 2.1. Chemicals and Materials

Methanol (MeOH) and acetonitrile (ACN) (HPLC grade quality) were purchased from J.T Baker (Deventer, The Netherlands), dichloromethane (DCM) from Acros Organics (Geel, Belgium), and formic acid (FA) (98–100%) from Merck (Darmstadt, Germany). Milli-Q grade water was produced with a Milli-Q system equipped with an EDS Pack (Millipore SA, Saint-Quentin-les-Yvelines, France). Pure analytical standards (alprazolam, citalopram, diazepam, fluoxetine, oxazepam, and venlafaxine) used for standard solutions and quality controls were of all analytical grade (purity > 95%) and supplied by Sigma-Aldrich (Saint Quentin Fallavier, France), AlsaChim (Strasbourg, France), and Toronto Research Chemicals (Toronto, ON, Canada). Stock solutions were gravimetrically prepared in acetonitrile or methanol and stored at −20 °C. Working solutions were prepared by dilution of individual stock solutions in methanol or acetonitrile and stored at −20 °C for no more than six months.

### 2.2. Sampling

Three wastewater treatment plants (called WWTP1, WWTP2, and WWTP3) with different capacities and treatment processes located in two cities in Gironde (southwest France) were selected in this study. Information on capacities, treatment processes, and flow rates of the selected WWTPs is summarized in Table 1. Twenty-four hour averaged samples were collected in the 3 WWTPs with an automatic sampler. Raw and treated waters from WWTP3 were sampled during a first campaign in 2018 and those from WWTP1 and WWTP2 during a second campaign in winter 2020. The wastewater samples from each site were filtered on glass filtration units through a 1.6 µm glass fiber filter (Whatman^®^, Buckinghamshire, UK) and then through a 0.7 µm glass fiber filter (Whatman^®^). They were stored at −20 °C in the dark until extraction and analysis.

### 2.3. Analytical Procedure

#### 2.3.1. SPE Extraction

A solid-phase extraction (SPE) was performed on the wastewater samples using Oasis^®^ HLB cartridges (200 mg, 6 cc) (Waters, Mildford, MA, USA). Cartridges were first conditioned with 5 mL DCM, then with 5 mL MeOH, and finally with 2 × 5 mL Milli-Q water. Wastewater samples and a blank sample (Milli-Q water) were passed through the cartridges by vacuum suction. The cartridges were then dried under vacuum for 1h. Finally, the analytes were eluted first with 2 × 5 mL MeOH, then with 2 × 5 mL of a MeOH/DCM mixture (50:50, *v*/*v*), and last with 2 × 5 mL DCM. Extracts were evaporated nearly to dryness using a Rapidvap^®^ (Labconco, Missouri, USA) at 40 °C. One mL samples were then reconstituted in vials by adding MeOH. The samples were stored at −20 °C until analysis.

#### 2.3.2. LC-QTOF Analysis

The determination of drugs and TPs in the WWTP samples were performed on an Agilent Infinity 1290 HPLC system coupled to an Agilent 6540 accurate mass quadrupole time-of-flight mass spectrometer (LC-QTOF Agilent Technologies, Santa Clara, CA, USA) operated in the positive ionization mode (ESI+). Analytes (3 µL sample) were separated using a Kinetex C18 column (100 mm × 2.1 mm, 1.7µm; Phenomenex^®^, Agilent Technologies, Les Ulis, France) at 0.3 mL min^−1^ and 40 °C with Milli-Q water and acetonitrile, both acidified with 0.1% FA, as aqueous (A) and organic (B) solvents, respectively. Gradient LC elution was performed as follows: from 90% A at 0 min, to 60% A at 22 min, then to 0% A at 30 min, held for 2 min, back to 90% A at 34 min, and held until 38 min. The QTOF instrument was operated in full scan MS mode (Extended Dynamic Range 2 GHz) over the range 50–1700 *m*/*z* from an acquisition rate of 1 spectrum s^−1^. ESI source and MS parameters were: 300 °C drying gas temperature, 8 L min^−1^ gas flow, 40 psig nebulizer, 400 °C sheath gas temperature, and 11 L min^−1^ sheath gas flow. The capillary voltage was 3 kV, nozzle voltage 0.5 kV, fragmentor voltage 110 V, skimmer 65 V, and octopole 1 RF voltage 750 V.

The QTOF instrument was also operated in data-dependent MS/MS (Auto MS/MS) using the same chromatographic and MS source parameters as above, with MS and MS/MS *m*/*z* ranges of 50–1000 and 50–640, respectively. Precursors (list of selected suspect TPs) were selected using a narrow isolation width (*ca.* 1.3 *m*/*z*), a mass accuracy less than 5 ppm, and a retention time window of ±0.5 min and fragmented at three collision energies: 10, 20, and 40 eV.

An Agilent TOF reference solution continuously flowing through the reference nebulizer allowed to correct any mass drift by using the reference mass ion HP-921 (hexakis-(1H,1H,3H-tetrafluoropropoxy)-phosphazine) at *m*/*z* 922.0098 [M + H]^+^. The instrument resolution for ESI+ was 11,100–30,000 at *m*/*z* 118.0863 and 1521.9715, with mass accuracy below 5 ppm. Prior to performing non-target chemical analyses on WWTP sample extracts, routine quality controls were performed to ensure the adequate performance of the instrument. Briefly, a control card solution containing a mixture of nine (ESI+) or five (ESI-) pharmaceutical standards were injected (about 20 pg) in ESI+ to ensure that the following criteria were met for each compound: mass accuracy threshold of 2 mDa and 5 ppm and less than 0.3 min drift in retention time, similar peak areas, signal to noise ratio (S/N), and detection limits compared with previously validated quality controls.

Raw and treated samples were injected in triplicate for WWTP3 samples and only once for WWTP1 and WWTP2 samples. The acquired data were processed with Agilent MassHunter Qualitative Analysis (version B.07.00 and version B.10.00).

#### 2.3.3. Data Treatment

**Figure 1:**
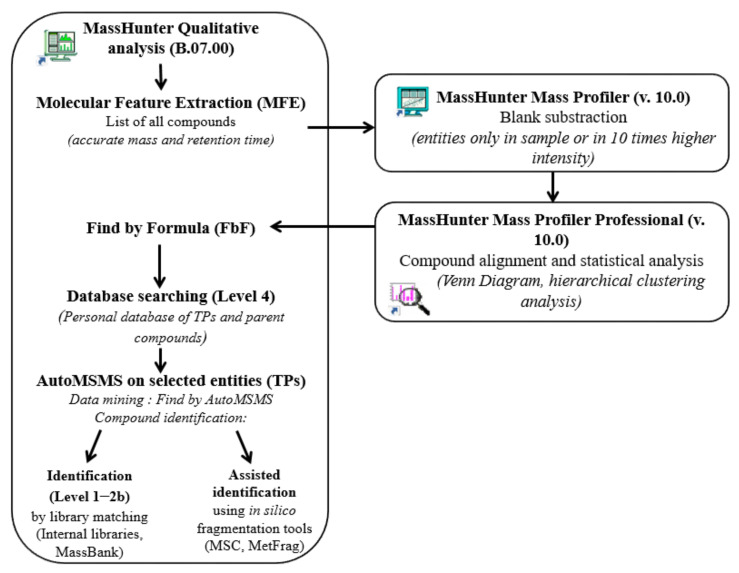
Data processing workflow.

A search strategy based on the data processing method was developed, using tools from MassHunter Qualitative Analysis software and Mass Profiler (MP) and Mass Profiler Professional (MPP) software (Figure 1). It was designed to identify psychotropic drugs as well as TPs present in raw and treated waters. First, chemical entities were extracted for each sample using the “Molecular Feature Extraction (MFE)” algorithm. Filters were: ion species: [M + H]^+^, [M + Na]^+^, [M + NH_4_]^+^, peak height > 1000 counts; compound height > 30,000 counts; quality score ≥ 80. Compound filters were relatively high in order to reduce the number of extracted compounds from these complex matrices (and prevent too many false positives). After this step, a blank subtraction was performed using Mass Profiler software (v. 10.0). Only the features present in the sample and those present with an intensity 10 times higher than the same detected in the procedural blank sample (Milli-Q water) were retained for further data processing. Then, chemical entities were imported into the Agilent chemometric package Agilent Mass Profiler Pro (MPP) (v. 13.1.1) for compound alignment and statistical prioritization (PCA, Venn diagram, and hierarchical clustering analysis). Using these data, the behavior of the entities in the WWTPs was studied (elimination, formation, and persistence). Thus, potential relevant entities were observed and were then selected on the heat maps (hierarchical clustering) for further investigation. For each sample, chemical entities that were not present in the three replicates (for the WWTP3 sample) were filtered out. Then, the MPP files were re-imported in MassHunter Qualitative Analysis and the features detected in the wastewaters were identified using the “Find by Formula” algorithm (FBF). The database used was an Excel file containing all the suspected parent and transformation compounds (see Section 3.1). The overall match score (score > 75%) of the features retained included fit with the exact mass (error < 5 ppm) and the isotopic profile (isotope abundance and isotope spacing) of the compound. They were then considered as “potential” parent compounds and TPs. After this step, analytical standards of alprazolam, citalopram, diazepam, fluoxetine, oxazepam, and venlafaxine were used to confirm the identification of parent compounds (level 1). Moreover, MS/MS analysis was performed to obtain fragmentation spectra of some relevant TPs. Chemical entities were tentatively identified by comparison of experimental MS/MS spectra with those of our internal spectral library, as well as those provided by the Agilent software (personal compound databases and libraries, PCDLs), namely ForensicsTox, Metlin and Metlin_Metabolites, Pesticides, and Water. Additionally, the Agilent molecular structure correlator (MSC), the MetFrag tool, and the MassBank database were also used to increase the identification confidence. The further identification process using these in silico tools is still under finalization; however, some examples of identification by MS/MS experiments are given as proof of concept.

## 3. Results and Discussion

### 3.1. Lists for Suspect Screening

In order to develop an analytical method for suspect screening using HRMS, two lists of suspect compounds were established. The first list contained the six parent psychotropic drugs: alprazolam, citalopram, diazepam, fluoxetine, oxazepam, and venlafaxine. They were chosen following various parameters such as high consumption, occurrence in WWTPs, and ability to generate TPs. Their names, as well as their formula and mass, are represented in Table 2. A second list of suspects, including the 6 psychotropic drugs and 251 of their tentative metabolites/TPs, was then compiled using the scientific literature and an Excel macro that allowed us to predict the various biotic and abiotic reactions that contaminants can undergo in water. Thus, among the TPs searched, a great part was generated by computer and has never been studied before. This Excel spreadsheet has been developed from the non-commercial and publicly available online in silico prediction tool EAWAG-Biocatalysis/Biodegradation Database Pathways Prediction System (EAWAG-BBD/PPS) (http://eawag-bbd.ethz.ch/), accessed on 1 April 2022, under the supervision of the Swiss Federal Institute of Aquatic Science and Technology (EAWAG), redesigned, reimplemented, upgraded, and renamed enviPath (https://envipath.org/) accessed on 1 April 2022. This tool allows the prediction of different biological transformation pathways of contaminants [64,65,66,67,68,69]. All the information collected on the psychotropic drugs and TPs (name, mass, elemental composition, and origin) was gathered in an Excel spreadsheet. The TPs from the literature review have the same nomenclature as in the paper they were studied in. The TPs generated by the Excel spreadsheet have been given specific nomenclatures: TPs from alprazolam are called TP-ALP-X; those of citalopram, TP-CTR-X; those of diazepam, TP-DIA-X; those of fluoxetine, TP-FLX-X; those of oxazepam, TP-OXA-X; those of venlafaxine, TP-VFX-X. Then, a database was created from this Excel spreadsheet in the Agilent PDCL software (databases and libraries, version B.08.00). The total number of TPs compiled from the literature and via the in silico prediction tool for each compound is summarized in Table 2. The name, formula, mass, and origin of the 251 TPs [45,51,70,71,72,73,74,75,76,77,78,79,80] are represented in the Supplementary Information (SI), Tables S1–S6.

### 3.2. Occurrence and Identification of Compounds in WWTPs Samples

The proposed analytical strategy was used to search for and tentatively identify psychotropic drugs and their TPs. Despite the numerous advantages of this strategy (e.g., semi-automatic method and identification of unknowns), some drawbacks were also noted. Indeed, a high number of features, up to several thousands, were detected in this complex matrix after screening by MFE in spite of the use of filters. Therefore, the use of filters seems to be necessary to reduce the number of extracted features but also to decrease the number of false positives [70]. In addition, after the screening by FBF, the obtained compound chromatograms and MS spectra were manually checked with the same objective. Moreover, the presence (or absence) of the six parent drugs was confirmed by the injection of analytical standards. AutoMS/MS analyses using a preferred list of relevant TPs, that is, TPs not eliminated during wastewater treatment and those potentially generated during wastewater treatment, were also performed to confirm and increase the confidence level in the identification of the potential TPs. For fragmented compounds for which no fragmentation spectra were available in libraries, tentative assisted-identification using in silico fragmentation tools (Metfrag and Agilent MSC) was performed and is still under finalization.

#### 3.2.1. Occurrence of the Six Parent Psychotropic Drugs

Of the six investigated psychotropic drugs, between three and four were detected across sites and water types. In the raw and treated waters of WWTP2 and WWTP3, three compounds were found: citalopram, oxazepam, and venlafaxine. For WWTP1, four compounds (citalopram, fluoxetine, oxazepam, and venlafaxine) were detected in the raw water while three compounds were found in the treated water (citalopram, oxazepam, and venlafaxine). Citalopram, oxazepam, and venlafaxine were therefore common to all three sites and were considered as persistent compounds as they were not totally eliminated during wastewater treatment. The identity of the parent compounds was then confirmed by the injection of the analytical standards in MS/MS mode (level 1) (see the example for oxazepam in WWTP1 raw and treated waters given in Figure S1).

The occurrence of these three compounds in wastewater has been shown by different studies [24,27,31,71,81,82]. The presence of these molecules in raw water can be explained by their excretion via human metabolism. Indeed, after use, these compounds are excreted in both altered and unchanged forms and then transported to WWTPs via urine and feces. A total of 26 to 29% of ingested citalopram is excreted unchanged [28]. For oxazepam, 75% is not metabolized [4,83]. Finally, between 1 and 10% of venlafaxine is excreted unchanged [4,36]. Excretion is therefore a mechanism that may explain the presence of these compounds in raw water, since it introduces a significant amount of citalopram, oxazepam, and venlafaxine into WWTPs. The persistence of these three compounds during wastewater treatment might be explained by the efficiency of the treatment processes. In fact, both citalopram and oxazepam are refractory compounds to wastewater treatment, having a removal rate in a conventional WWTP of 10 and 13%, respectively [32,44]. For venlafaxine, the removal rate for a conventional WWTP is 40%; therefore, more than half of venlafaxine is not removed in WWTPs [41].

Fluoxetine was detected only in raw water from WWTP1. Several studies have demonstrated its occurrence in raw [27,28,84] and treated water [27,28]. The non-detection of fluoxetine in treated water could be explained by elimination during wastewater treatment processes, by the sensitivity of the instrumentation, or by sorption on sewage sludge due to its physicochemical properties [85,86].

Finally, alprazolam and diazepam were not detected in either raw or treated water at any of the WWTPs. The sensitivity of the instrumentation used is in the picogram range [55] and the low concentrations of these compounds in wastewater might therefore explain the non-detection of these compounds in the WWTPs studied. In fact, these compounds had been detected in influent and effluent at low concentrations ranging from 1.88 to 8.8 ng L^−1^ for alprazolam and from 2.2 to 12 ng L^−1^ for diazepam using more sensitive techniques such as triple quadrupole mass spectrometers [16,28,72,82].

#### 3.2.2. Case of the Potential TPs of the Six Selected Drugs

**Figure 2:**
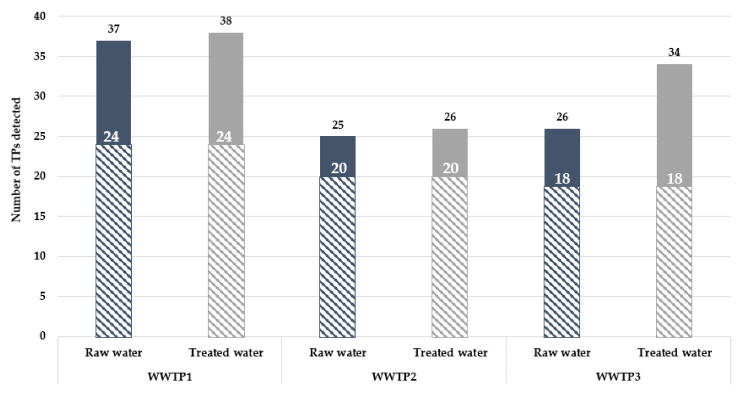
Number of potential TPs in the studied WWTPs. The full bars represent the number of TPs specific to WWTP influents (blue) and effluents (grey), respectively. The number of persistent TPs (found in both influent and effluent waters) for each site is shown as hatched bars.

Among the 251 TPs screened, between 25 and 38 potential TPs were detected (level 4), depending on the WWTP and the type of wastewater (Figure 2). Between 25 and 37 TPs were potentially detected in raw waters and between 26 and 38 in treated waters. WWTP1 was the site with the highest number of potential TPs detected for both influent and effluent.

For each WWTP, potential persistent TPs, i.e., present in both raw and treated waters, were observed; there were 24 in WWTP1, 20 in WWTP2, and 18 in WWTP3. The detection of metabolites at the WWTP inlet might be explained by the excretion of these compounds via urine and feces. Furthermore, during the transport of wastewater to WWTPs, compounds might undergo abiotic reactions to form TPs, also explaining their presence in raw water [87]. Among the persistent features, *O*-desmethylvenlafaxine (ODV) and *N,O*-didesmethylvenlafaxine (NODDV)—two metabolites of venlafaxine—were detected in all three WWTPs. After ingestion of a dose of venlafaxine, 29% is excreted as ODV, 6–19% as NODDV, and 1% as *N*-desmethylvenlafaxine [4,30]. Venlafaxine is a compound with an elimination rate of 40% in a conventional WWTP [32]. The eliminated part of venlafaxine would be transformed into TPs by biodegradation reactions [45,88] and abiotic degradation reactions [88,89,90]. In addition, norcitalopram—a metabolite of citalopram—was also persistent in WWTP1 and WWTP3. When citalopram is ingested and metabolized, 19% of the citalopram dose is excreted as desmethylcitalopram (i.e., norcitalopram) [30].

#### 3.2.3. Comparison of the WWTPs

**Figure 3:**
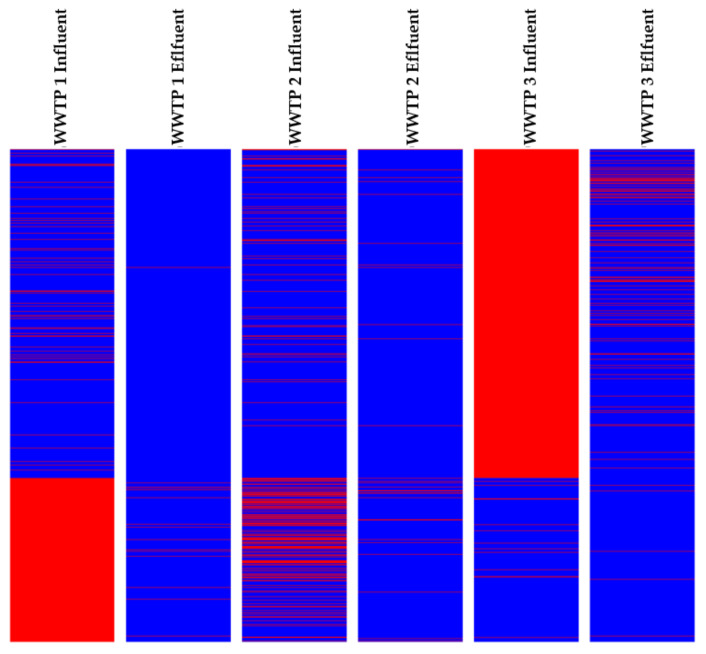
Hierarchical clustering analysis (HCA) for the chemical fingerprint of the 3 WWTPs (MPP software, v.10.0). Each colored line indicates a chemical entity. The relative abundance of each entity depends on the color, from red (high abundance) to blue (low or null abundance) [91].

After the detection of the psychotropic drugs and their potential TPs in the samples, a comparison between WWTP1, WWTP2, and WWTP3 was carried out using chemical fingerprinting, as illustrated in the heatmap (Figure 3).

Thus, this chemical fingerprint comprising all the entities present in the samples (10,847 features) informed us, through color shifts, of three possible trends that the features and thus potential TPs in WWTPs might follow: elimination during wastewater treatment, formation of TPs by treatment processes, and non-elimination of compounds in WWTPs. Thus, the classification by color change allowed to form clusters of features following the three different behaviors throughout water treatments. The color shift also indicated that some entities are common or specific to a site and, therefore, to a wastewater treatment process. For further analysis and compound identification, feature lists can be retrieved for the specific clusters of the data analysis and visualization technique (heatmap).

After the data processing treatment of the detected features, 60 potential TPs/metabolites related to the 6 monitored psychotropic drugs were proposed. Then, they were classified according to their behavior in the samples (Table 3). The average retention time, accurate mass (*m*/*z*), and mass error for each entity corresponding to potential TPs are shown in Table S7.

#### 3.2.4. Potential Persistent TPs of the Six Selected Drugs

Nine potentials TPs were classified as ubiquitous and persistent because they were detected in the raw and treated waters of the three WWTPs. The different wastewater treatments (one- or two-stage biofiltration, UV disinfection, and fixed cultures) did not eliminate these TPs. In consideration of their behavior, these compounds were considered relevant. Among these molecules, two potential TPs of oxazepam (TP-OXA-12 and TP-OXA-14) and seven TPs of venlafaxine (ODV, NODDV, venlafaxine met 5, venlafaxine TP23, TP-VFX-25a, and VB4, TP 216) were detected (Table 3).

**Figure 4:**
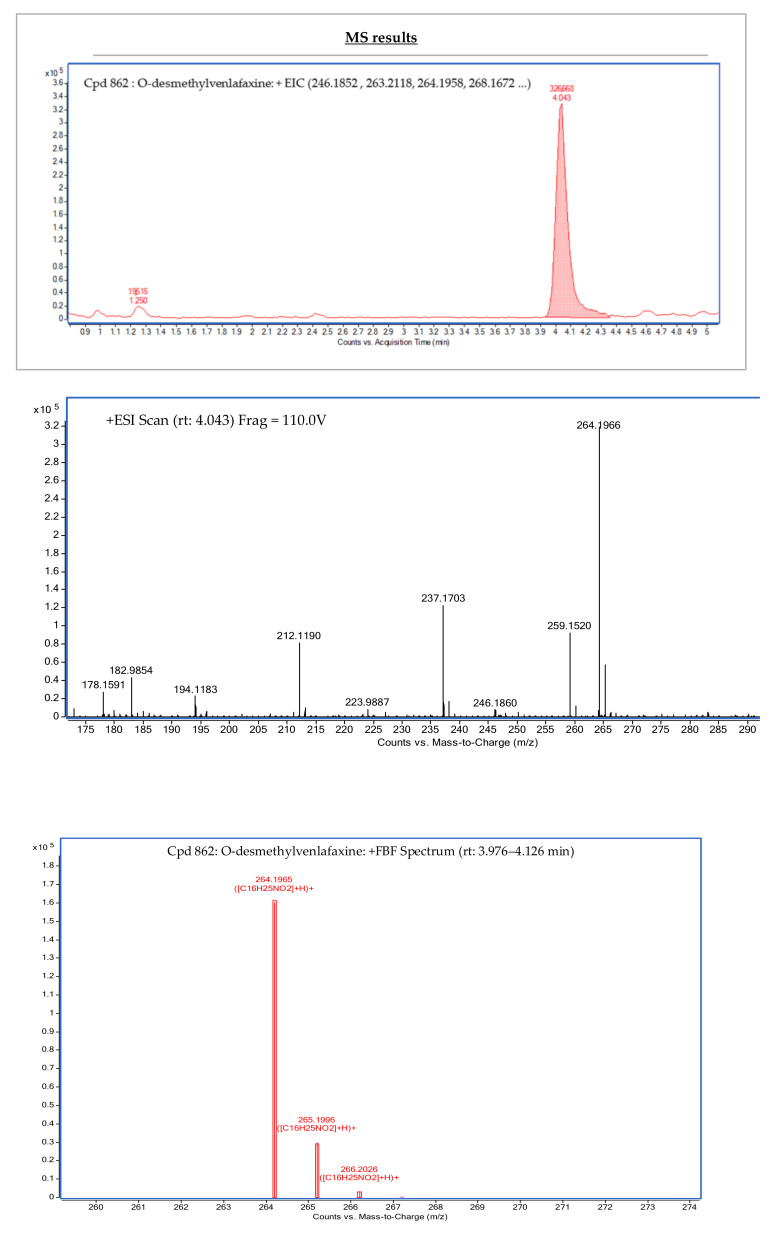

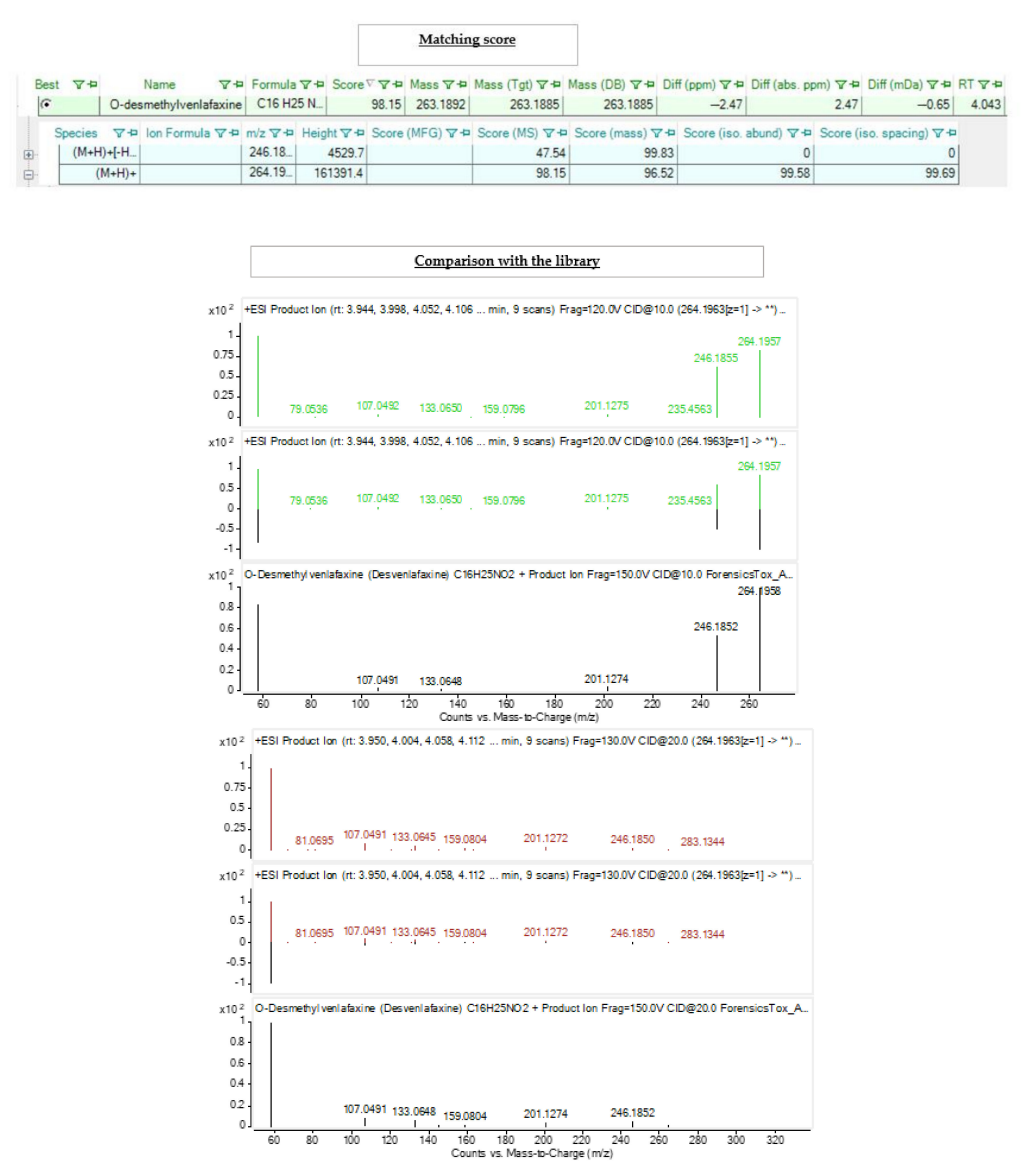

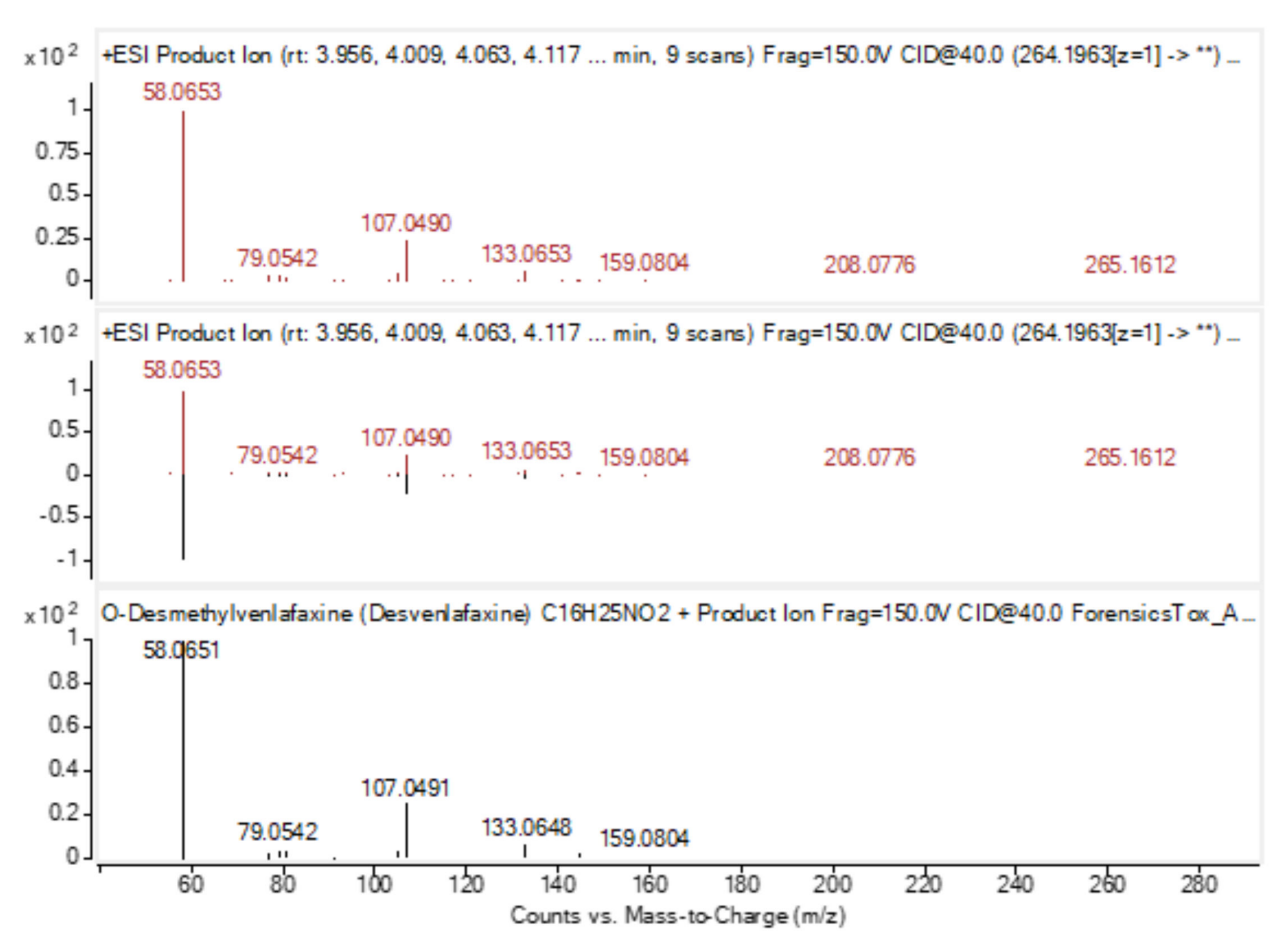
Chromatogram and experimental MS^2^ spectra obtained for ODV (identified at level 2a) in WWTP1 effluent. ** represents the unknown fragment ions generated during the fragmentation of the precursor ion 264.1963.

Following MS/MS experiments on these selected TPs, the identity of ODV and NODDV (level 2a) has been validated by library matching in the raw and treated waters of WWTPs. Comparison and fit of the fragmentation spectra at three collision energies of ODV in the WWTP1 effluent are shown in Figure 4. The occurrence of ODV (C_16_H_25_NO_2_; [M + H]^+^ *m*/*z* = 264.1961, RT = 4.00 min), NODDV (C_15_H_23_NO_2_; [M + H]^+^ *m*/*z* = 250.1803, RT = 3.70 min), and VB4 (C_17_H_23_NO_2_; [M + NH_4_]^+^ *m*/*z* = 291.2070, RT = 10.03 min) in wastewater have been reported previously in different studies [6,26,31,45]. The presence and identity of VB4 could not be confirmed with a higher level of confidence because fragmentation spectra were not obtained, probably due to the sensitivity of the instrumentation used. Norcitalopram (C_19_H_19_FN_2_O; [M + H]^+^ *m*/*z* = 311.1558, RT = 12.12 min)—a metabolite of citalopram—was persistent in WWTP1 and WWTP3; in WWTP2, it was only detected in the treated water. This non-detection in the raw water of WWTP2 might be explained by the presence of matrix effects that could suppress the signal and by the sensitivity of the instrumentation. The presence of norcitalopram in wastewater has been reported in several studies [38,39,40]. The presence and identity of norcitalopram was also confirmed at level 2a by library (and RT) matching.

TP 216 (C_11_H_23_NO_3_; [M + H]^+^ *m*/*z* = 216.1595, RT = 12.76 min)—a TP of venlafaxine—has already been detected in water [51]. It appeared to be generated by advanced oxidation reactions during experiments using UV/H_2_HO_2_ treatment [51]. The detection of venlafaxine met 5 (C_16_H_25_NO_3_; [M + H]^+^ *m*/*z* = 280.1909, RT = 2.39 min) and venlafaxine-TP23 (C_14_H_21_NO_2_*;* [M + H]^+^ *m*/*z* = 236.1648, RT = 2.58 min) in wastewater has never been reported in the literature. Venlafaxine TP-23, also searched [73], has not been detected in river water. If the molecular features corresponding to these potential TPs had been well fragmented in AutoMSMS, the identity of these compounds could not be validated with a higher confident level due to the lack of spectra in the library. Exemplary chromatogram and MS^2^ spectra are shown for venlafaxine met 5 in the effluent of WWTP1 (Figure S2).

The detection of TP-VFX-25a (C_15_H_23_NO_2_; [M + H]^+^ *m*/*z* = 250.1805, RT = 2.48 min) in wastewater has also never been reported in the scientific literature. This TP was obtained from the Excel spreadsheet developed from EAWAG-BDD/PPS and might therefore be generated by didemethylation or by desethylation. However, the detected molecular entity could correspond to either TP-VFX-25a, *O*- and *N*-desmethyltramadol [74], or NNDV; the fragmentation spectra matched those of O-desmethyltramadol, which is thus the persistent TP identified.

Lastly, the presence of TP-OXA-12 and TP-OXA-14 in wastewater has never been mentioned in the scientific literature. These two compounds were also generated by the Excel spreadsheet. TP-OXA-12 (C_15_H_14_N_2_O_2_; [M + H]^+^ *m*/*z* = 255.1130, RT = 7.48 min) is produced by a reductive displacement of chlorine and hydrogenation, while TP-OXA-14 (C_15_H_14_N_2_O_3_; [M + H]^+^ *m*/*z* = 271.1078, RT = 6.27 min) is generated by a combination of a reductive displacement of chlorine and hydration. Here again, their fragmentation spectra allowed us to identify these entities in our internal libraries as being 10,11-dihydro-10-hydroxycarbamazepine (level 2a) [92] and CAR_270 (level 2a), a TP of carbamazepine, instead of TP-OXA-12 and TP-OXA-14, respectively. These persistent TPs were thus those of carbamazepine. As an example, the match of the experimental spectra with those of CAR_270 is represented in Figure S3 for the WWTP2 effluent.

#### 3.2.5. Potential TPs of the Six Selected Drugs Present in Raw Water Only

The name and the elemental composition of some possible TPs specific to raw water could be proposed using the classification presented in the Table 3. A potential fluoxetine TP generated by the Excel spreadsheet, TP-FLX-67a (C_17_H_19_NO_3_; [M + H]^+^ *m*/*z* = 286.1441, RT =22.98 min), was detected in the raw water of the three WWTPs. To our knowledge, this compound has never been reported in the literature. The non-detection of TP-FLX-67a in treated water means that this compound might have been eliminated by the biological treatment of wastewater, since it was also removed by WWTPs that perform biological treatment. Possible TPs specific to WWTP raw water were also detected and proposed based on elemental composition. For instance, a TP of alprazolam generated by the Excel spreadsheet, TP-ALP-50 (C_17_H_16_N_4_O; [M + H]^+^ *m*/*z* = 293.1377, RT = 10.40 min), was detected only in influents from WWTP 1 and was probably removed during the two-stage biofiltration biological treatment used at that site. However, the identity of these TPs was based only on their accurate masses and, thus, on their elemental compositions (these potential TPs were not selected for MS/MS experiments at this step).

#### 3.2.6. Potential TPs of the Six Selected Drugs Formed during Water Treatments (Present in Treated Water Only)

Finally, the occurrence, as well as the potential identity, of TPs present only in the treated waters of WWTPs was proposed. TP-FLX-24c (C_16_H_17_F_2_NO_2_*;* [M + NH4]^+^ *m*/*z* = 311.1559, RT = 12.09 min), generated by the Excel spreadsheet and produced by a reaction of oxidative displacement of fluorine followed by a reaction of demethylation, was detected in the treated water of the three WWTPs investigated. The TP-FLX-24c seemed to be generated during the biological treatment, a common parameter of these three WWTPs. Moreover, one TP, never reported in the literature, common to the WWTP1 and WWTP2 effluents was detected; it was a TP of citalopram, TP-CTR-44 (C_20_H_24_N_2_O_2_*;* [M + H]^+^ *m*/*z* = 325.1917, RT = 3.84 min), also generated by the in silico prediction program. It could be formed by a reductive displacement of fluorine followed by hydration or by an oxidative reductive displacement of fluorine combined with a hydrogenation reaction. The presence of the compound in the treated water of WWTP1 and WWTP2 could mean that TP-CTR-44 was generated during the biofiltration treatment. Its non-detection in the treated water of WWTP3 could be due to the use of tertiary treatment (UV disinfection). This hypothesis remains to be confirmed or refuted in future studies and analyses such as laboratory degradation experiments of citalopram (parent compound of this TP) in WWTP water. In addition, two potential TPs—TP-VFX-28 (C_16_H_23_NO; [M + H]^+^ *m*/*z* = 246.1846, RT = 3.99 min) and venlafaxine-F1 (C_17_H_25_NO, [M + H]^+^ *m*/*z* = 260.2009, RT = 8.32 min)—were detected in the treated water of WWTPs 1 and 3. TP-VFX-28 was produced by two reactions, demethylation and dehydration. Venlafaxine-F1 was already screened in WTWPs effluents [70] but was not detected. Thus, to our knowledge, these TPs were never found in wastewater. Finally, one TP of fluoxetine—TP163 (C_10_H_13_NO, [M + H]^+^ *m*/*z* = 164.1069, RT = 6.36 min)—was detected in the treated water of WWTP2 and WWTP3. This compound was previously described [75] and was obtained by ozonation of fluoxetine and, in particular, by disruption of the C-O bond.

Therefore, MS/MS analyses were performed to increase the confident level in identification and to confirm the occurrence of these relevant TPs. So, the identity of specific TPs to treated water could be proposed on the basis of the elemental composition, MS spectrum, and MS^2^ spectrum. Fragment spectra were acquired for TP-CTR-44, TP-VFX-28, Venlafaxine-F1, and TP163 (at 10, 20, and 40 eV) but not for TP-FLX-24c. The identity of the first three TPs could not be confirmed with a higher confident level due to the lack of spectra in the libraries. Exemplary chromatogram and MS^2^ spectra are shown for TP-VFX-28 in WWTP1 effluent (Figure S4).

TPs specific to the treated water of each WWTP could also be suggested according to their accurate masses, elemental composition, and MS^2^ spectrum when available. Thus, the potential formation of TPs by different wastewater treatment processes was determined.

Seven unique TPs (TP-ALP-43, TP-CTR-45, TP-DIA-70, TP-OXA-22d, venlafaxine TP16b, venlafaxine TP16c, and venlafaxine TP32) were detected in the treated water of WWTP1. Thus, they were probably generated during the two-stage biofiltration biological treatment. Among these seven TPs, TP-ALP-43, TP-CTR-45, TP-DIA-70, and TP-OXA-22d were from the Excel spreadsheet and were formed by different abiotic reactions. The occurrence of venlafaxine-TP32 (C_15_H_20_O_4_, *m*/*z* = 282.1710, RT = 2.43 min) and two isomers of venlafaxine TP16, venlafaxine TP16b (C_14_H_18_O_4_; [M + H]^+^ *m*/*z* = 251.1284, RT = 2.30 min), and venlafaxine TP16c (C_14_H_18_O_4_; [M + H]^+^ *m*/*z* = 251.1284, RT = 8.39 min) in wastewater have also never been mentioned in the literature. Venlafaxine TP-16—already investigated [73]—was not detected in river water. Moreover, venlafaxine TP32 was not detected in raw water [74].

One specific TP—venlafaxine TP26 (C_6_H_10_O; [M + NH4]^+^ *m*/*z* = 116.1073, RT = 0.82 min)—was identified in the treated water of WWTP2. It was already screened but not found [84]. This presence was detected in wastewater for the first time to our knowledge. Venlafaxine TP26 was probably produced during biological treatment by biofiltration on a stage. It was not found in WWTP1, which has a two-stage biological treatment. The presence of two filtration stages probably allowed the elimination of this molecule.

For WWTP 3, three TPs (TP-ALP-11, TP-VFX-17, and venlafaxine TP31) were reported only in the treated water. Hence, these TPs were certainly and specifically generated by the UV treatment, as they were only present in the effluent from WWTP3. TP-ALP-11 (C_15_H_9_ClN_4_; [M + H]^+^ *m*/*z* = 281.0598, RT = 12.55 min) and TP-VFX-17 (C_17_H_27_NO_4_; [M + H]^+^ *m*/*z* = 310.2011, RT =2.61 min) were computer-generated and were never described in the literature. TP-ALP-11 was formed by didemethylation or desethylation of alprazolam, while TP-VFX-17 could be produced by several combinations of reactions, such as two consecutive hydroxylation reactions. Finally, the presence of venlafaxine-TP31 (C_14_H_18_O_3_; [M + NH4]^+^ *m*/*z* = 252.1590, RT = 6.10 min) in raw waters was investigated [74], but this compound was not found in the analyzed samples.

The identity and occurrence of five TPs (venlafaxine TP16c, venlafaxine TP32, venlafaxine TP26, TP-ALP-11, and venlafaxine TP31) could not be validated with a higher level of confidence because fragmentation spectra were not obtained. Fragmentation spectra have been acquired for TP-ALP-43, TP-CTR-45, TP-DIA-70, TP-OXA-22d, venlafaxine TP16b, and TP-VFX-17 at three different collision energies (10, 20, and 40 eV). Unfortunately, the identity of these compounds could not be confirmed with a level 2 due to the lack of spectra in the libraries. Moreover, the entity corresponding to TP-OXA-22d, as well as to other carbamazepine TPs, was finally identified at level 2a as carbamazepine-10,11-epoxide (Figure S5). This TP is therefore not a TP of oxazepam but a metabolite of carbamazepine.

## 4. Conclusions

The analytical methodology developed in this work allowed the detection of some psychotropic compounds and several of their potential TPs in wastewater. Four out of the six monitored drugs—citalopram, fluoxetine, oxazepam, and venlafaxine—were detected in at least one of the WWTPs samples. The number of their potential TPs detected varied between 25 and 38 depending on the WWTP and the water sampled. Certain TPs, such as desmethylvenlafaxine or norcitalopram, were identified and found to be ubiquitous, while others such as TP-VFX-17, venlafaxine-TP-26, TP-DIA-70, or TP-CTR-44 were formed during the wastewater treatment, although their identity was still uncertain (level 4–5).

The use of predictive software coupled with a bibliographic review allowed us to search for a high number of known and unknown TPs. This method, mainly based on HRMS full scan mode, is a precursor tool that can be used to detect potential TPs. In addition, the use of filters during data processing proved to be necessary to reduce the number of entities extracted by the software, thus reducing the number of false positives. In addition, the use of MS/MS mode on the TPs considered most relevant was necessary because it allowed us to not only confirm the occurrence of TPs in WWTPs but also, in certain cases, to improve the level of confidence in the identification of the TPs, confirming or refuting the identities proposed from the accurate masses and elemental compositions. Moreover, this mode allowed us to obtain fragmentation spectra of potential TPs not previously reported in the literature. However, as mentioned before, analyses in MS/MS mode were only performed on ubiquitous non-eliminated TPs and TPs potentially generated by WWTPs. Moreover, the short fragmentation study rather led to the identification of several TPs of carbamazepine as ubiquitous persistent TPs.

In the near future, additional analyses will be performed to proceed further in the identification of the proposed TPs. This method (that needs to be perfected) gave very promising results and proved useful and feasible in complex matrices such as wastewater. The results obtained and this analytical strategy may be very useful and serve as a starting point for future studies interested in the identification and, especially, the elucidation of TP structures.

## Figures and Tables

**Table 1:** Characteristics of the 3 investigated WWTPs.

Code	Capacity (Eq. Inh.)	Output Rate (m^3^·d^−1^)	Type of Secondary Treatment	Tertiary Treatment
WWTP1	85,000	18,700	Two-stage biofiltration	None
WWTP2	366,000	210,000	One-stage biofiltration	None
WWTP3	150,000	25,000	Bacterial beds	UV disinfection *

* UV disinfection in WWTP3 only takes place between May and September.

**Table 2:** Information on the 6 psychotropic drugs and numbers of their associated TPs.

Compound	Formula	Mass	TPs Referenced in the Literature	TPs Generated In Silico (Excel Macro)
Alprazolam	C_17_H_13_ClN_4_	308.0828	3	27
Citalopram	C_20_H_21_FN_2_O	324.1638	12	35
Diazepam	C_16_H_13_ClN_2_O	284.0716	18	22
Fluoxetine	C_17_H_18_F_3_NO	309.1340	10	30
Oxazepam	C_15_H_11_ClN_2_O_2_	286.0509	11	18
Venlafaxine	C_17_H_27_NO_2_	277.2042	50	15

**Table 3:** Classification of the entities being possibly TPs according to their occurrence in WWTP samples. Red indicates that potential TP was not detected. Green indicates that potential TP was detected.

	WWTP1		WWTP2		WWTP3	
Compounds	Influent	Effluent	Influent	Effluent	Influent	Effluent
TP-ALP-11						
TP-ALP-17						
TP-ALP-28						
TP-ALP-43						
TP-ALP-50						
TP-ALP-51						
Desmethylcitalopram						
TP-CTR-9						
TP-CTR-44						
TP-CTR-45						
TP-DIA-47						
TP-DIA-59a/1,3-Dihydro-5-phenyl-2H-1,4-benzodiazepin-2-one						
TP-DIA-59b/Carbamazepine (parent)						
TP-DIA-70						
TP 163						
TP165						
TP298						
TP325						
TP-FLX-16						
TP-FLX-24a						
TP-FLX-24b						
TP-FLX-24c						
TP-FLX-57						
TP-FLX-67a						
TP-FLX-67b						
OXZ-VI						
TP-OXA-7						
TP-OXA-12/10-hydroxycarbazepine						
TP-OXA-14/CAR_270b/trans-10,11-dihydroxy-10,11-dihydrocarbazepine						
TP-OXA-22a/Carbamazepine 10,11 epoxide/Oxcarbazepine/1,/3,4-hydroxycarbamazepine						
TP-OXA-22b/Carbamazepine 10,11 epoxide/Oxcarbazepine/1,/3,4-hydroxycarbamazepine						
TP-OXA-22c/Carbamazepine 10,11 epoxide/Oxcarbazepine/1,/3,4-hydroxycarbamazepine						
TP-OXA-22d/Carbamazepine 10,11 epoxide/Oxcarbazepine						
ODV						
NODDV						
Venlafaxine-F1						
Venlafaxine met 5/Tramadol-N-Oxide						
Venlafaxine met 9						
Venlafaxine TP16a						
Venlafaxine TP16b						
Venlafaxine TP16c						
Venlafaxine TP23						
Venlafaxine TP26						
Venlafaxine TP31						
Venlafaxine TP32						
VB3						
VB4						
TP 216						
TP-VFX-15						
TP-VFX-16/Venlafaxine-N-Oxide						
TP-VFX-17						
TP-VFX-25a/O-desmethyltramadol/N-desmethyltramadol/NNDDV						
TP-VFX-25b/O-desmethyltramadol/N-desmethyltramadol/NNDDV						
TP-VFX-25c/O-desmethyltramadol/N-desmethyltramadol/NNDDV						
TP-VFX-28						
TP-VFX-29						
TP-VFX-34						
TP-VFX-35						
TP-VFX-36						
TP-VFX-42						

## Data Availability

Major data and methods are given in the manuscript and Supplementary Materials; raw data (chromatography mass spectrometry data) are available on request.

## Supplementary Materials

The following supporting information can be downloaded at: https://www.mdpi.com/article/10.3390/toxics11080713/s1. Table S1: List of TPs generated from alprazolam. Table S2: List of TPs generated from citalopram. Table S3: List of TPs generated from diazepam. Table S4: List of TPs generated from fluoxetine. Table S5: List of TPs generated from oxazepam. Table S6: List of TPs generated from venlafaxine. Table S7: Average retention time, accurate mass (*m*/*z*) and mass error of potentials TPs detected in raw and treated water from WWTPs. Figure S1: Exemplary comparison of analytical standard and experimental MS2 spectra for oxazepam. Figure S2: Chromatogram and MS2 spectra of venlafaxine met 5. Figure S3: Chromatogram and MS2 spectra of TP-OXA-14. Figure S4: Chromatogram and MS2 spectra of TP-VFX-28. Figure S5: Chromatogram and MS2 spectra of TP-OXA-22d.
