# Supplementary material for: Assessment of the Presence of Transformation Products of Certain Pharmaceutical Products (Psychotropic Family) by Suspect and Non-Targeted HRMS Screening in Wastewater Treatment Plants
Source: Toxics. 2023 Aug 18;11(8):713. doi: 10.3390/toxics11080713 (PMC10457822; doi:10.3390/toxics11080713)
Supplement: Supplementary file 1 [file toxics-11-00713-s001.zip › toxics-2311378-supplementary.pptx]

## Slide 1
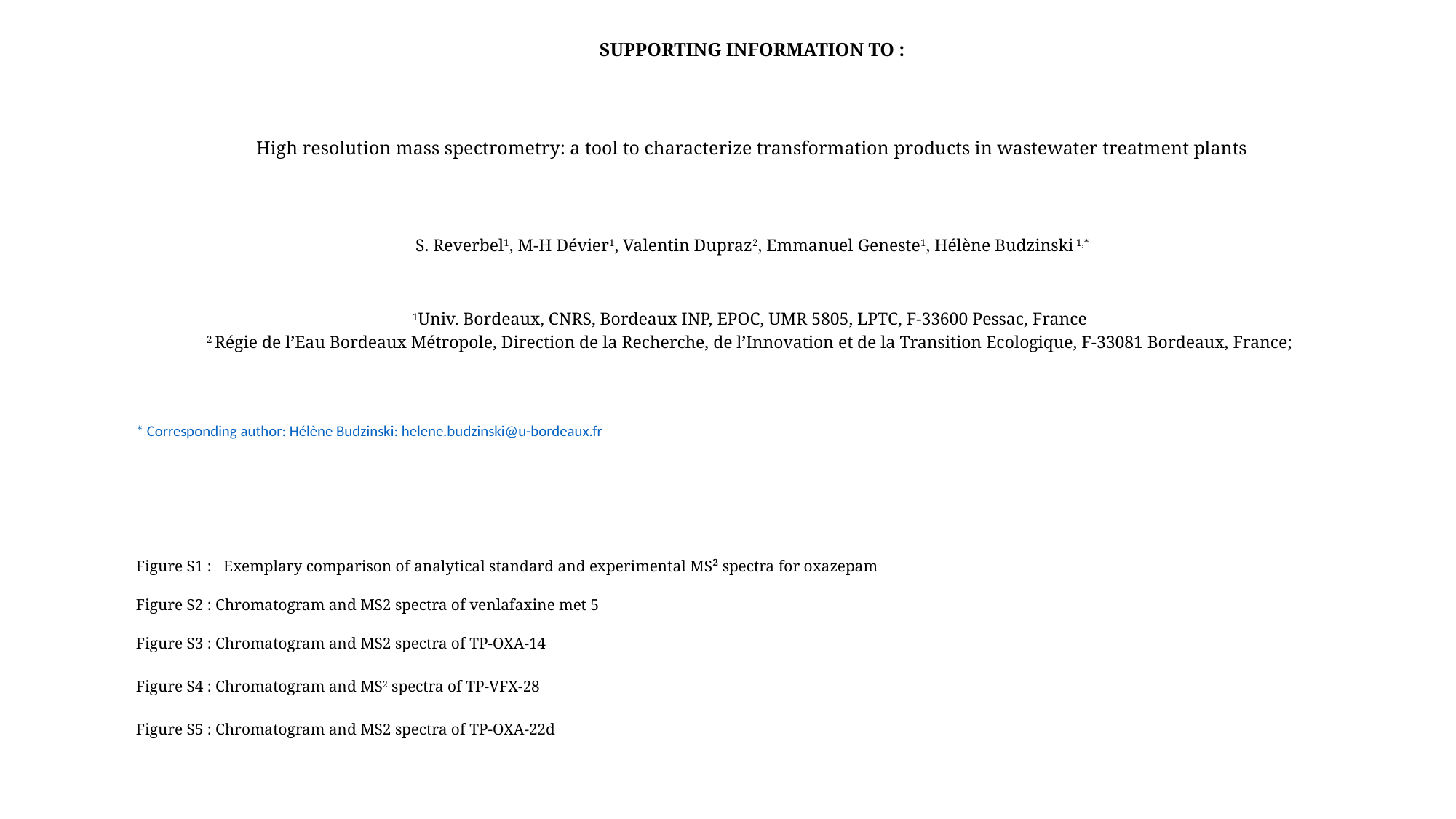

| SUPPORTING INFORMATION TO : |
| --- |
| |
| High resolution mass spectrometry: a tool to characterize transformation products in wastewater treatment plants |
| |
| S. Reverbel1, M-H Dévier1, Valentin Dupraz2, Emmanuel Geneste1, Hélène Budzinski 1,\* |
| |
| 1Univ. Bordeaux, CNRS, Bordeaux INP, EPOC, UMR 5805, LPTC, F-33600 Pessac, France 2 Régie de l’Eau Bordeaux Métropole, Direction de la Recherche, de l’Innovation et de la Transition Ecologique, F-33081 Bordeaux, France; |
| |
| \* Corresponding author: Hélène Budzinski: helene.budzinski@u-bordeaux.fr |
| |
| |
| Figure S1 : Exemplary comparison of analytical standard and experimental MS² spectra for oxazepam |
| Figure S2 : Chromatogram and MS2 spectra of venlafaxine met 5 |
| Figure S3 : Chromatogram and MS2 spectra of TP-OXA-14 |
| Figure S4 : Chromatogram and MS2 spectra of TP-VFX-28 |
| Figure S5 : Chromatogram and MS2 spectra of TP-OXA-22d |

## Slide 2
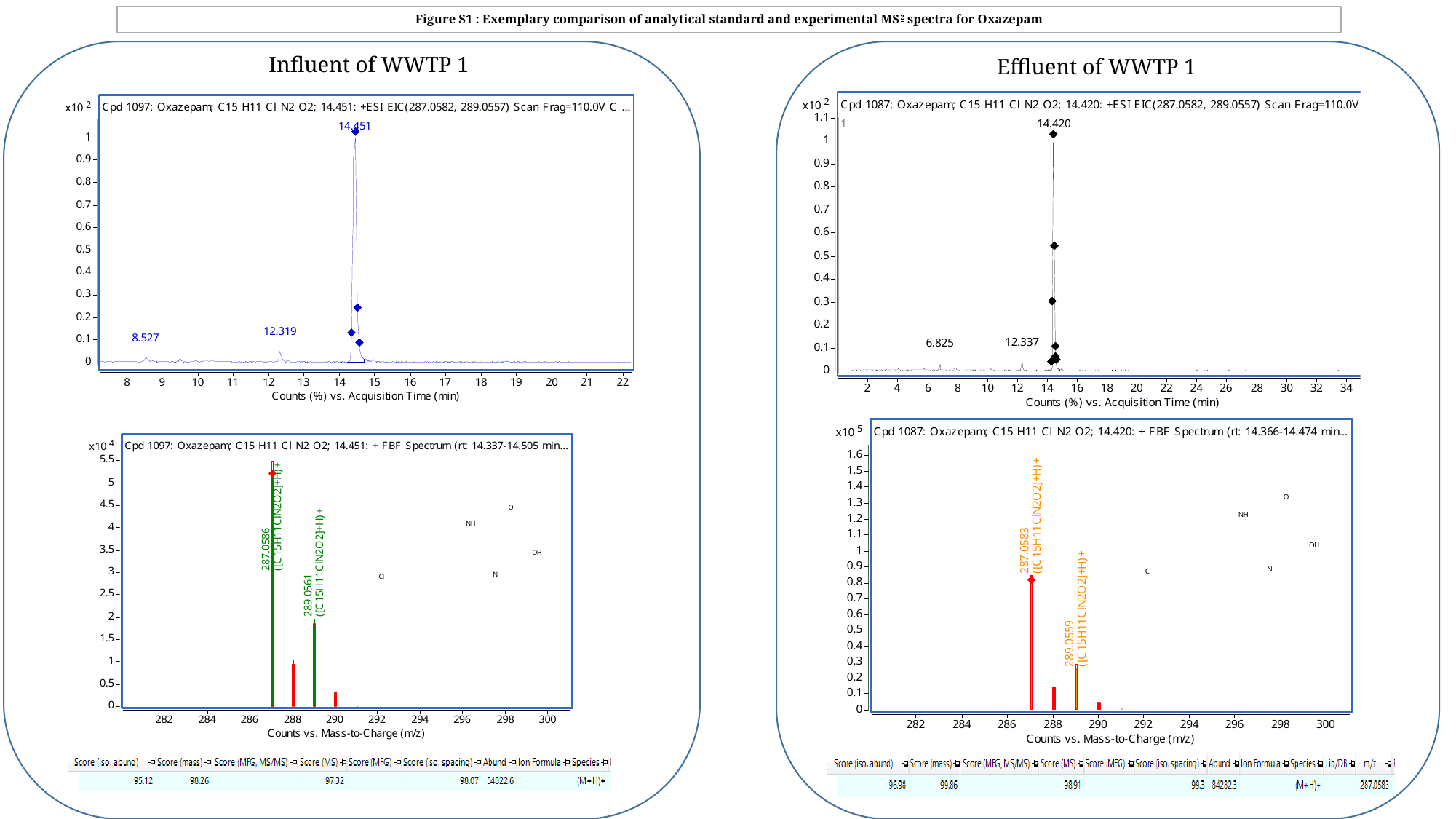

Figure S1 : Exemplary comparison of analytical standard and experimental MS2 spectra for Oxazepam
Influent of WWTP 1
Effluent of WWTP 1

## Slide 3
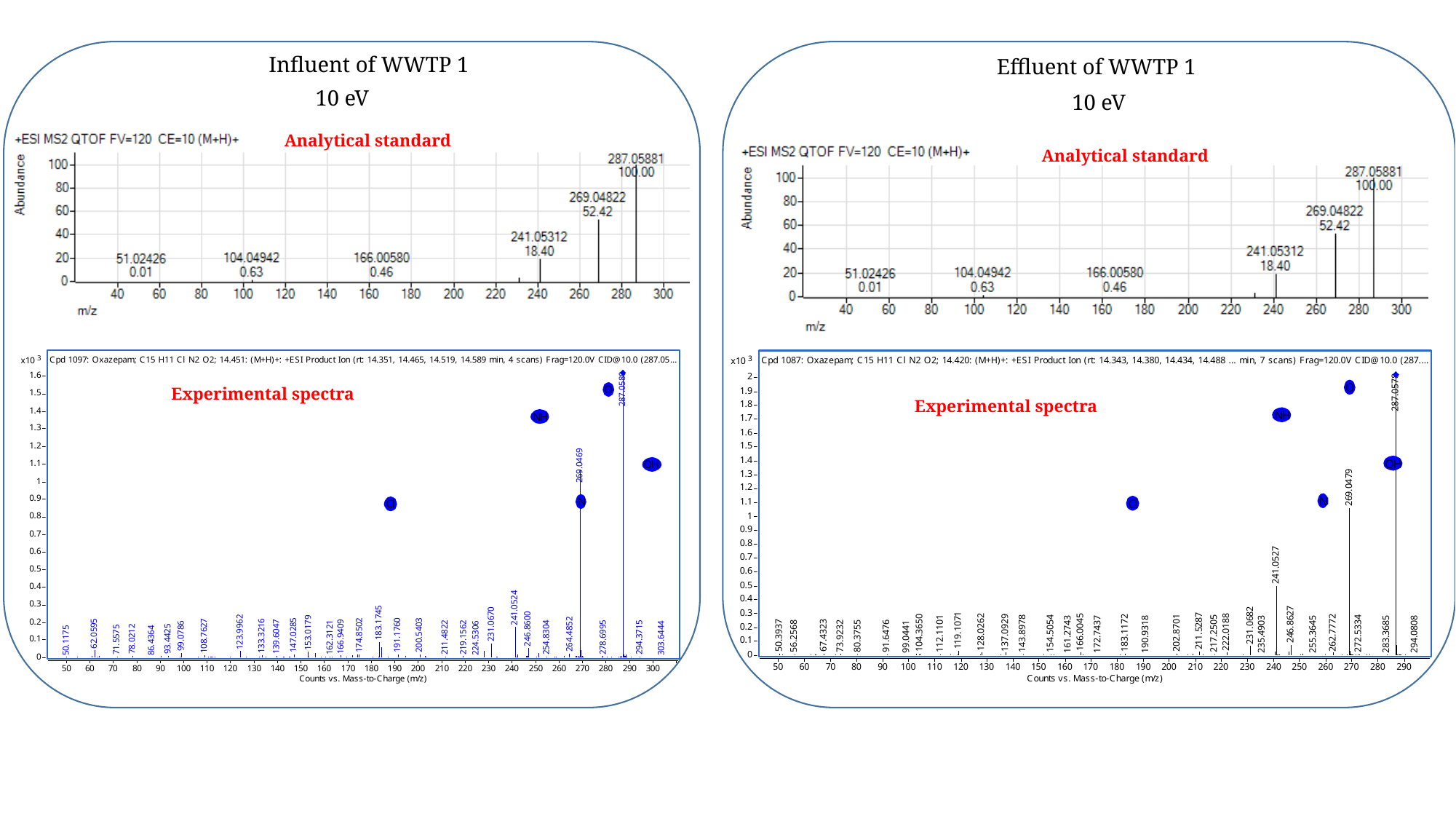

Influent of WWTP 1
Effluent of WWTP 1
10 eV
10 eV
Analytical standard
Analytical standard
Experimental spectra
Experimental spectra

## Slide 4
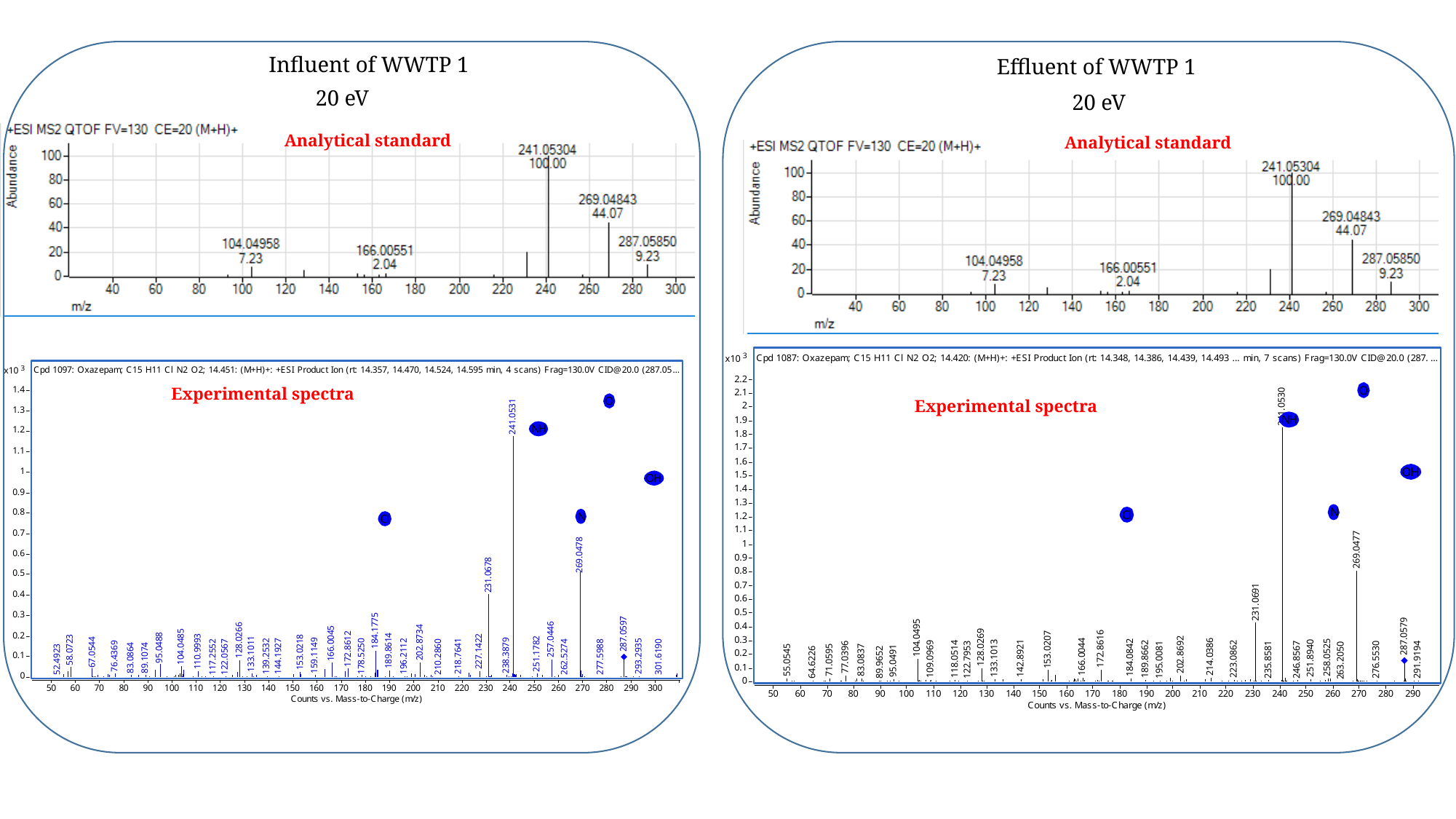

Influent of WWTP 1
Effluent of WWTP 1
20 eV
20 eV
Analytical standard
Analytical standard
Experimental spectra
Experimental spectra

## Slide 5
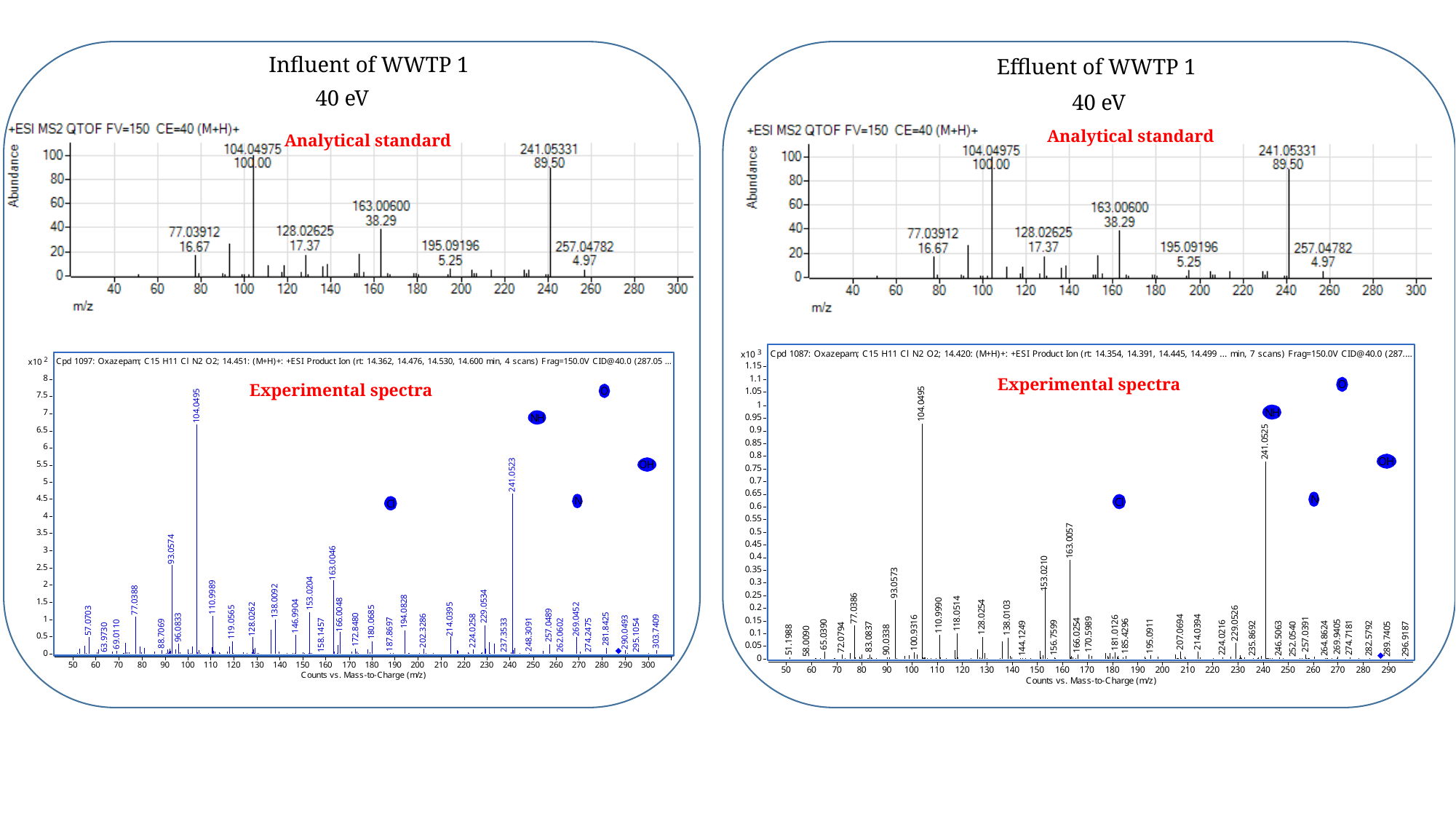

Influent of WWTP 1
Effluent of WWTP 1
40 eV
40 eV
Analytical standard
Analytical standard
Experimental spectra
Experimental spectra

## Slide 6
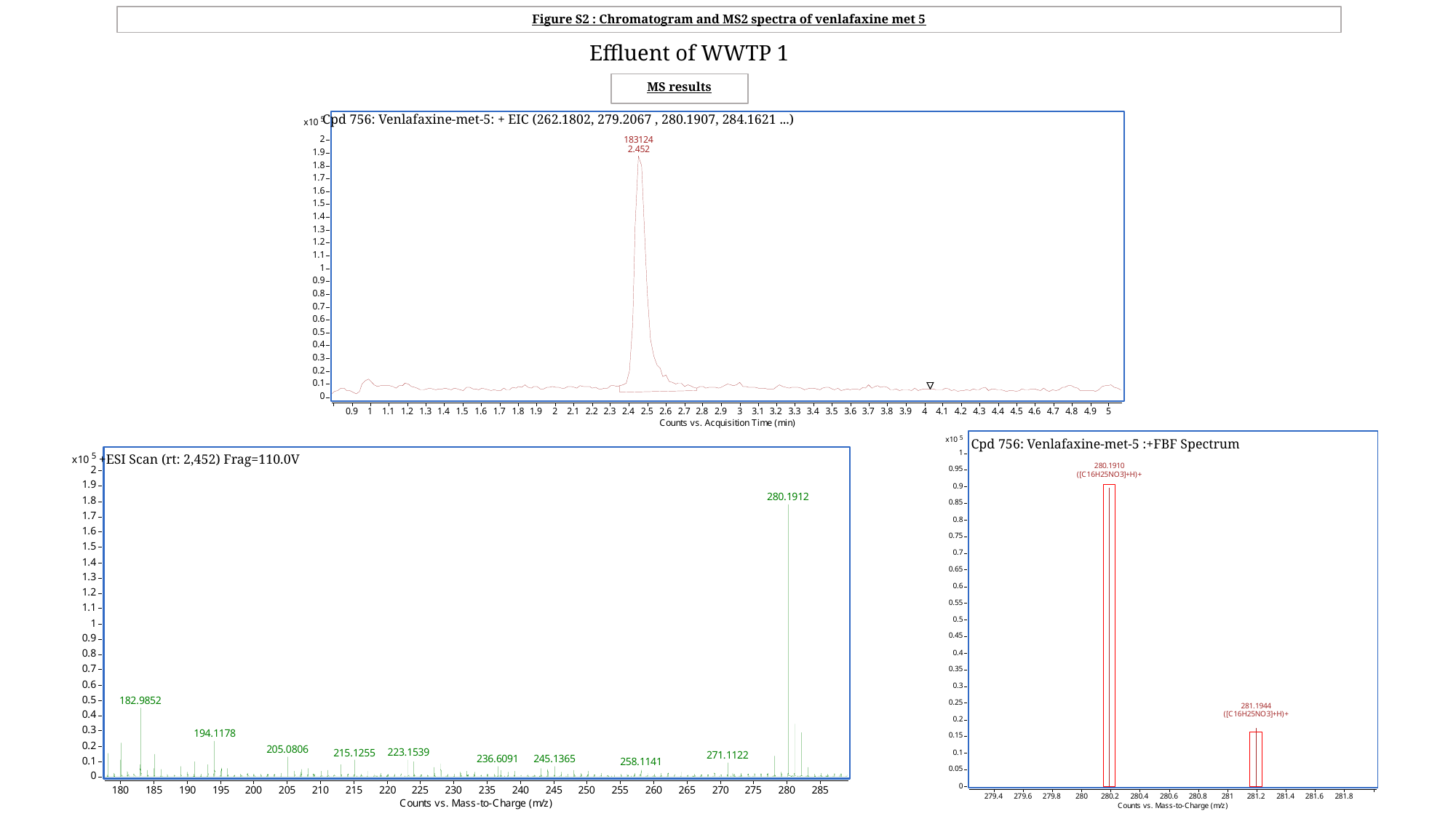

Figure S2 : Chromatogram and MS2 spectra of venlafaxine met 5
Effluent of WWTP 1
MS results
Cpd 756: Venlafaxine-met-5: + EIC (262.1802, 279.2067 , 280.1907, 284.1621 ...)
Cpd 756: Venlafaxine-met-5 :+FBF Spectrum
+ESI Scan (rt: 2,452) Frag=110.0V

## Slide 7
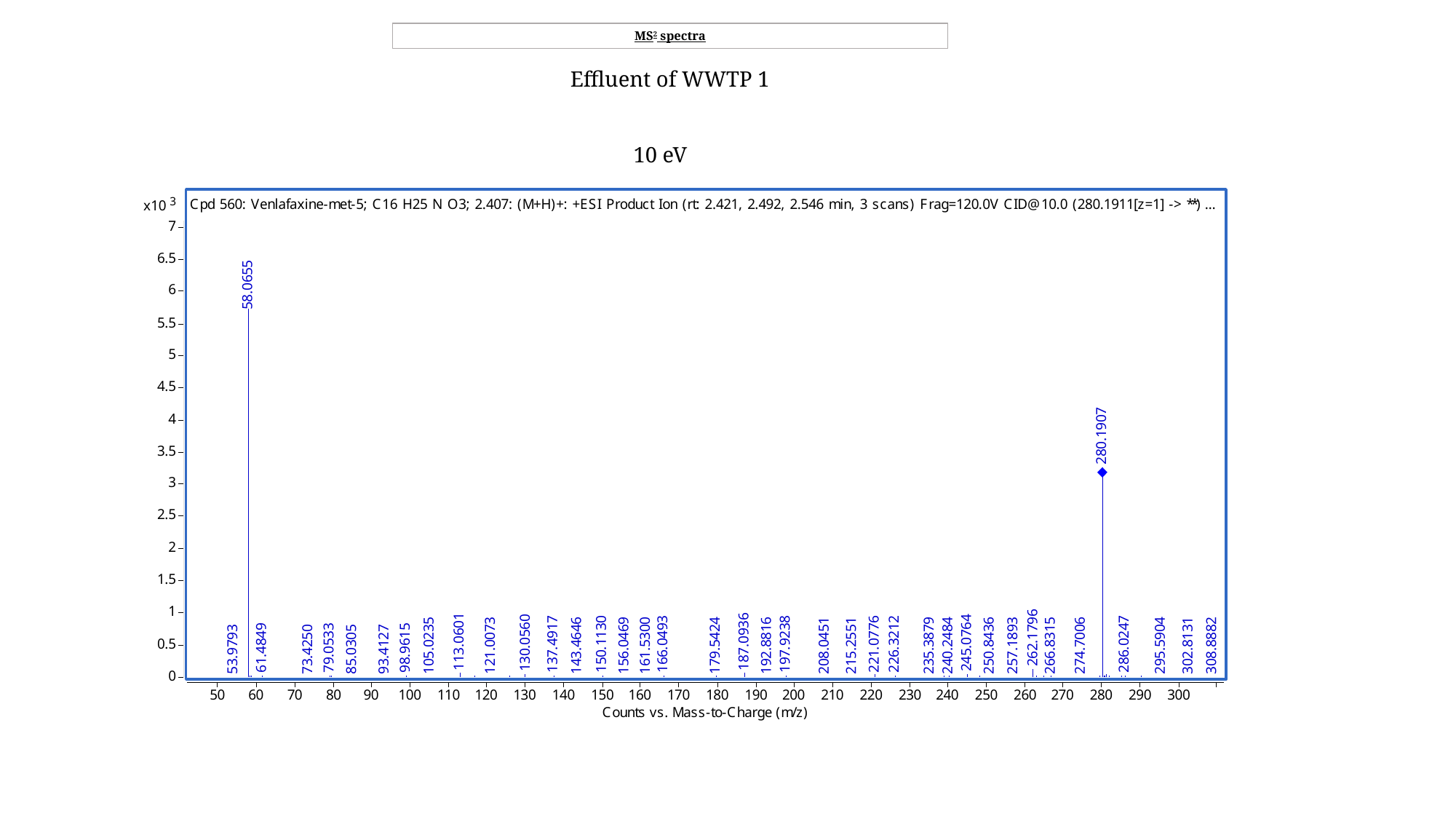

MS2 spectra
Effluent of WWTP 1
10 eV

## Slide 8
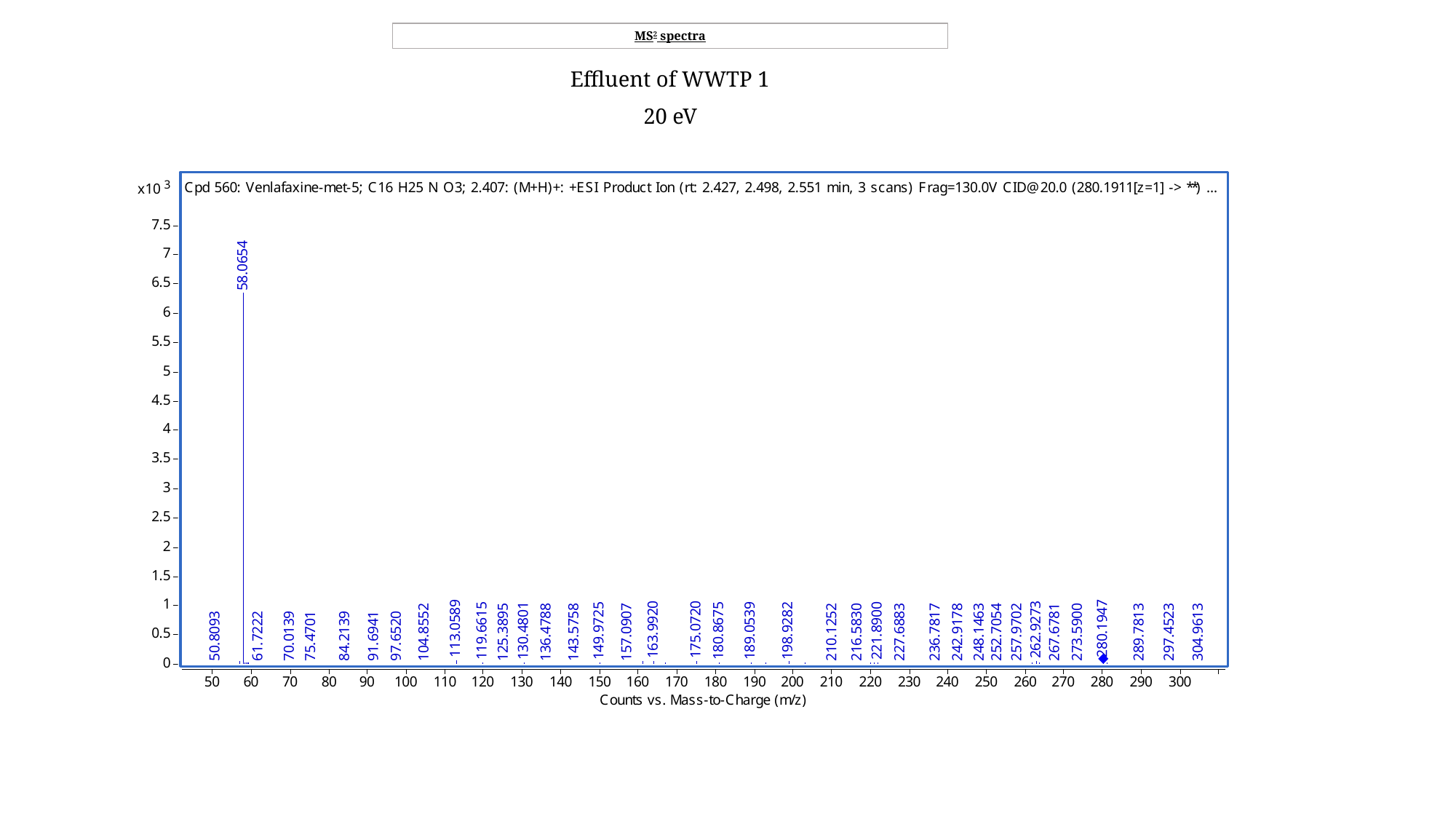

MS2 spectra
Effluent of WWTP 1
20 eV

## Slide 9
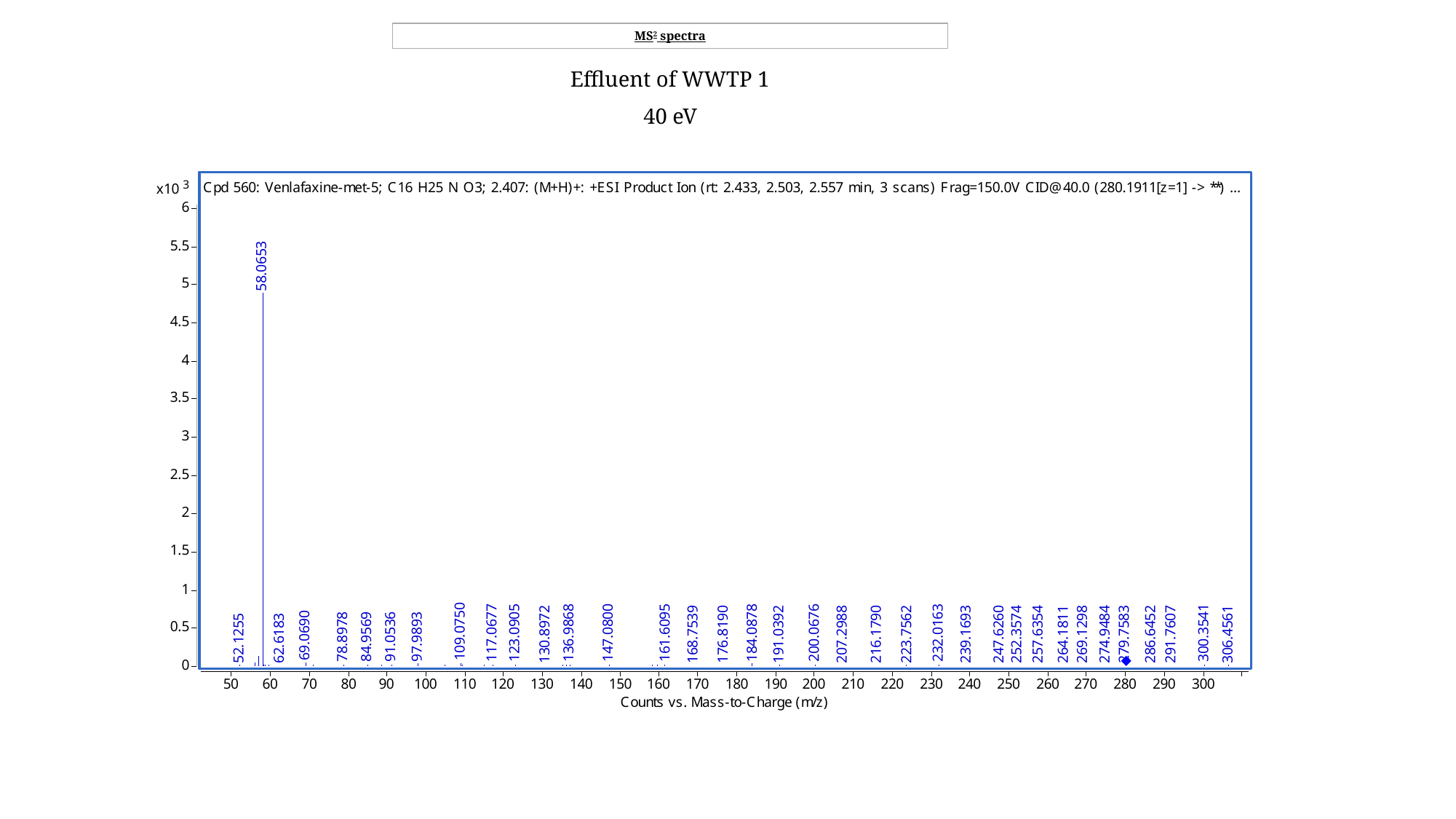

MS2 spectra
Effluent of WWTP 1
40 eV

## Slide 10
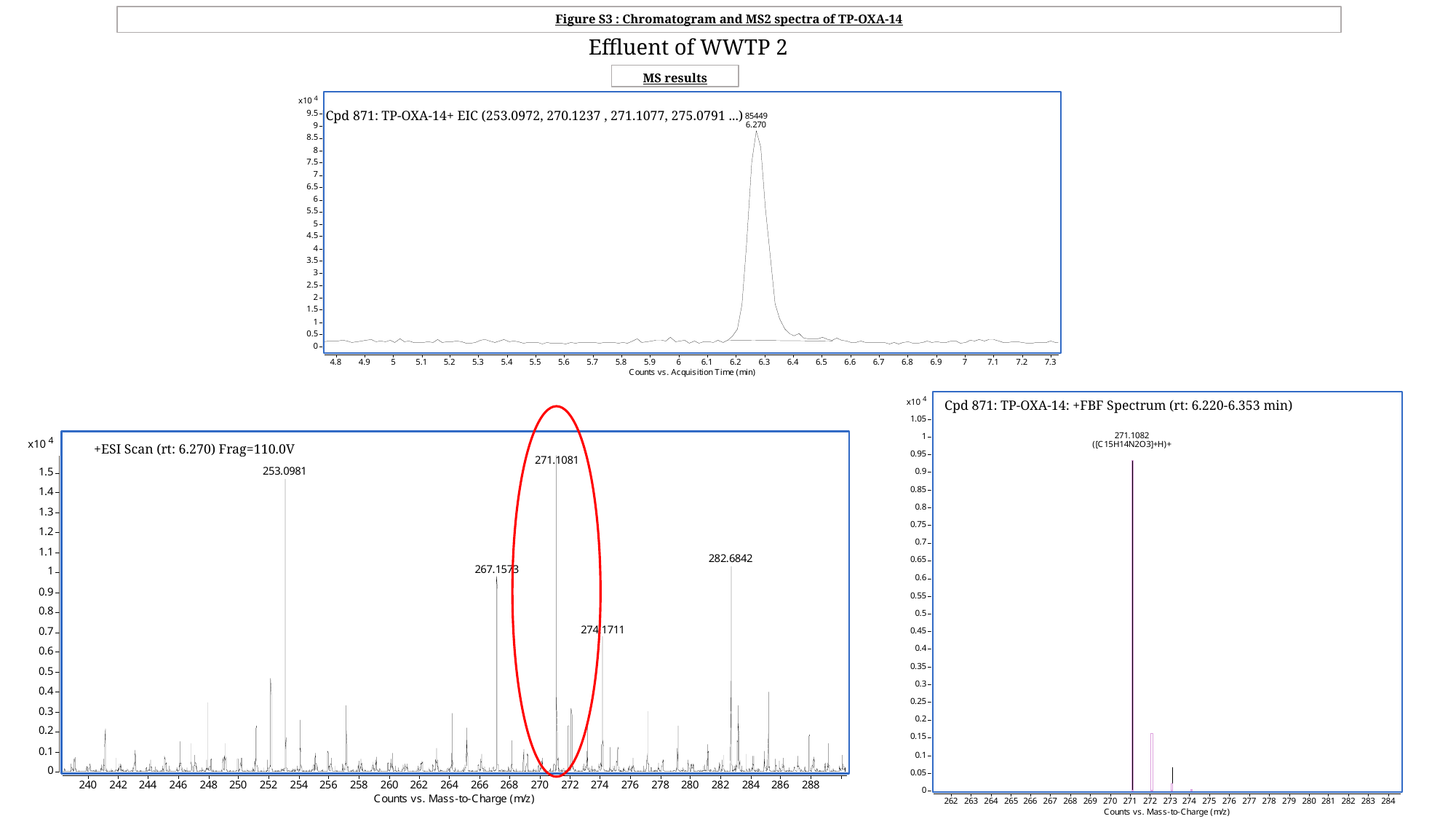

Figure S3 : Chromatogram and MS2 spectra of TP-OXA-14
Effluent of WWTP 2
MS results
Cpd 871: TP-OXA-14+ EIC (253.0972, 270.1237 , 271.1077, 275.0791 ...)
Cpd 871: TP-OXA-14: +FBF Spectrum (rt: 6.220-6.353 min)
+ESI Scan (rt: 6.270) Frag=110.0V

## Slide 11
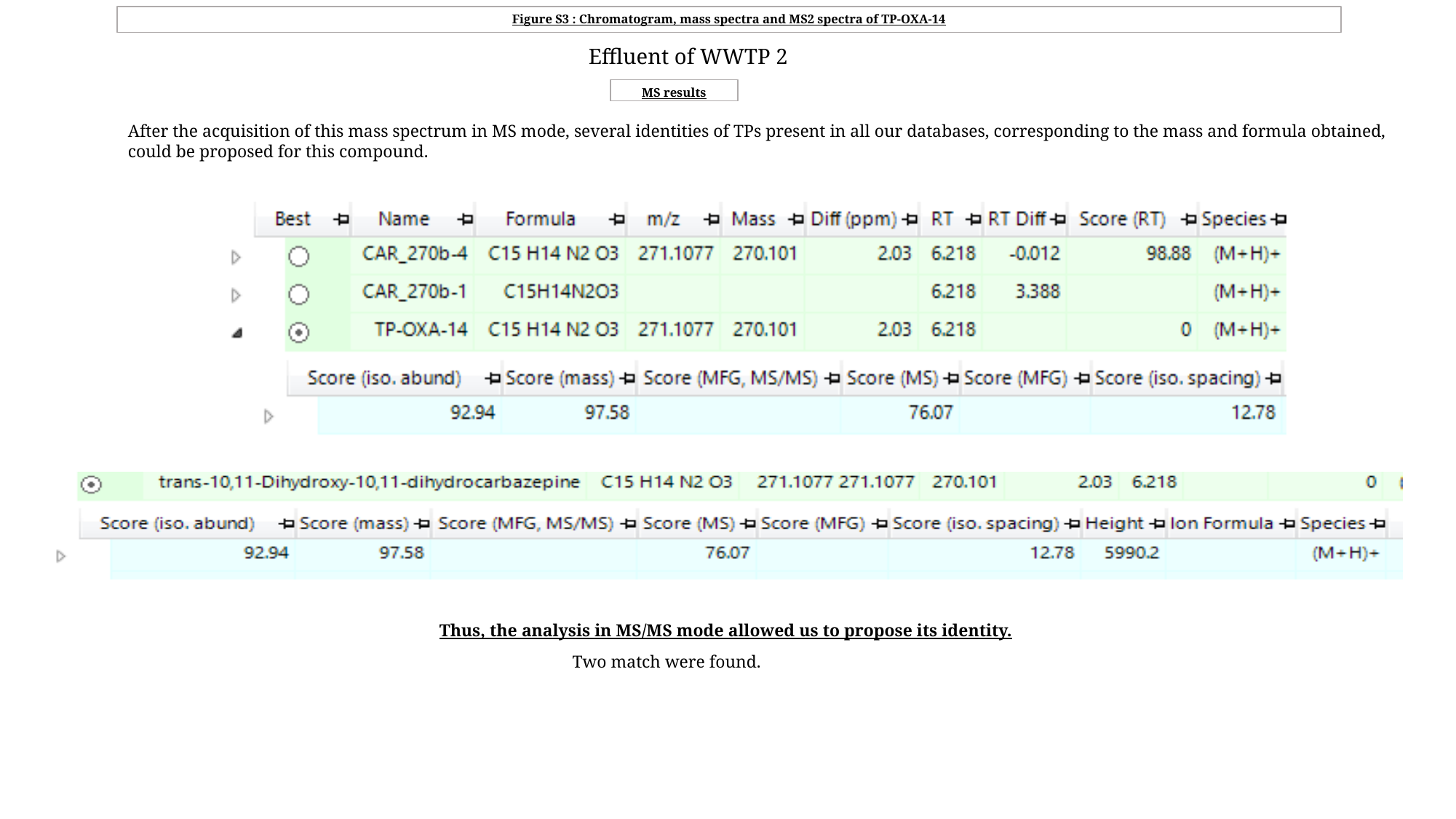

Figure S3 : Chromatogram, mass spectra and MS2 spectra of TP-OXA-14
Effluent of WWTP 2
MS results
After the acquisition of this mass spectrum in MS mode, several identities of TPs present in all our databases, corresponding to the mass and formula obtained, could be proposed for this compound.
Thus, the analysis in MS/MS mode allowed us to propose its identity.
Two match were found.

## Slide 12
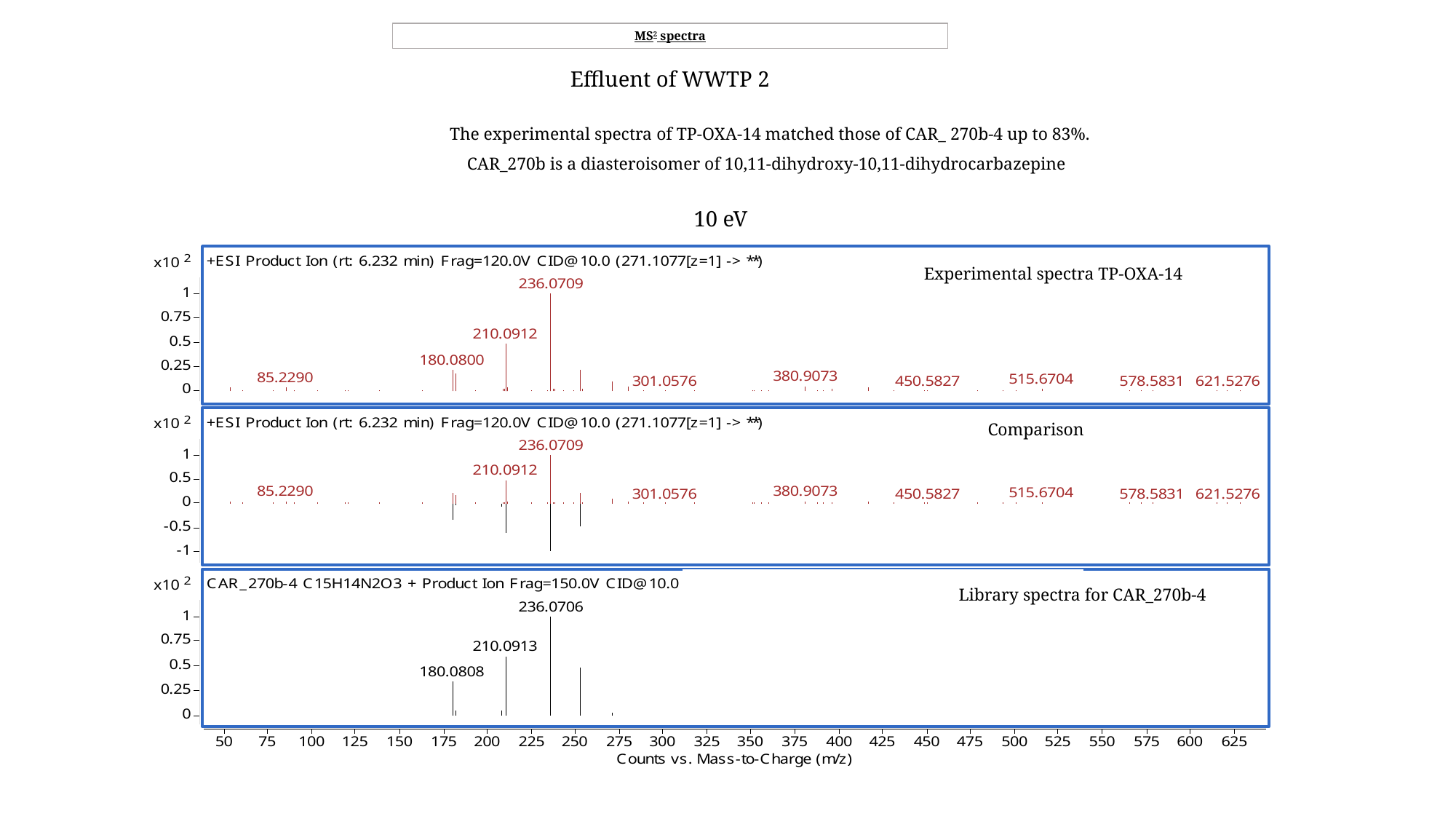

MS2 spectra
Effluent of WWTP 2
The experimental spectra of TP-OXA-14 matched those of CAR_ 270b-4 up to 83%.
CAR_270b is a diasteroisomer of 10,11-dihydroxy-10,11-dihydrocarbazepine
10 eV
Experimental spectra TP-OXA-14
Comparison
Library spectra for CAR_270b-4

## Slide 13
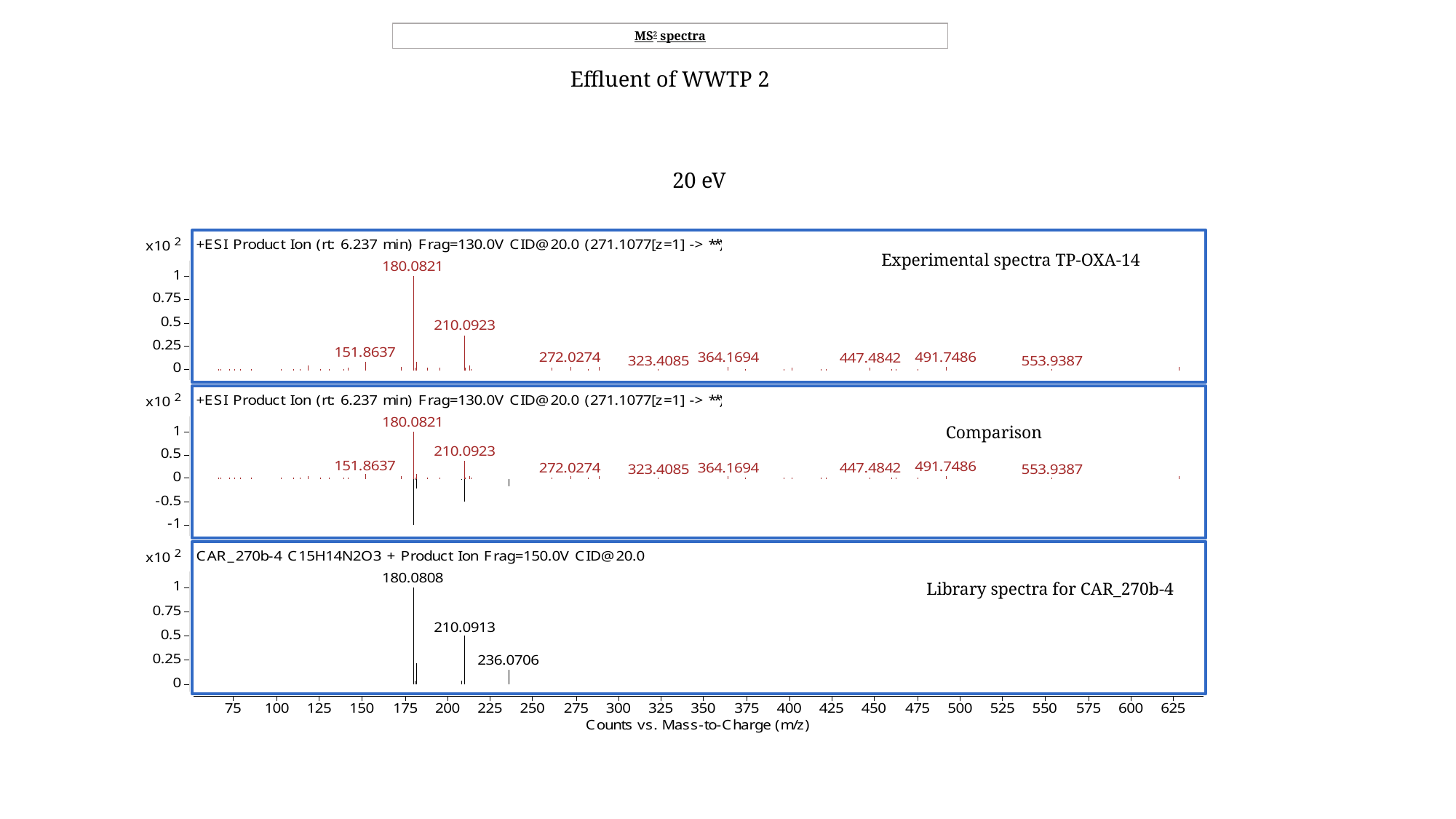

MS2 spectra
Effluent of WWTP 2
20 eV
Comparison
Experimental spectra TP-OXA-14
Library spectra for CAR_270b-4

## Slide 14
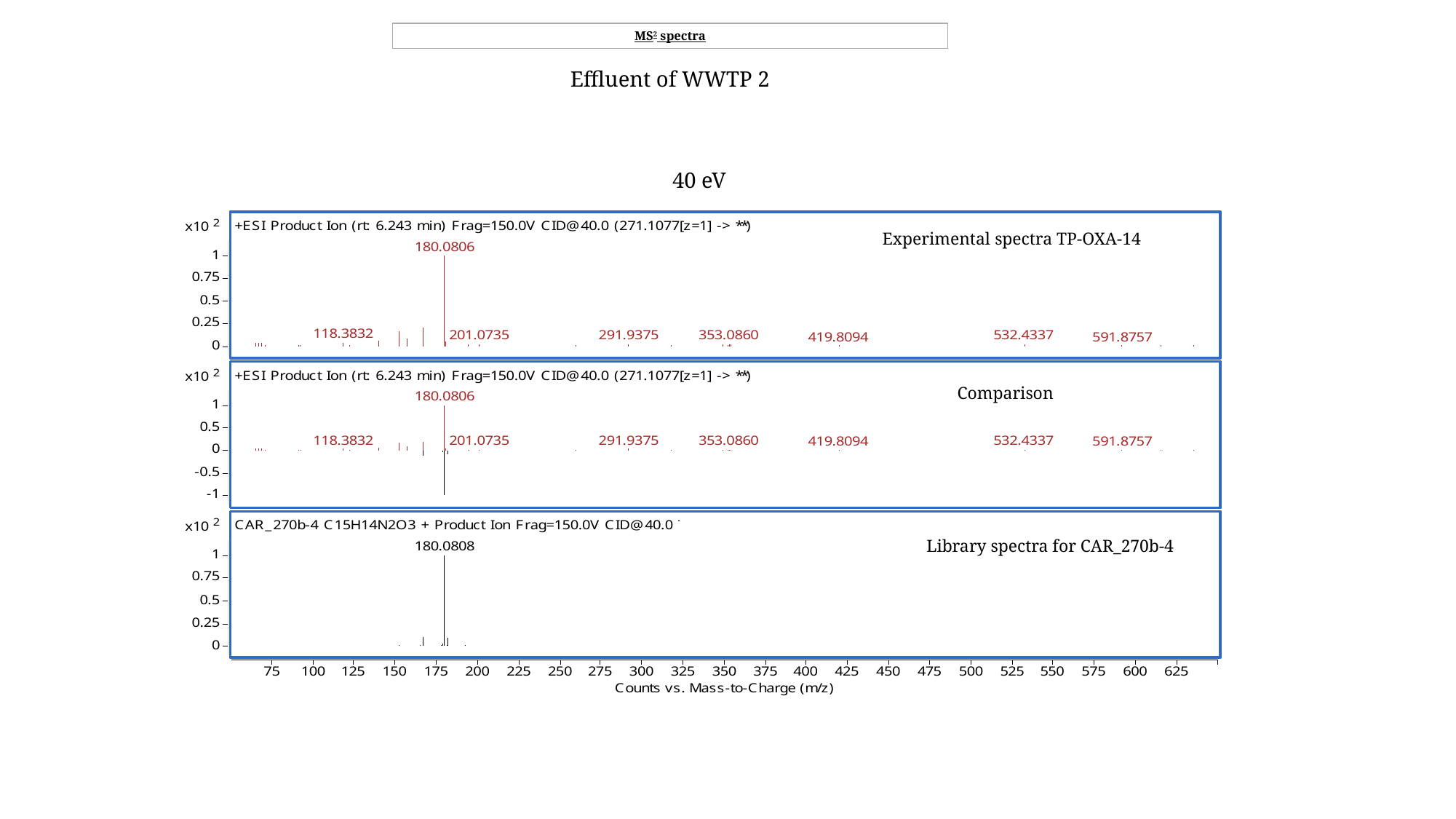

MS2 spectra
Effluent of WWTP 2
40 eV
Comparison
Experimental spectra TP-OXA-14
Library spectra for CAR_270b-4

## Slide 15
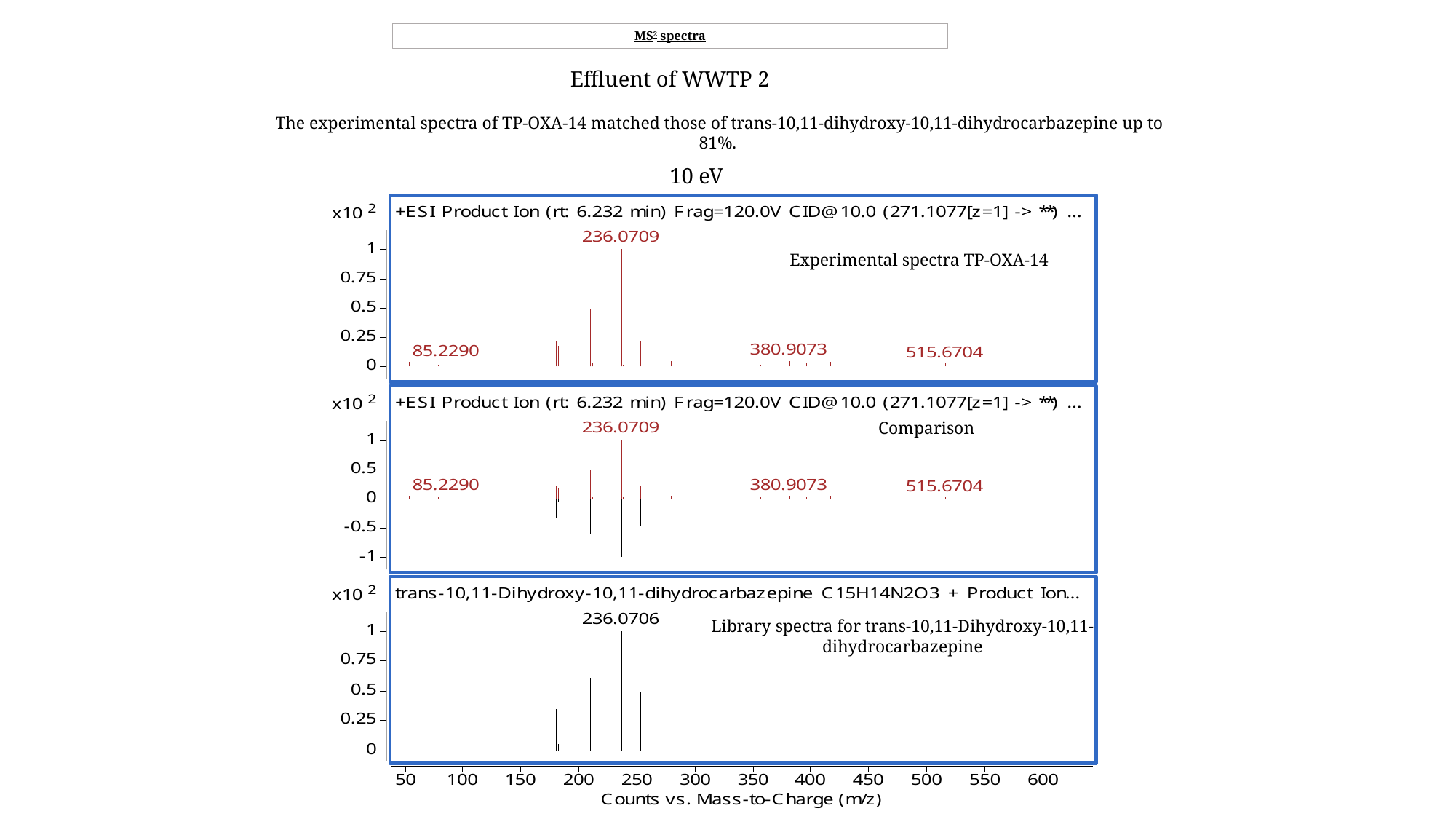

MS2 spectra
Effluent of WWTP 2
The experimental spectra of TP-OXA-14 matched those of trans-10,11-dihydroxy-10,11-dihydrocarbazepine up to 81%.
10 eV
Experimental spectra TP-OXA-14
Comparison
Library spectra for trans-10,11-Dihydroxy-10,11-dihydrocarbazepine

## Slide 16
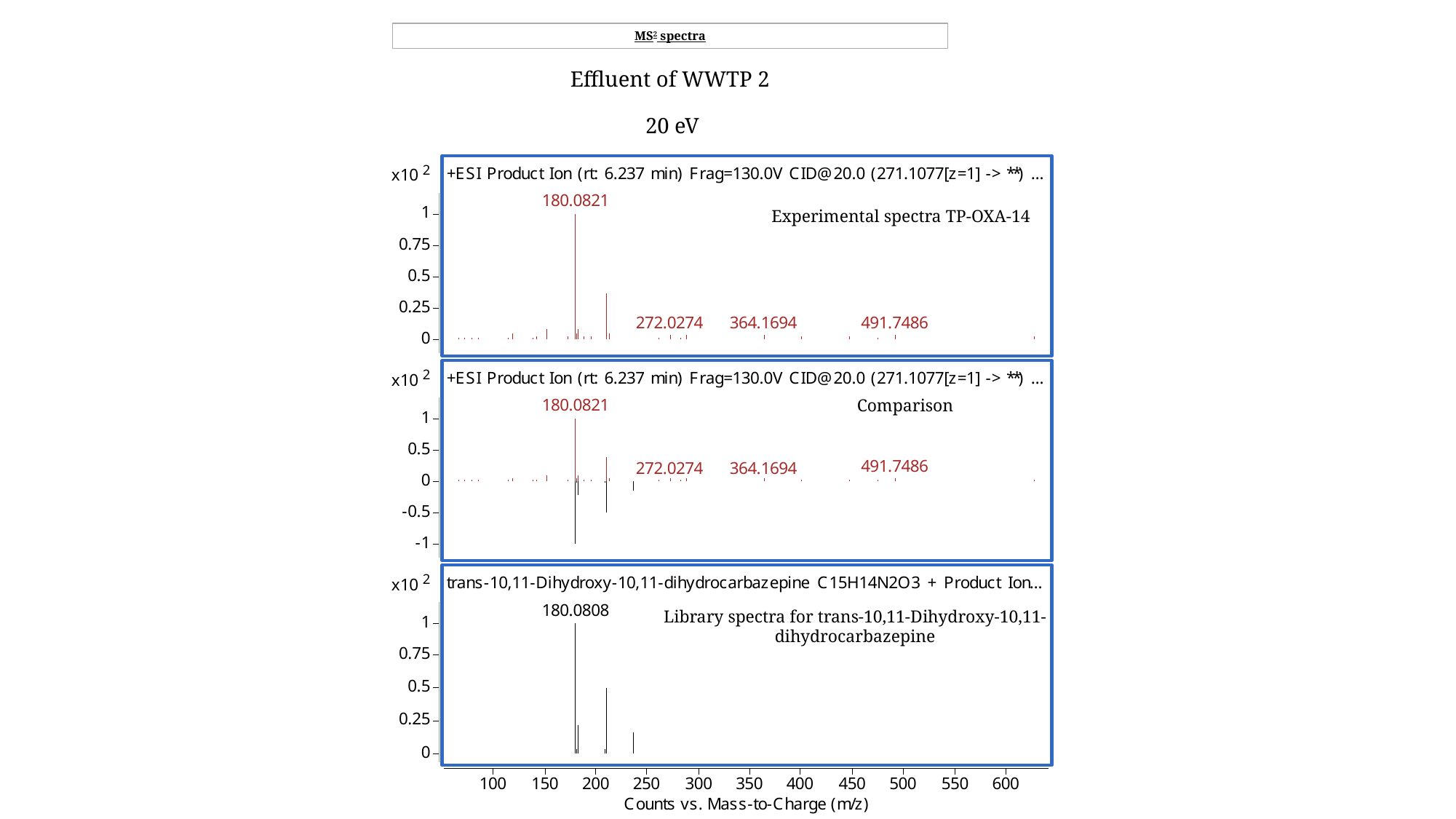

MS2 spectra
Effluent of WWTP 2
20 eV
Experimental spectra TP-OXA-14
Comparison
Library spectra for trans-10,11-Dihydroxy-10,11-dihydrocarbazepine

## Slide 17
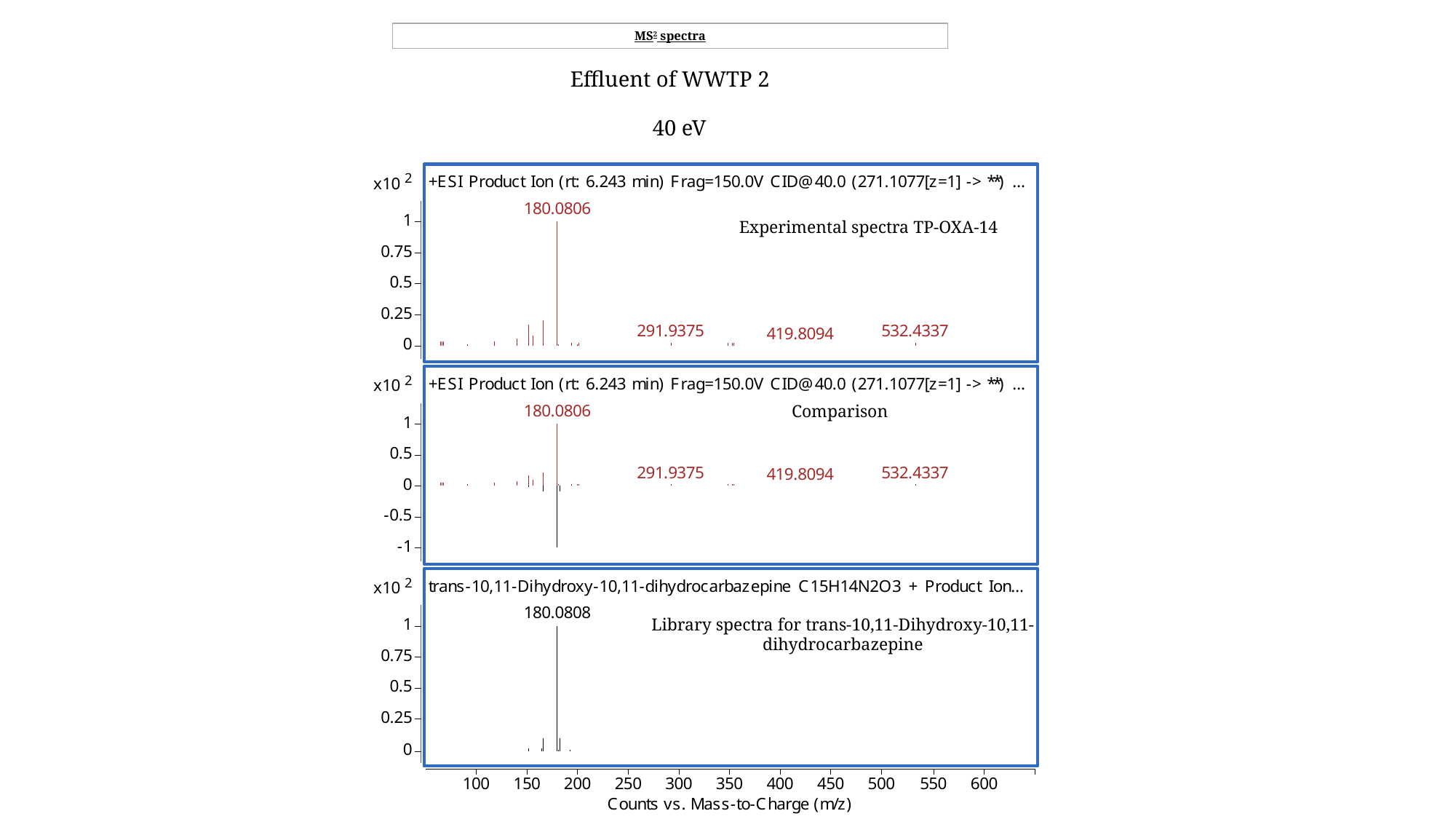

MS2 spectra
Effluent of WWTP 2
40 eV
Experimental spectra TP-OXA-14
Comparison
Library spectra for trans-10,11-Dihydroxy-10,11-dihydrocarbazepine

## Slide 18
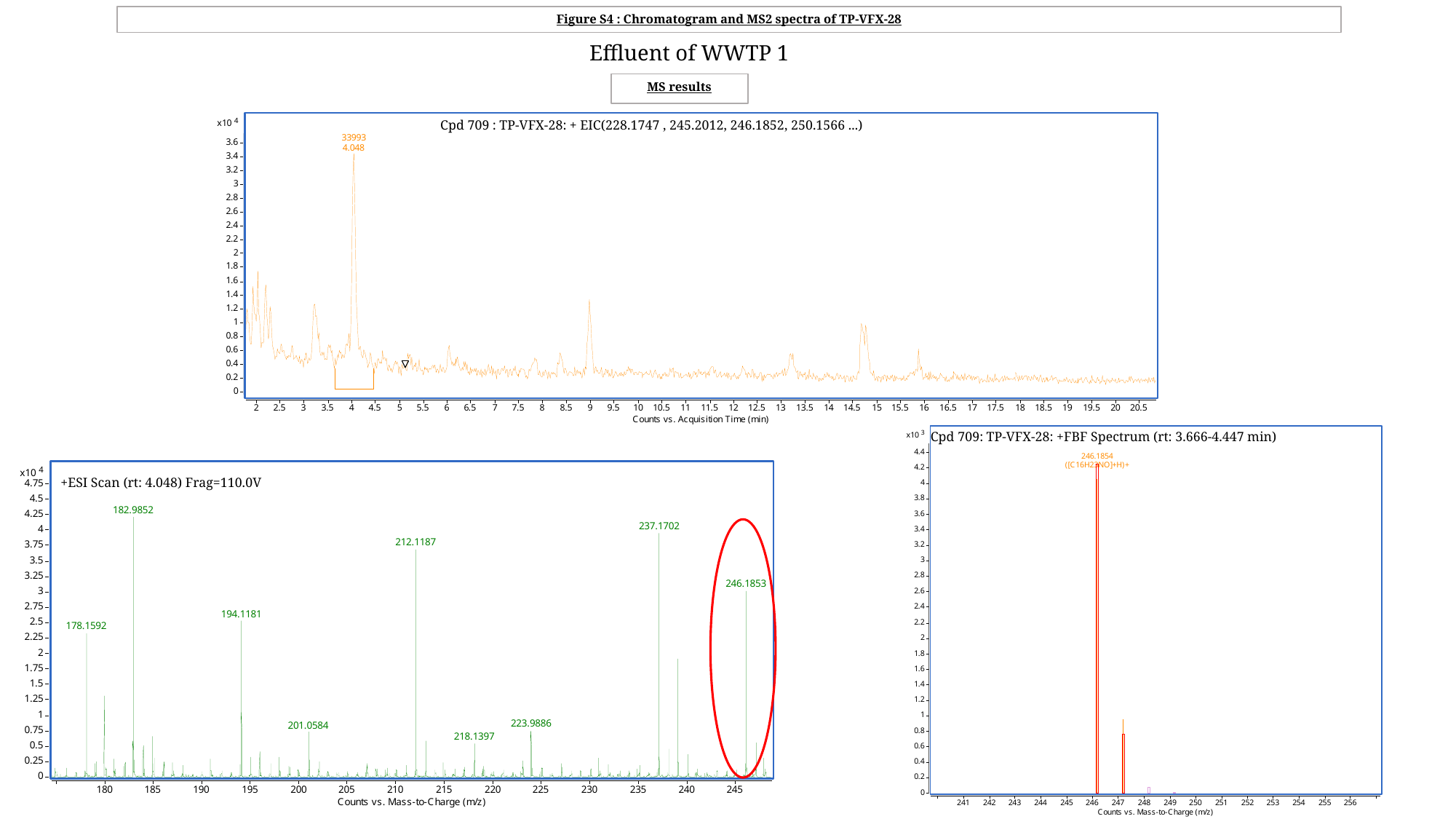

Figure S4 : Chromatogram and MS2 spectra of TP-VFX-28
Effluent of WWTP 1
MS results
Cpd 709 : TP-VFX-28: + EIC(228.1747 , 245.2012, 246.1852, 250.1566 ...)
Cpd 709: TP-VFX-28: +FBF Spectrum (rt: 3.666-4.447 min)
+ESI Scan (rt: 4.048) Frag=110.0V

## Slide 19
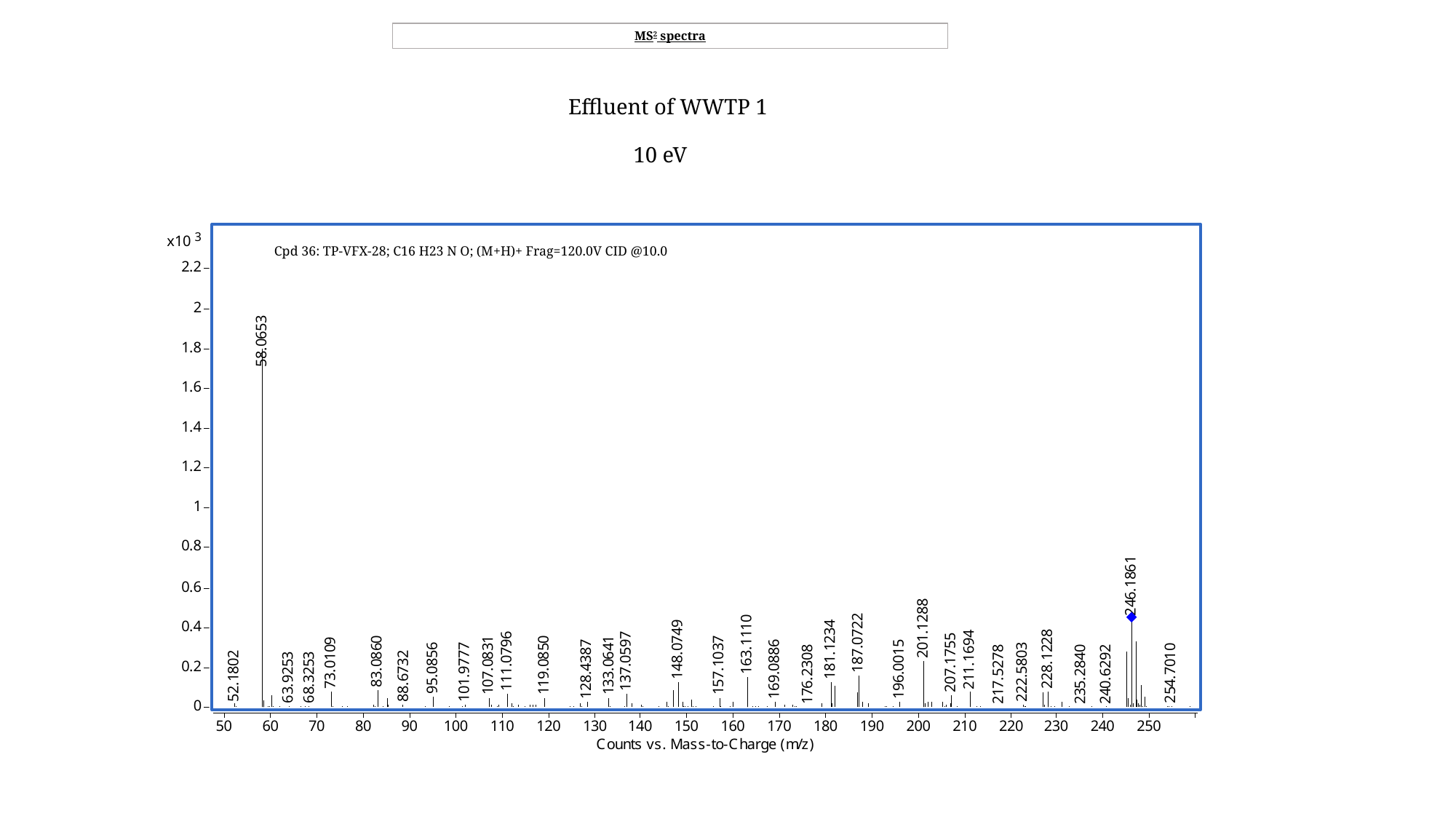

MS2 spectra
Effluent of WWTP 1
10 eV
Cpd 36: TP-VFX-28; C16 H23 N O; (M+H)+ Frag=120.0V CID @10.0

## Slide 20
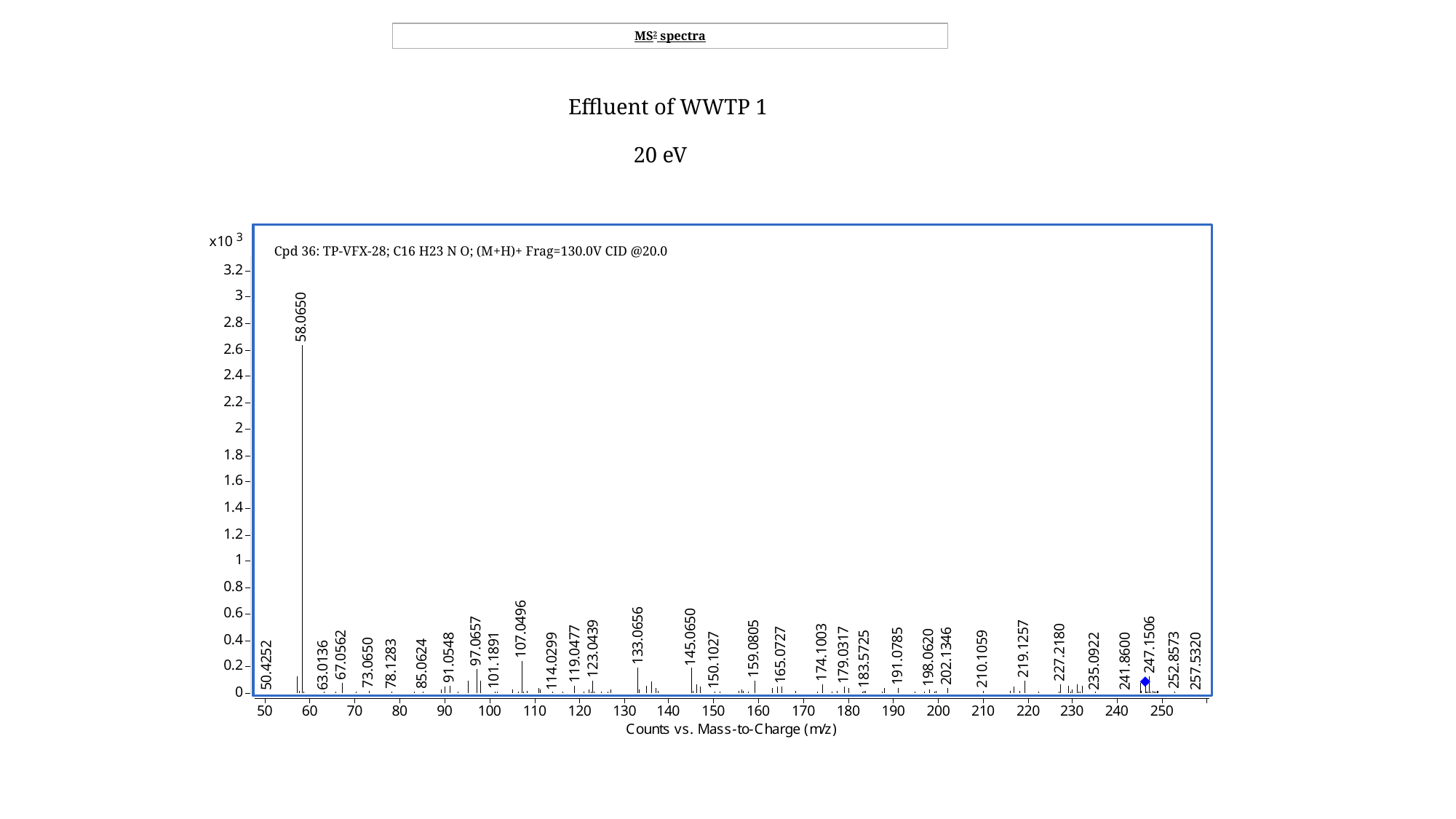

MS2 spectra
Effluent of WWTP 1
20 eV
Cpd 36: TP-VFX-28; C16 H23 N O; (M+H)+ Frag=130.0V CID @20.0

## Slide 21
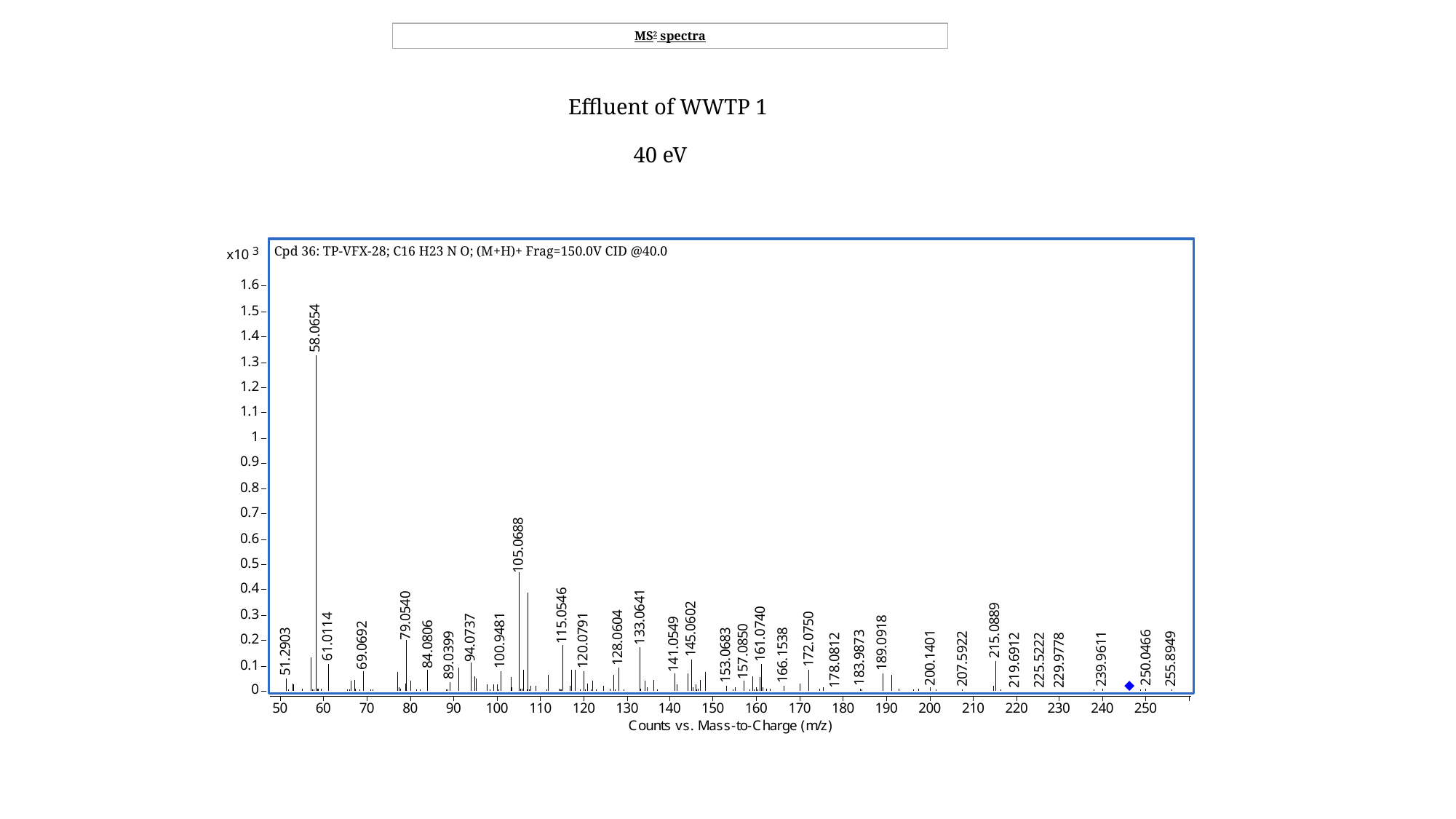

MS2 spectra
Effluent of WWTP 1
40 eV
Cpd 36: TP-VFX-28; C16 H23 N O; (M+H)+ Frag=150.0V CID @40.0

## Slide 22
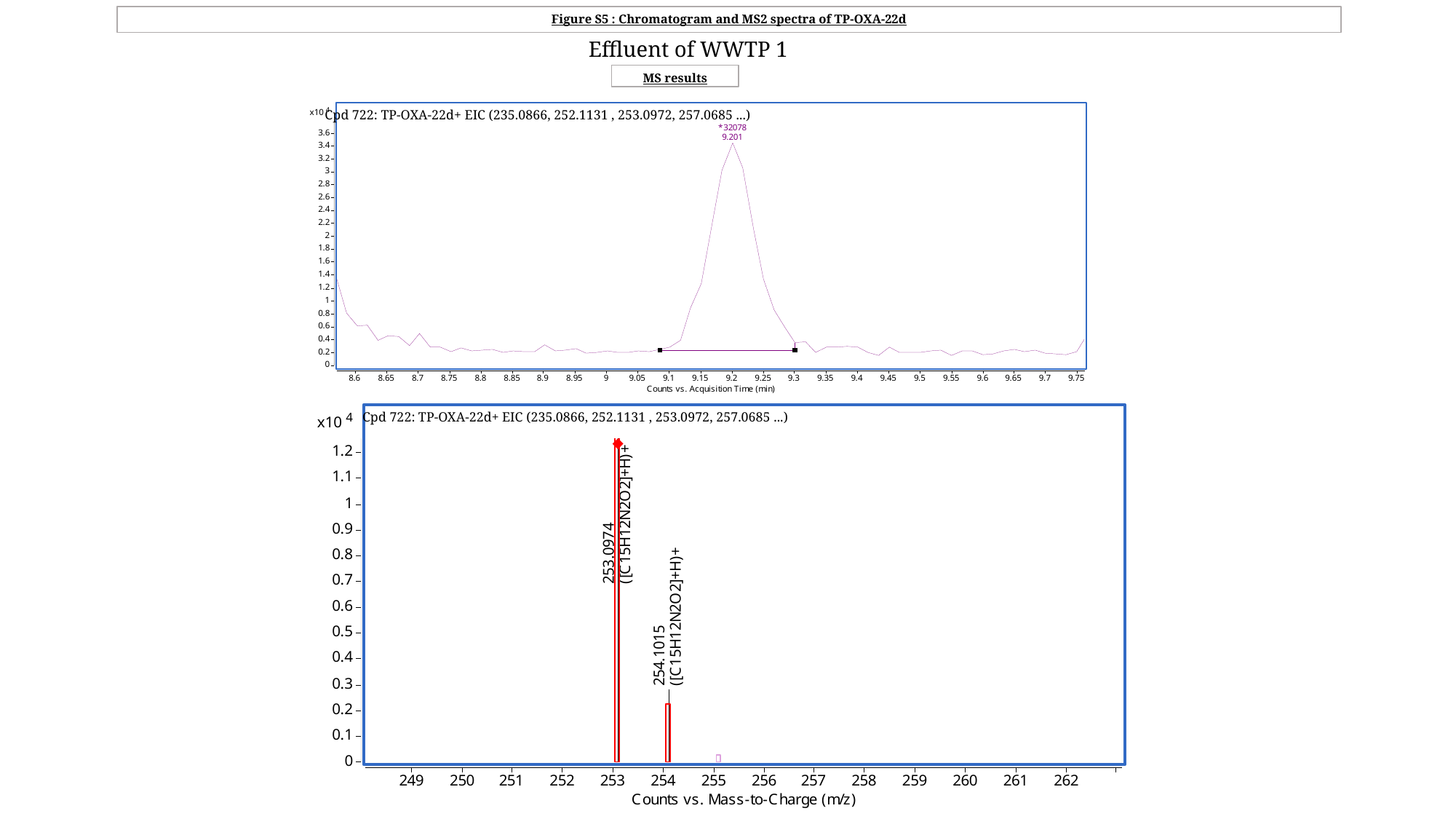

Figure S5 : Chromatogram and MS2 spectra of TP-OXA-22d
Effluent of WWTP 1
MS results
Cpd 722: TP-OXA-22d+ EIC (235.0866, 252.1131 , 253.0972, 257.0685 ...)
Cpd 722: TP-OXA-22d+ EIC (235.0866, 252.1131 , 253.0972, 257.0685 ...)

## Slide 23
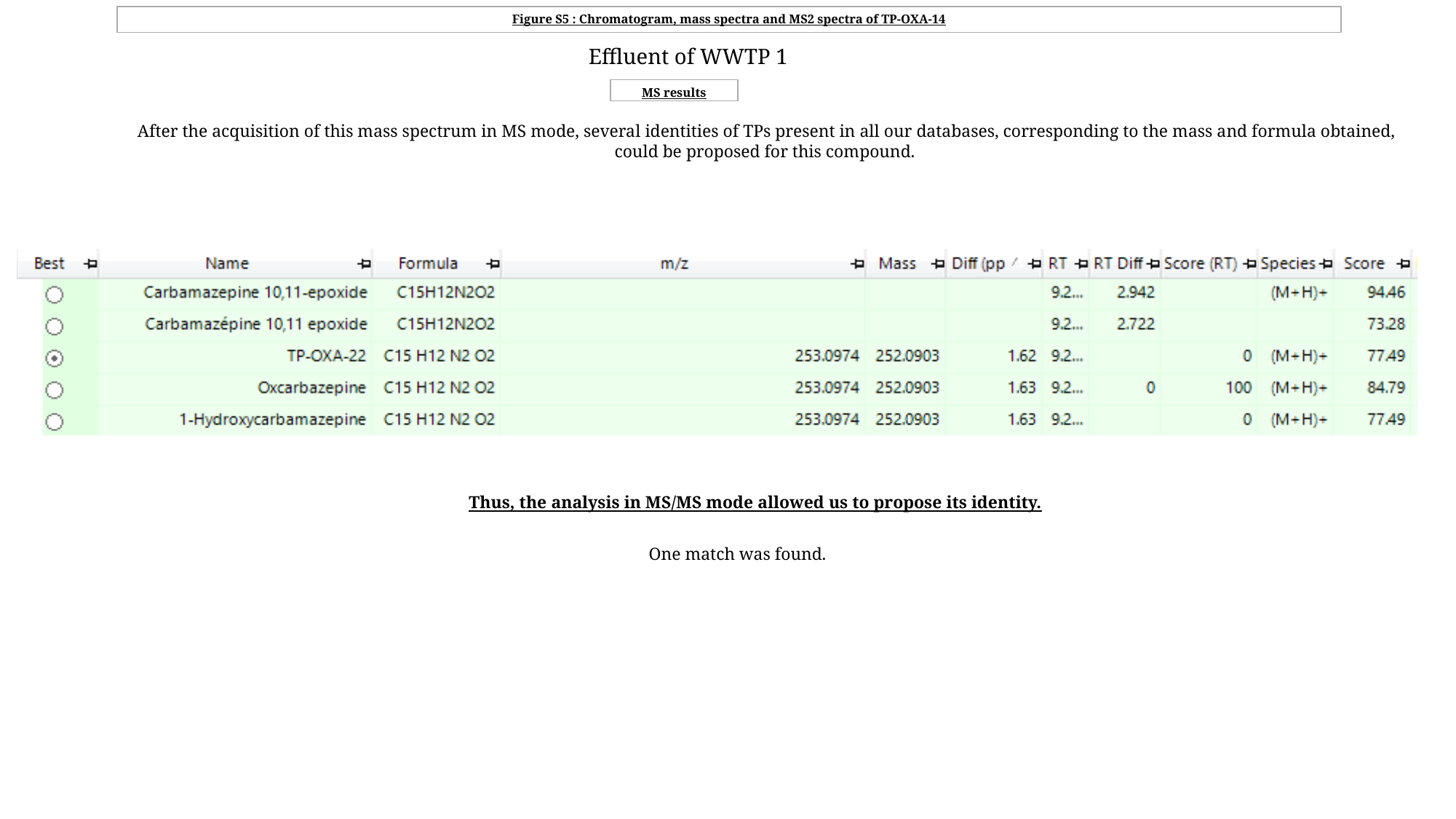

Figure S5 : Chromatogram, mass spectra and MS2 spectra of TP-OXA-14
Effluent of WWTP 1
MS results
After the acquisition of this mass spectrum in MS mode, several identities of TPs present in all our databases, corresponding to the mass and formula obtained, could be proposed for this compound.
Thus, the analysis in MS/MS mode allowed us to propose its identity.
One match was found.

## Slide 24
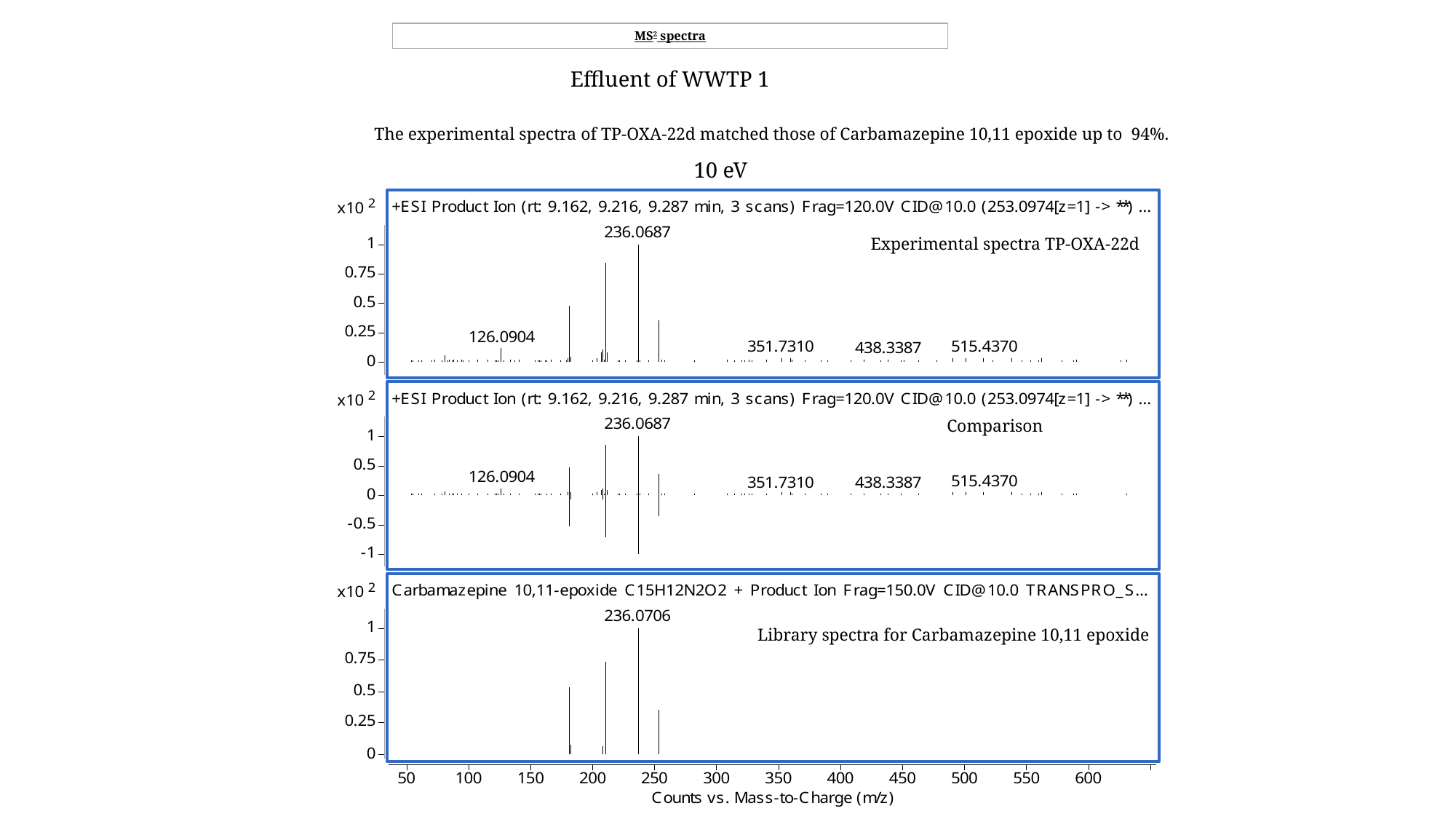

MS2 spectra
Effluent of WWTP 1
The experimental spectra of TP-OXA-22d matched those of Carbamazepine 10,11 epoxide up to 94%.
10 eV
Experimental spectra TP-OXA-22d
Comparison
Library spectra for Carbamazepine 10,11 epoxide

## Slide 25
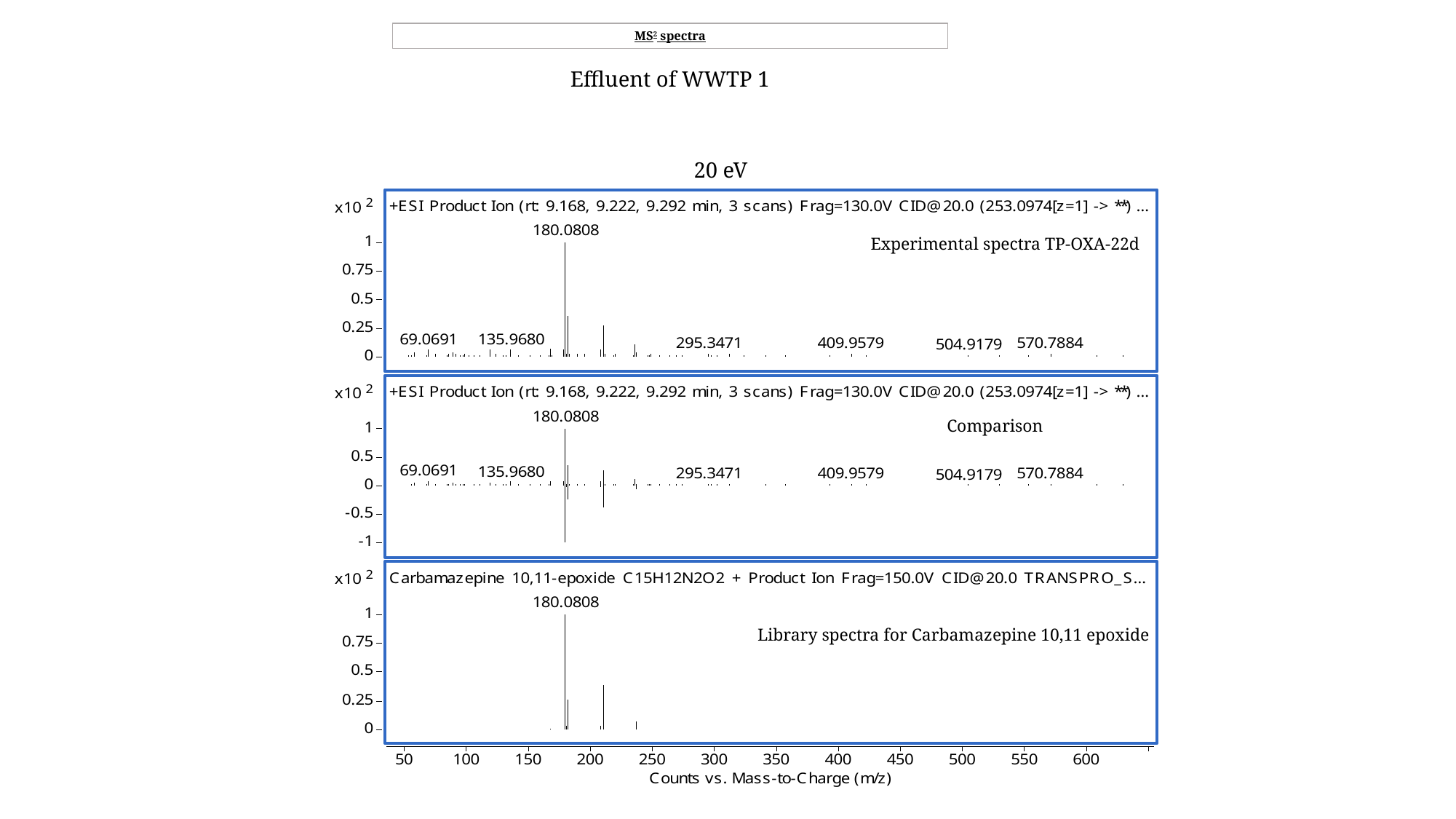

MS2 spectra
Effluent of WWTP 1
20 eV
Experimental spectra TP-OXA-22d
Comparison
Library spectra for Carbamazepine 10,11 epoxide

## Slide 26
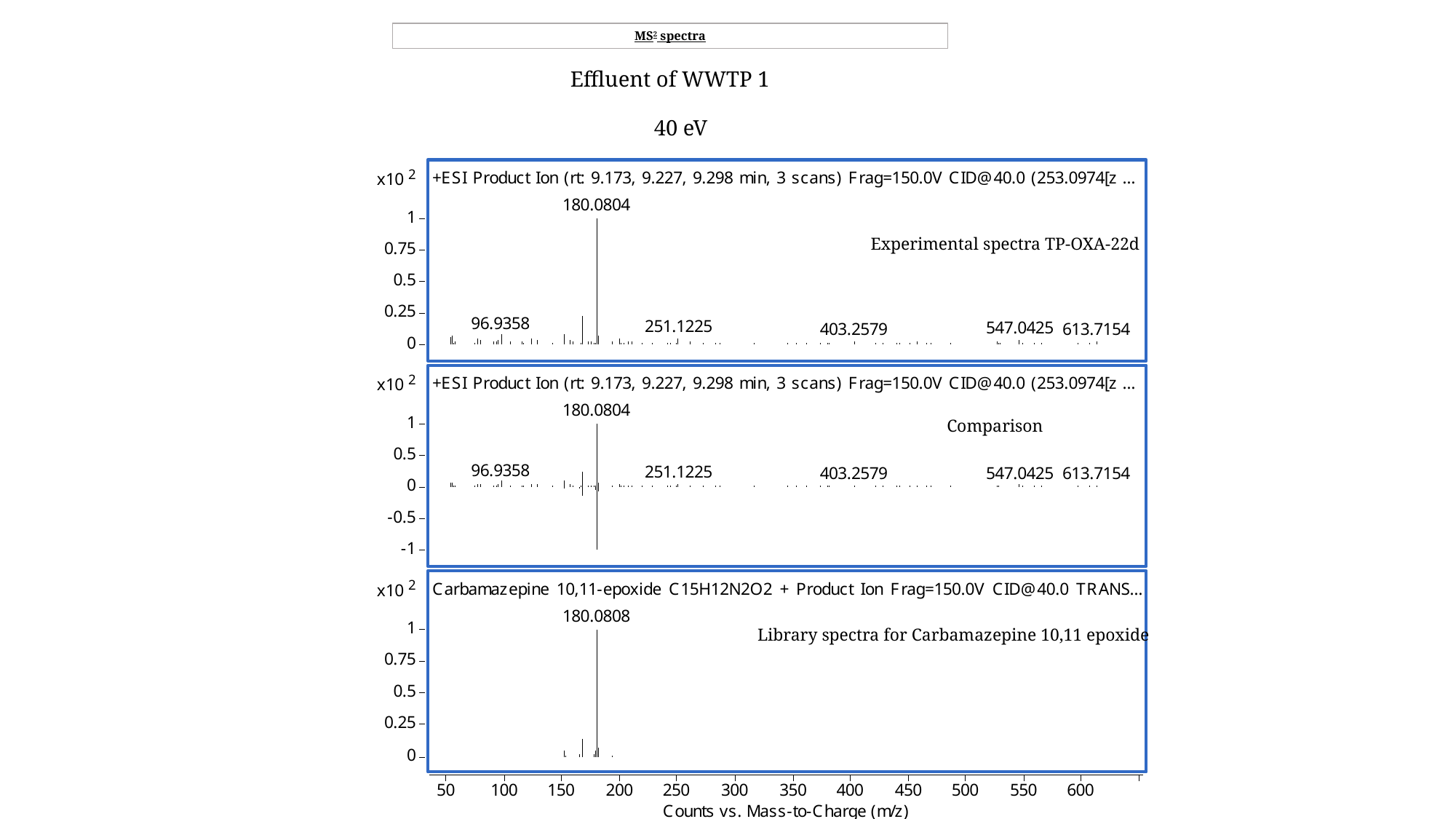

MS2 spectra
Effluent of WWTP 1
40 eV
Experimental spectra TP-OXA-22d
Comparison
Library spectra for Carbamazepine 10,11 epoxide

## Slide 27
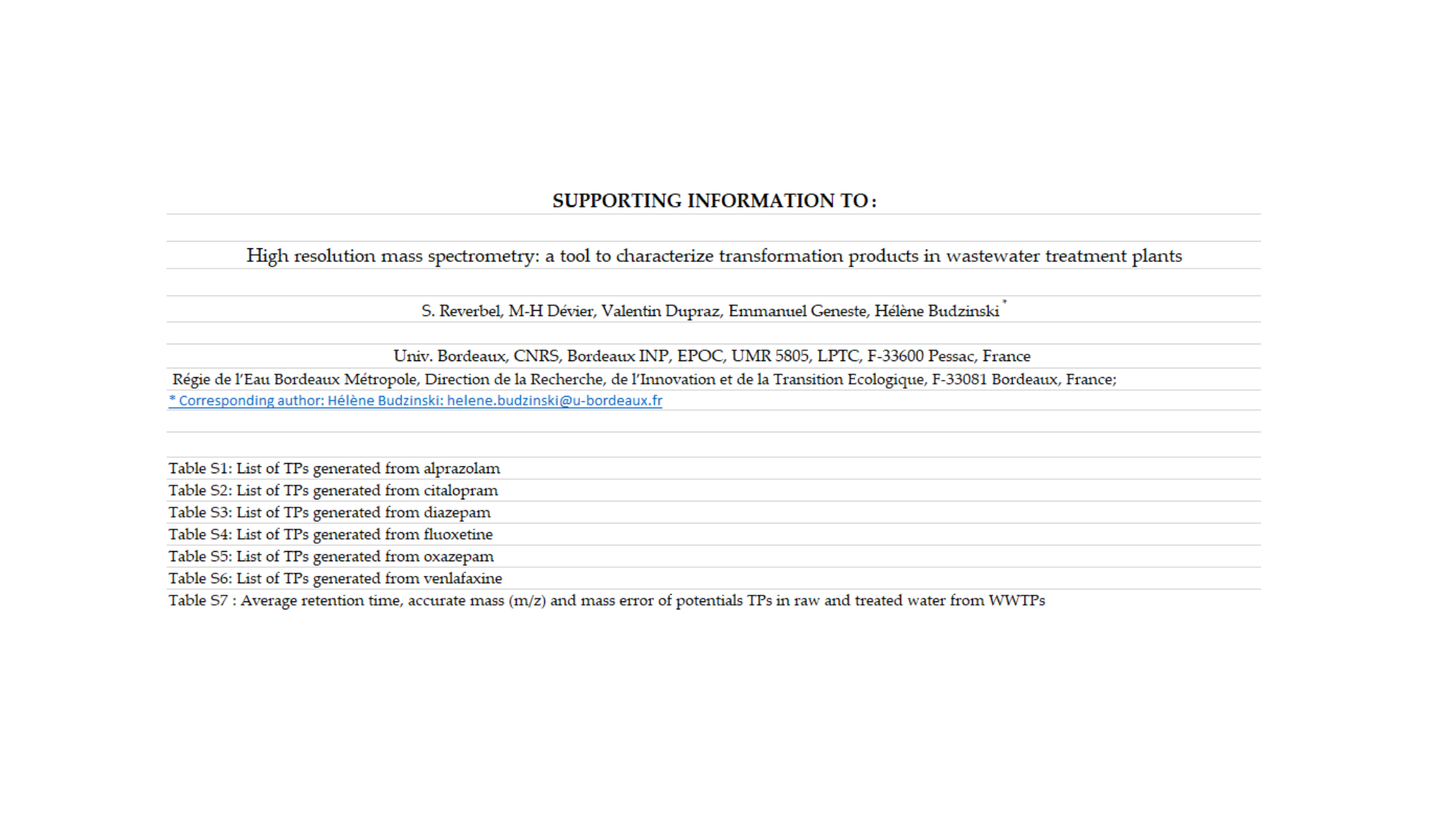

## Slide 28
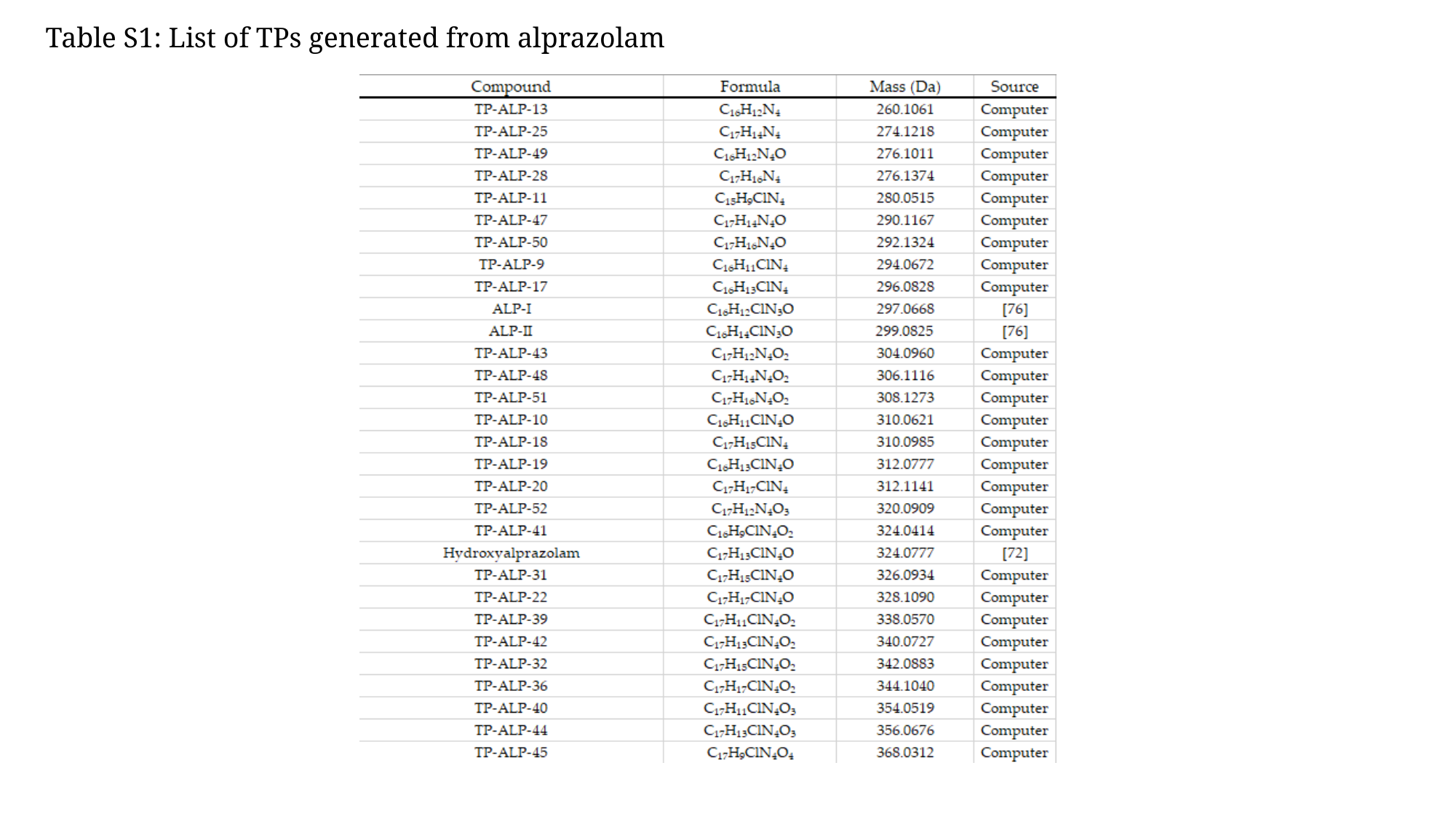

Table S1: List of TPs generated from alprazolam

## Slide 29
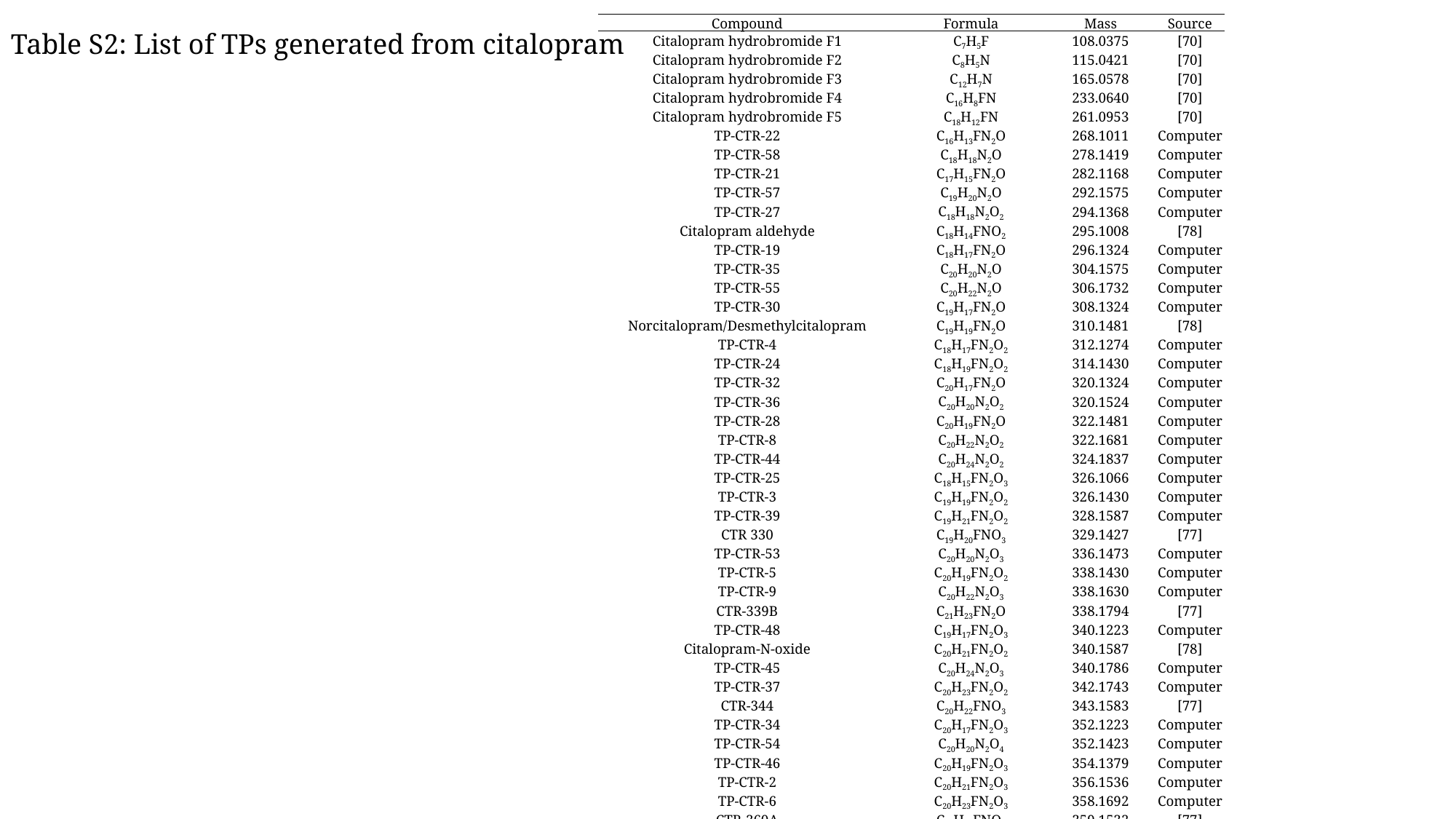

| Compound | Formula | Mass | Source |
| --- | --- | --- | --- |
| Citalopram hydrobromide F1 | C7H5F | 108.0375 | [70] |
| Citalopram hydrobromide F2 | C8H5N | 115.0421 | [70] |
| Citalopram hydrobromide F3 | C12H7N | 165.0578 | [70] |
| Citalopram hydrobromide F4 | C16H8FN | 233.0640 | [70] |
| Citalopram hydrobromide F5 | C18H12FN | 261.0953 | [70] |
| TP-CTR-22 | C16H13FN2O | 268.1011 | Computer |
| TP-CTR-58 | C18H18N2O | 278.1419 | Computer |
| TP-CTR-21 | C17H15FN2O | 282.1168 | Computer |
| TP-CTR-57 | C19H20N2O | 292.1575 | Computer |
| TP-CTR-27 | C18H18N2O2 | 294.1368 | Computer |
| Citalopram aldehyde | C18H14FNO2 | 295.1008 | [78] |
| TP-CTR-19 | C18H17FN2O | 296.1324 | Computer |
| TP-CTR-35 | C20H20N2O | 304.1575 | Computer |
| TP-CTR-55 | C20H22N2O | 306.1732 | Computer |
| TP-CTR-30 | C19H17FN2O | 308.1324 | Computer |
| Norcitalopram/Desmethylcitalopram | C19H19FN2O | 310.1481 | [78] |
| TP-CTR-4 | C18H17FN2O2 | 312.1274 | Computer |
| TP-CTR-24 | C18H19FN2O2 | 314.1430 | Computer |
| TP-CTR-32 | C20H17FN2O | 320.1324 | Computer |
| TP-CTR-36 | C20H20N2O2 | 320.1524 | Computer |
| TP-CTR-28 | C20H19FN2O | 322.1481 | Computer |
| TP-CTR-8 | C20H22N2O2 | 322.1681 | Computer |
| TP-CTR-44 | C20H24N2O2 | 324.1837 | Computer |
| TP-CTR-25 | C18H15FN2O3 | 326.1066 | Computer |
| TP-CTR-3 | C19H19FN2O2 | 326.1430 | Computer |
| TP-CTR-39 | C19H21FN2O2 | 328.1587 | Computer |
| CTR 330 | C19H20FNO3 | 329.1427 | [77] |
| TP-CTR-53 | C20H20N2O3 | 336.1473 | Computer |
| TP-CTR-5 | C20H19FN2O2 | 338.1430 | Computer |
| TP-CTR-9 | C20H22N2O3 | 338.1630 | Computer |
| CTR-339B | C21H23FN2O | 338.1794 | [77] |
| TP-CTR-48 | C19H17FN2O3 | 340.1223 | Computer |
| Citalopram-N-oxide | C20H21FN2O2 | 340.1587 | [78] |
| TP-CTR-45 | C20H24N2O3 | 340.1786 | Computer |
| TP-CTR-37 | C20H23FN2O2 | 342.1743 | Computer |
| CTR-344 | C20H22FNO3 | 343.1583 | [77] |
| TP-CTR-34 | C20H17FN2O3 | 352.1223 | Computer |
| TP-CTR-54 | C20H20N2O4 | 352.1423 | Computer |
| TP-CTR-46 | C20H19FN2O3 | 354.1379 | Computer |
| TP-CTR-2 | C20H21FN2O3 | 356.1536 | Computer |
| TP-CTR-6 | C20H23FN2O3 | 358.1692 | Computer |
| CTR-360A | C20H22FNO4 | 359.1532 | [77] |
| TP-CTR-42 | C20H25FN2O3 | 360.1849 | Computer |
| TP-CTR-7 | C20H19FN2O4 | 370.1328 | Computer |
| TP-CTR-43 | C20H21FN2O4 | 372.1485 | Computer |
| TP-CTR-52 | C20H17FN2O5 | 384.1121 | Computer |
| Dimethylamine | C2H7N | 45.0578 | Computer |
Table S2: List of TPs generated from citalopram

## Slide 30
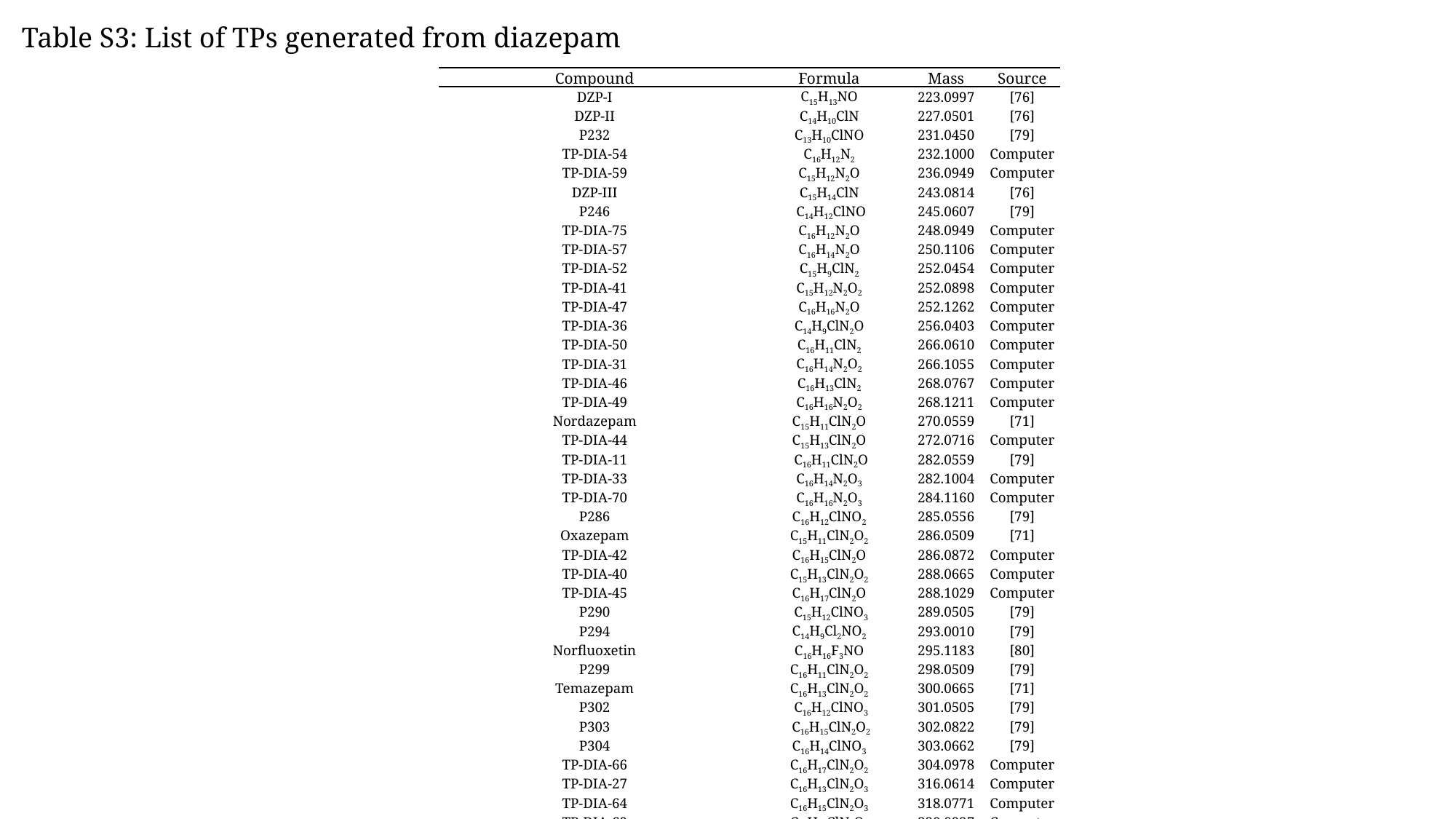

Table S3: List of TPs generated from diazepam
| Compound | Formula | Mass | Source |
| --- | --- | --- | --- |
| DZP-I | C15H13NO | 223.0997 | [76] |
| DZP-II | C14H10ClN | 227.0501 | [76] |
| P232 | C13H10ClNO | 231.0450 | [79] |
| TP-DIA-54 | C16H12N2 | 232.1000 | Computer |
| TP-DIA-59 | C15H12N2O | 236.0949 | Computer |
| DZP-III | C15H14ClN | 243.0814 | [76] |
| P246 | C14H12ClNO | 245.0607 | [79] |
| TP-DIA-75 | C16H12N2O | 248.0949 | Computer |
| TP-DIA-57 | C16H14N2O | 250.1106 | Computer |
| TP-DIA-52 | C15H9ClN2 | 252.0454 | Computer |
| TP-DIA-41 | C15H12N2O2 | 252.0898 | Computer |
| TP-DIA-47 | C16H16N2O | 252.1262 | Computer |
| TP-DIA-36 | C14H9ClN2O | 256.0403 | Computer |
| TP-DIA-50 | C16H11ClN2 | 266.0610 | Computer |
| TP-DIA-31 | C16H14N2O2 | 266.1055 | Computer |
| TP-DIA-46 | C16H13ClN2 | 268.0767 | Computer |
| TP-DIA-49 | C16H16N2O2 | 268.1211 | Computer |
| Nordazepam | C15H11ClN2O | 270.0559 | [71] |
| TP-DIA-44 | C15H13ClN2O | 272.0716 | Computer |
| TP-DIA-11 | C16H11ClN2O | 282.0559 | [79] |
| TP-DIA-33 | C16H14N2O3 | 282.1004 | Computer |
| TP-DIA-70 | C16H16N2O3 | 284.1160 | Computer |
| P286 | C16H12ClNO2 | 285.0556 | [79] |
| Oxazepam | C15H11ClN2O2 | 286.0509 | [71] |
| TP-DIA-42 | C16H15ClN2O | 286.0872 | Computer |
| TP-DIA-40 | C15H13ClN2O2 | 288.0665 | Computer |
| TP-DIA-45 | C16H17ClN2O | 288.1029 | Computer |
| P290 | C15H12ClNO3 | 289.0505 | [79] |
| P294 | C14H9Cl2NO2 | 293.0010 | [79] |
| Norfluoxetin | C16H16F3NO | 295.1183 | [80] |
| P299 | C16H11ClN2O2 | 298.0509 | [79] |
| Temazepam | C16H13ClN2O2 | 300.0665 | [71] |
| P302 | C16H12ClNO3 | 301.0505 | [79] |
| P303 | C16H15ClN2O2 | 302.0822 | [79] |
| P304 | C16H14ClNO3 | 303.0662 | [79] |
| TP-DIA-66 | C16H17ClN2O2 | 304.0978 | Computer |
| TP-DIA-27 | C16H13ClN2O3 | 316.0614 | Computer |
| TP-DIA-64 | C16H15ClN2O3 | 318.0771 | Computer |
| TP-DIA-69 | C16H17ClN2O3 | 320.0927 | Computer |
| P599 | C32H24Cl2N4O4 | 598.1174 | [79] |

## Slide 31
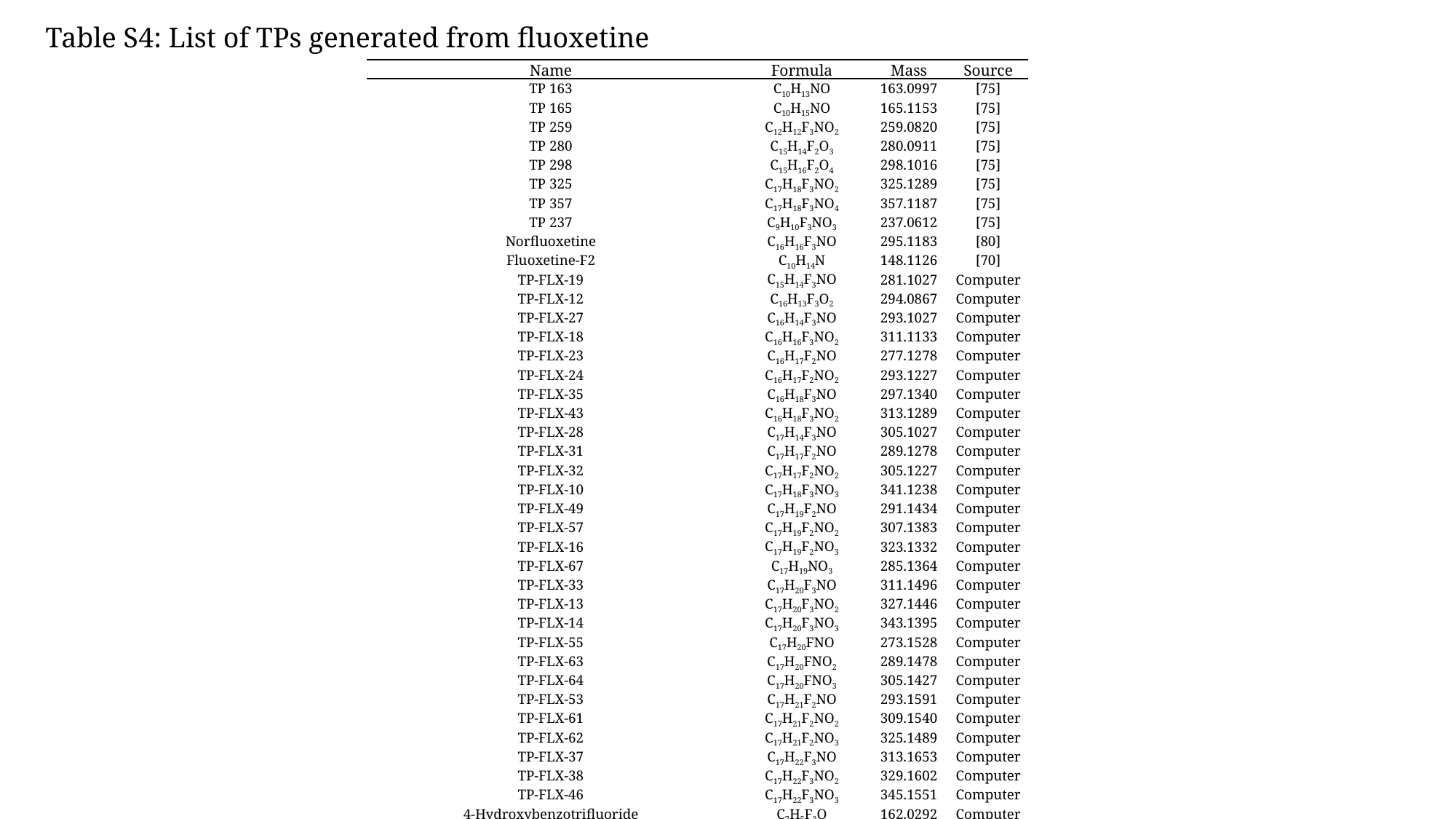

Table S4: List of TPs generated from fluoxetine
| Name | Formula | Mass | Source |
| --- | --- | --- | --- |
| TP 163 | C10H13NO | 163.0997 | [75] |
| TP 165 | C10H15NO | 165.1153 | [75] |
| TP 259 | C12H12F3NO2 | 259.0820 | [75] |
| TP 280 | C15H14F2O3 | 280.0911 | [75] |
| TP 298 | C15H16F2O4 | 298.1016 | [75] |
| TP 325 | C17H18F3NO2 | 325.1289 | [75] |
| TP 357 | C17H18F3NO4 | 357.1187 | [75] |
| TP 237 | C9H10F3NO3 | 237.0612 | [75] |
| Norfluoxetine | C16H16F3NO | 295.1183 | [80] |
| Fluoxetine-F2 | C10H14N | 148.1126 | [70] |
| TP-FLX-19 | C15H14F3NO | 281.1027 | Computer |
| TP-FLX-12 | C16H13F3O2 | 294.0867 | Computer |
| TP-FLX-27 | C16H14F3NO | 293.1027 | Computer |
| TP-FLX-18 | C16H16F3NO2 | 311.1133 | Computer |
| TP-FLX-23 | C16H17F2NO | 277.1278 | Computer |
| TP-FLX-24 | C16H17F2NO2 | 293.1227 | Computer |
| TP-FLX-35 | C16H18F3NO | 297.1340 | Computer |
| TP-FLX-43 | C16H18F3NO2 | 313.1289 | Computer |
| TP-FLX-28 | C17H14F3NO | 305.1027 | Computer |
| TP-FLX-31 | C17H17F2NO | 289.1278 | Computer |
| TP-FLX-32 | C17H17F2NO2 | 305.1227 | Computer |
| TP-FLX-10 | C17H18F3NO3 | 341.1238 | Computer |
| TP-FLX-49 | C17H19F2NO | 291.1434 | Computer |
| TP-FLX-57 | C17H19F2NO2 | 307.1383 | Computer |
| TP-FLX-16 | C17H19F2NO3 | 323.1332 | Computer |
| TP-FLX-67 | C17H19NO3 | 285.1364 | Computer |
| TP-FLX-33 | C17H20F3NO | 311.1496 | Computer |
| TP-FLX-13 | C17H20F3NO2 | 327.1446 | Computer |
| TP-FLX-14 | C17H20F3NO3 | 343.1395 | Computer |
| TP-FLX-55 | C17H20FNO | 273.1528 | Computer |
| TP-FLX-63 | C17H20FNO2 | 289.1478 | Computer |
| TP-FLX-64 | C17H20FNO3 | 305.1427 | Computer |
| TP-FLX-53 | C17H21F2NO | 293.1591 | Computer |
| TP-FLX-61 | C17H21F2NO2 | 309.1540 | Computer |
| TP-FLX-62 | C17H21F2NO3 | 325.1489 | Computer |
| TP-FLX-37 | C17H22F3NO | 313.1653 | Computer |
| TP-FLX-38 | C17H22F3NO2 | 329.1602 | Computer |
| TP-FLX-46 | C17H22F3NO3 | 345.1551 | Computer |
| 4-Hydroxybenzotrifluoride | C7H5F3O | 162.0292 | Computer |
| Methylamine | CH5N | 31.04219 | Computer |

## Slide 32
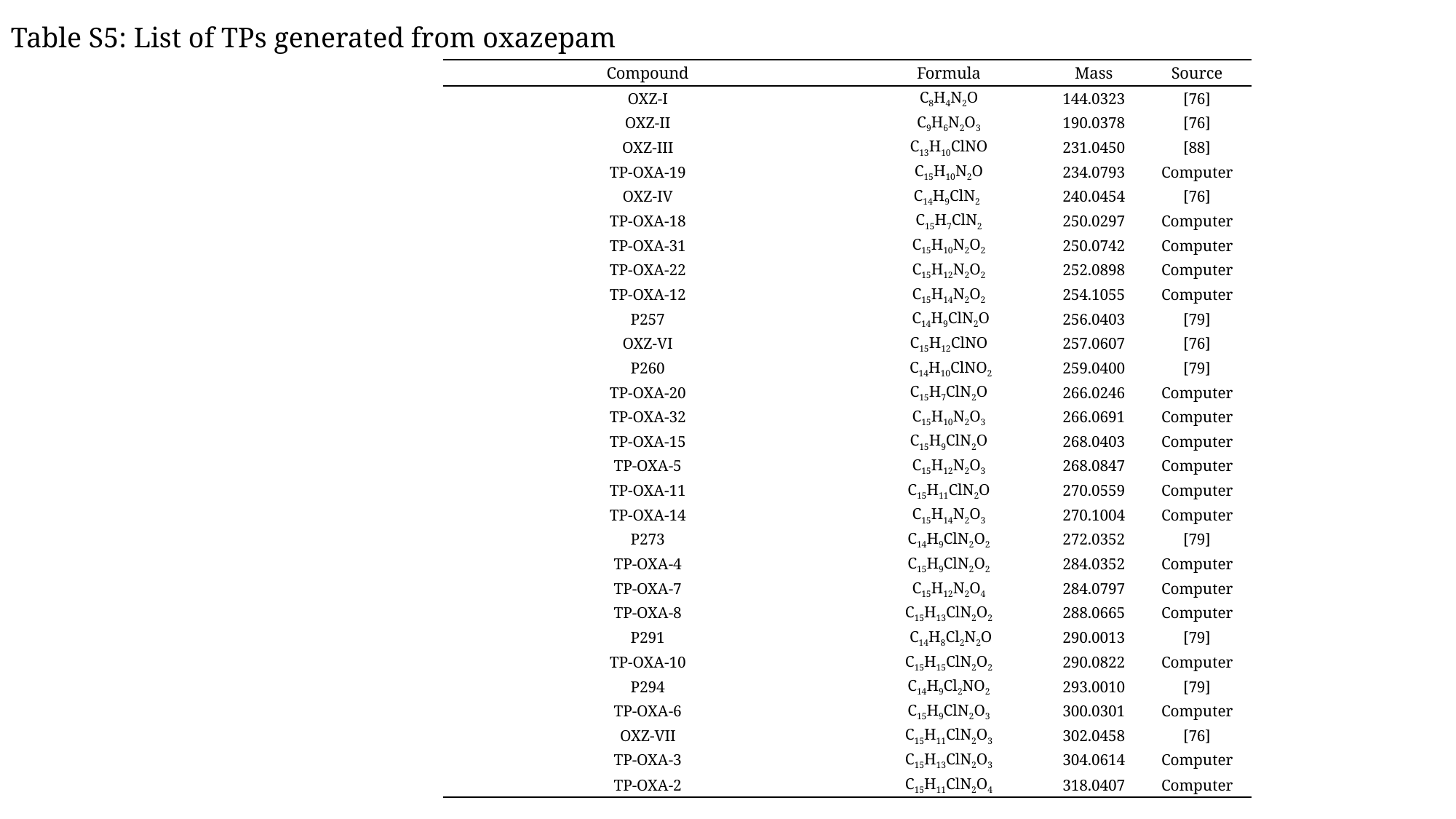

Table S5: List of TPs generated from oxazepam
| Compound | Formula | Mass | Source |
| --- | --- | --- | --- |
| OXZ-I | C8H4N2O | 144.0323 | [76] |
| OXZ-II | C9H6N2O3 | 190.0378 | [76] |
| OXZ-III | C13H10ClNO | 231.0450 | [88] |
| TP-OXA-19 | C15H10N2O | 234.0793 | Computer |
| OXZ-IV | C14H9ClN2 | 240.0454 | [76] |
| TP-OXA-18 | C15H7ClN2 | 250.0297 | Computer |
| TP-OXA-31 | C15H10N2O2 | 250.0742 | Computer |
| TP-OXA-22 | C15H12N2O2 | 252.0898 | Computer |
| TP-OXA-12 | C15H14N2O2 | 254.1055 | Computer |
| P257 | C14H9ClN2O | 256.0403 | [79] |
| OXZ-VI | C15H12ClNO | 257.0607 | [76] |
| P260 | C14H10ClNO2 | 259.0400 | [79] |
| TP-OXA-20 | C15H7ClN2O | 266.0246 | Computer |
| TP-OXA-32 | C15H10N2O3 | 266.0691 | Computer |
| TP-OXA-15 | C15H9ClN2O | 268.0403 | Computer |
| TP-OXA-5 | C15H12N2O3 | 268.0847 | Computer |
| TP-OXA-11 | C15H11ClN2O | 270.0559 | Computer |
| TP-OXA-14 | C15H14N2O3 | 270.1004 | Computer |
| P273 | C14H9ClN2O2 | 272.0352 | [79] |
| TP-OXA-4 | C15H9ClN2O2 | 284.0352 | Computer |
| TP-OXA-7 | C15H12N2O4 | 284.0797 | Computer |
| TP-OXA-8 | C15H13ClN2O2 | 288.0665 | Computer |
| P291 | C14H8Cl2N2O | 290.0013 | [79] |
| TP-OXA-10 | C15H15ClN2O2 | 290.0822 | Computer |
| P294 | C14H9Cl2NO2 | 293.0010 | [79] |
| TP-OXA-6 | C15H9ClN2O3 | 300.0301 | Computer |
| OXZ-VII | C15H11ClN2O3 | 302.0458 | [76] |
| TP-OXA-3 | C15H13ClN2O3 | 304.0614 | Computer |
| TP-OXA-2 | C15H11ClN2O4 | 318.0407 | Computer |

## Slide 33
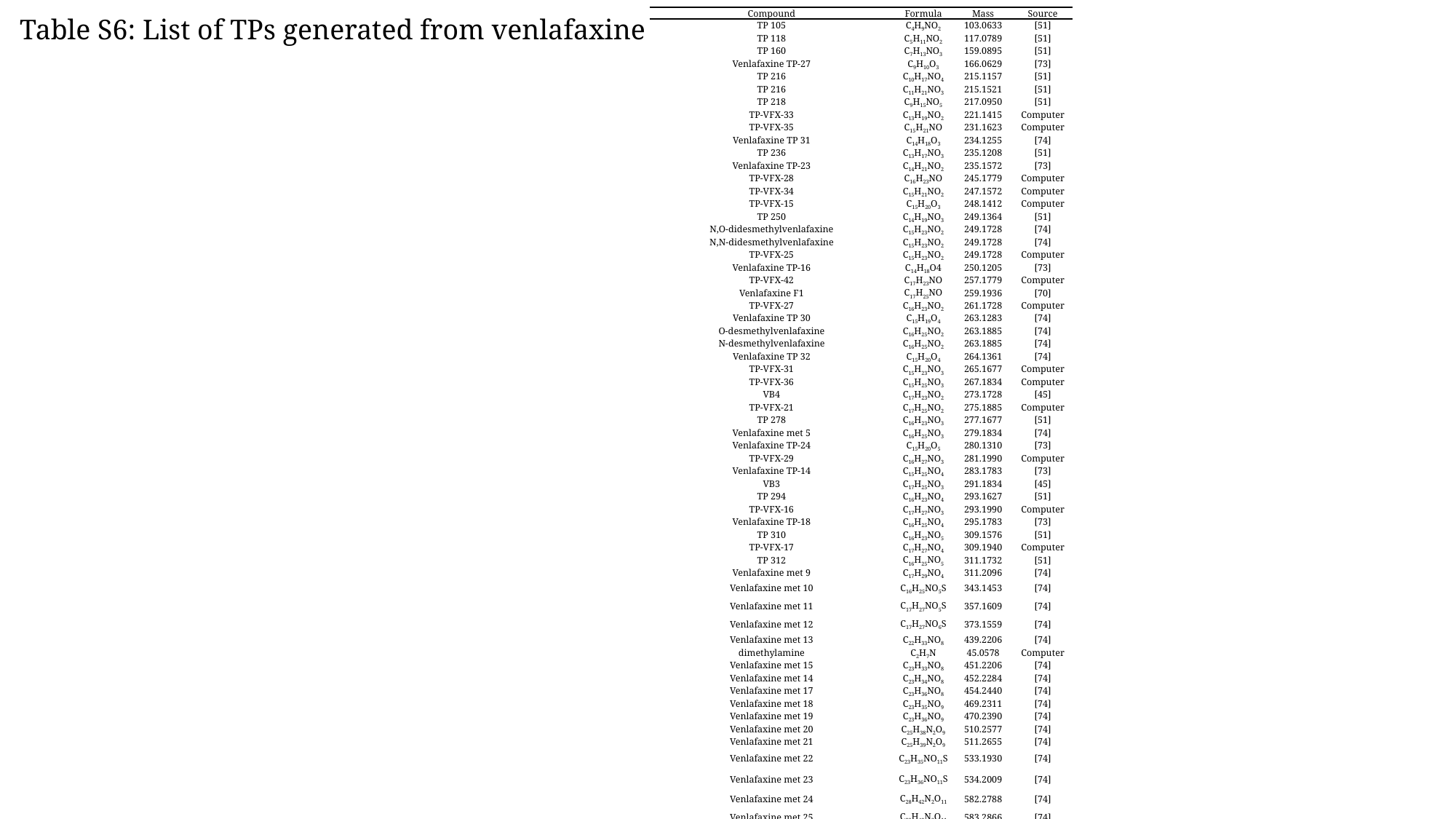

Table S6: List of TPs generated from venlafaxine
| Compound | Formula | Mass | Source |
| --- | --- | --- | --- |
| TP 105 | C4H9NO2 | 103.0633 | [51] |
| TP 118 | C5H11NO2 | 117.0789 | [51] |
| TP 160 | C7H13NO3 | 159.0895 | [51] |
| Venlafaxine TP-27 | C9H10O3 | 166.0629 | [73] |
| TP 216 | C10H17NO4 | 215.1157 | [51] |
| TP 216 | C11H21NO3 | 215.1521 | [51] |
| TP 218 | C9H15NO5 | 217.0950 | [51] |
| TP-VFX-33 | C13H19NO2 | 221.1415 | Computer |
| TP-VFX-35 | C15H21NO | 231.1623 | Computer |
| Venlafaxine TP 31 | C14H18O3 | 234.1255 | [74] |
| TP 236 | C13H17NO3 | 235.1208 | [51] |
| Venlafaxine TP-23 | C14H21NO2 | 235.1572 | [73] |
| TP-VFX-28 | C16H23NO | 245.1779 | Computer |
| TP-VFX-34 | C15H21NO2 | 247.1572 | Computer |
| TP-VFX-15 | C15H20O3 | 248.1412 | Computer |
| TP 250 | C14H19NO3 | 249.1364 | [51] |
| N,O-didesmethylvenlafaxine | C15H23NO2 | 249.1728 | [74] |
| N,N-didesmethylvenlafaxine | C15H23NO2 | 249.1728 | [74] |
| TP-VFX-25 | C15H23NO2 | 249.1728 | Computer |
| Venlafaxine TP-16 | C14H18O4 | 250.1205 | [73] |
| TP-VFX-42 | C17H23NO | 257.1779 | Computer |
| Venlafaxine F1 | C17H25NO | 259.1936 | [70] |
| TP-VFX-27 | C16H23NO2 | 261.1728 | Computer |
| Venlafaxine TP 30 | C15H19O4 | 263.1283 | [74] |
| O-desmethylvenlafaxine | C16H25NO2 | 263.1885 | [74] |
| N-desmethylvenlafaxine | C16H25NO2 | 263.1885 | [74] |
| Venlafaxine TP 32 | C15H20O4 | 264.1361 | [74] |
| TP-VFX-31 | C15H23NO3 | 265.1677 | Computer |
| TP-VFX-36 | C15H25NO3 | 267.1834 | Computer |
| VB4 | C17H23NO2 | 273.1728 | [45] |
| TP-VFX-21 | C17H25NO2 | 275.1885 | Computer |
| TP 278 | C16H23NO3 | 277.1677 | [51] |
| Venlafaxine met 5 | C16H25NO3 | 279.1834 | [74] |
| Venlafaxine TP-24 | C15H20O5 | 280.1310 | [73] |
| TP-VFX-29 | C16H27NO3 | 281.1990 | Computer |
| Venlafaxine TP-14 | C15H25NO4 | 283.1783 | [73] |
| VB3 | C17H25NO3 | 291.1834 | [45] |
| TP 294 | C16H23NO4 | 293.1627 | [51] |
| TP-VFX-16 | C17H27NO3 | 293.1990 | Computer |
| Venlafaxine TP-18 | C16H25NO4 | 295.1783 | [73] |
| TP 310 | C16H23NO5 | 309.1576 | [51] |
| TP-VFX-17 | C17H27NO4 | 309.1940 | Computer |
| TP 312 | C16H25NO5 | 311.1732 | [51] |
| Venlafaxine met 9 | C17H29NO4 | 311.2096 | [74] |
| Venlafaxine met 10 | C16H25NO5S | 343.1453 | [74] |
| Venlafaxine met 11 | C17H27NO5S | 357.1609 | [74] |
| Venlafaxine met 12 | C17H27NO6S | 373.1559 | [74] |
| Venlafaxine met 13 | C22H33NO8 | 439.2206 | [74] |
| dimethylamine | C2H7N | 45.0578 | Computer |
| Venlafaxine met 15 | C23H33NO8 | 451.2206 | [74] |
| Venlafaxine met 14 | C23H34NO8 | 452.2284 | [74] |
| Venlafaxine met 17 | C23H36NO8 | 454.2440 | [74] |
| Venlafaxine met 18 | C23H35NO9 | 469.2311 | [74] |
| Venlafaxine met 19 | C23H36NO9 | 470.2390 | [74] |
| Venlafaxine met 20 | C25H38N2O9 | 510.2577 | [74] |
| Venlafaxine met 21 | C25H39N2O9 | 511.2655 | [74] |
| Venlafaxine met 22 | C23H35NO11S | 533.1930 | [74] |
| Venlafaxine met 23 | C23H36NO11S | 534.2009 | [74] |
| Venlafaxine met 24 | C28H42N2O11 | 582.2788 | [74] |
| Venlafaxine met 25 | C28H43N2O11 | 583.2866 | [74] |
| Venlafaxine met 26 | C27H44N4O9S | 600.2829 | [74] |
| Venlafaxine met 27 | C29H43NO14 | 629.2683 | [74] |
| Venlafaxine met 28 | C29H44NO14 | 630.2761 | [74] |
| Venlafaxine met 29 | C29H46NO14 | 632.2918 | [74] |
| Venlafaxine TP-26 | C6H10O | 98.0731 | [73] |

## Slide 34
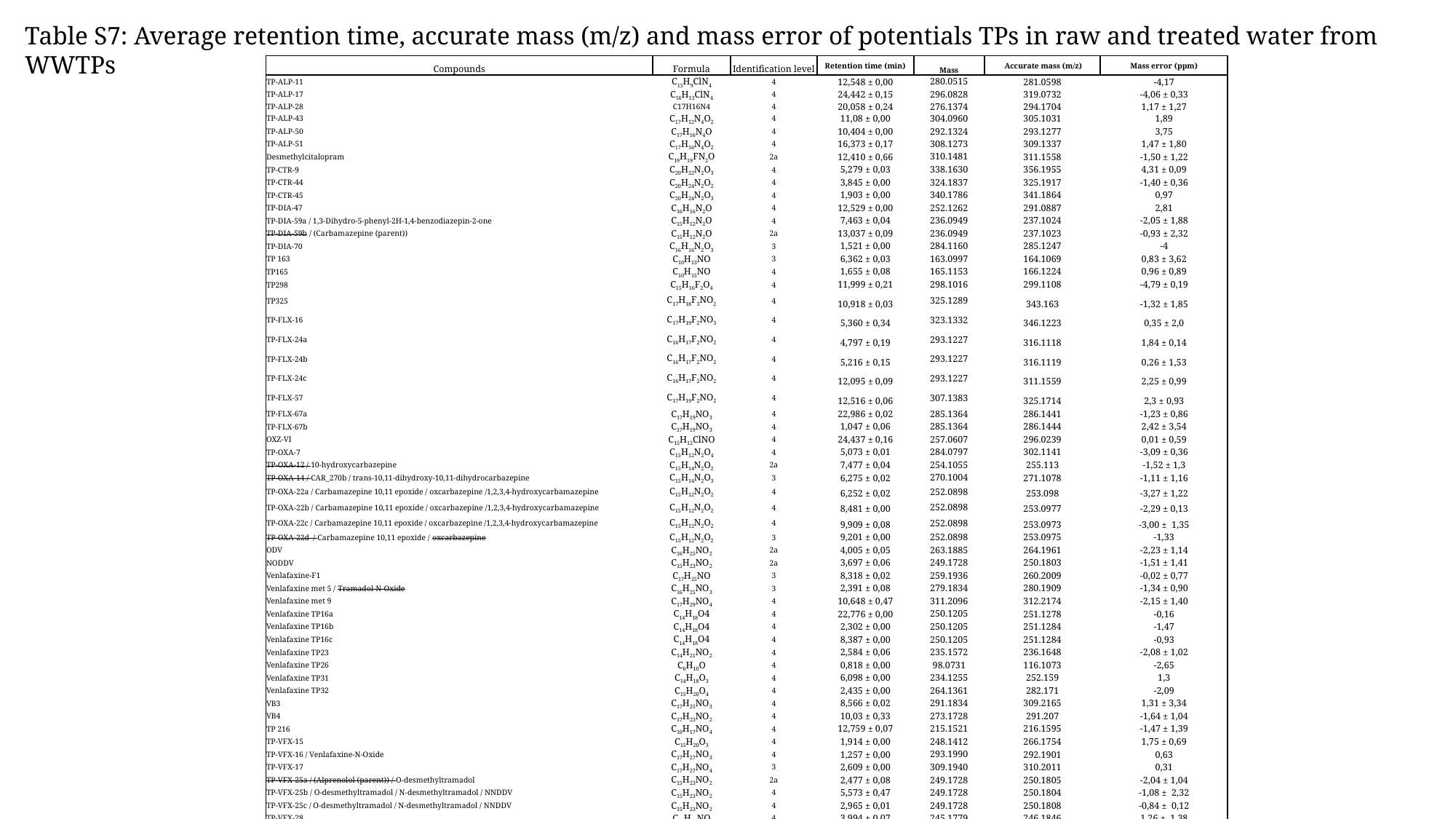

Table S7: Average retention time, accurate mass (m/z) and mass error of potentials TPs in raw and treated water from WWTPs
| Compounds | Formula | Identification level | Retention time (min) | Mass | Accurate mass (m/z) | Mass error (ppm) |
| --- | --- | --- | --- | --- | --- | --- |
| TP-ALP-11 | C15H9ClN4 | 4 | 12,548 ± 0,00 | 280.0515 | 281.0598 | -4,17 |
| TP-ALP-17 | C16H13ClN4 | 4 | 24,442 ± 0,15 | 296.0828 | 319.0732 | -4,06 ± 0,33 |
| TP-ALP-28 | C17H16N4 | 4 | 20,058 ± 0,24 | 276.1374 | 294.1704 | 1,17 ± 1,27 |
| TP-ALP-43 | C17H12N4O2 | 4 | 11,08 ± 0,00 | 304.0960 | 305.1031 | 1,89 |
| TP-ALP-50 | C17H16N4O | 4 | 10,404 ± 0,00 | 292.1324 | 293.1277 | 3,75 |
| TP-ALP-51 | C17H16N4O2 | 4 | 16,373 ± 0,17 | 308.1273 | 309.1337 | 1,47 ± 1,80 |
| Desmethylcitalopram | C19H19FN2O | 2a | 12,410 ± 0,66 | 310.1481 | 311.1558 | -1,50 ± 1,22 |
| TP-CTR-9 | C20H22N2O3 | 4 | 5,279 ± 0,03 | 338.1630 | 356.1955 | 4,31 ± 0,09 |
| TP-CTR-44 | C20H24N2O2 | 4 | 3,845 ± 0,00 | 324.1837 | 325.1917 | -1,40 ± 0,36 |
| TP-CTR-45 | C20H24N2O3 | 4 | 1,903 ± 0,00 | 340.1786 | 341.1864 | 0,97 |
| TP-DIA-47 | C16H16N2O | 4 | 12,529 ± 0,00 | 252.1262 | 291.0887 | 2,81 |
| TP-DIA-59a / 1,3-Dihydro-5-phenyl-2H-1,4-benzodiazepin-2-one | C15H12N2O | 4 | 7,463 ± 0,04 | 236.0949 | 237.1024 | -2,05 ± 1,88 |
| TP-DIA-59b / (Carbamazepine (parent)) | C15H12N2O | 2a | 13,037 ± 0,09 | 236.0949 | 237.1023 | -0,93 ± 2,32 |
| TP-DIA-70 | C16H16N2O3 | 3 | 1,521 ± 0,00 | 284.1160 | 285.1247 | -4 |
| TP 163 | C10H13NO | 3 | 6,362 ± 0,03 | 163.0997 | 164.1069 | 0,83 ± 3,62 |
| TP165 | C10H15NO | 4 | 1,655 ± 0,08 | 165.1153 | 166.1224 | 0,96 ± 0,89 |
| TP298 | C15H16F2O4 | 4 | 11,999 ± 0,21 | 298.1016 | 299.1108 | -4,79 ± 0,19 |
| TP325 | C17H18F3NO2 | 4 | 10,918 ± 0,03 | 325.1289 | 343.163 | -1,32 ± 1,85 |
| TP-FLX-16 | C17H19F2NO3 | 4 | 5,360 ± 0,34 | 323.1332 | 346.1223 | 0,35 ± 2,0 |
| TP-FLX-24a | C16H17F2NO2 | 4 | 4,797 ± 0,19 | 293.1227 | 316.1118 | 1,84 ± 0,14 |
| TP-FLX-24b | C16H17F2NO2 | 4 | 5,216 ± 0,15 | 293.1227 | 316.1119 | 0,26 ± 1,53 |
| TP-FLX-24c | C16H17F2NO2 | 4 | 12,095 ± 0,09 | 293.1227 | 311.1559 | 2,25 ± 0,99 |
| TP-FLX-57 | C17H19F2NO2 | 4 | 12,516 ± 0,06 | 307.1383 | 325.1714 | 2,3 ± 0,93 |
| TP-FLX-67a | C17H19NO3 | 4 | 22,986 ± 0,02 | 285.1364 | 286.1441 | -1,23 ± 0,86 |
| TP-FLX-67b | C17H19NO3 | 4 | 1,047 ± 0,06 | 285.1364 | 286.1444 | 2,42 ± 3,54 |
| OXZ-VI | C15H12ClNO | 4 | 24,437 ± 0,16 | 257.0607 | 296.0239 | 0,01 ± 0,59 |
| TP-OXA-7 | C15H12N2O4 | 4 | 5,073 ± 0,01 | 284.0797 | 302.1141 | -3,09 ± 0,36 |
| TP-OXA-12 / 10-hydroxycarbazepine | C15H14N2O2 | 2a | 7,477 ± 0,04 | 254.1055 | 255.113 | -1,52 ± 1,3 |
| TP-OXA-14 / CAR\_270b / trans-10,11-dihydroxy-10,11-dihydrocarbazepine | C15H14N2O3 | 3 | 6,275 ± 0,02 | 270.1004 | 271.1078 | -1,11 ± 1,16 |
| TP-OXA-22a / Carbamazepine 10,11 epoxide / oxcarbazepine /1,2,3,4-hydroxycarbamazepine | C15H12N2O2 | 4 | 6,252 ± 0,02 | 252.0898 | 253.098 | -3,27 ± 1,22 |
| TP-OXA-22b / Carbamazepine 10,11 epoxide / oxcarbazepine /1,2,3,4-hydroxycarbamazepine | C15H12N2O2 | 4 | 8,481 ± 0,00 | 252.0898 | 253.0977 | -2,29 ± 0,13 |
| TP-OXA-22c / Carbamazepine 10,11 epoxide / oxcarbazepine /1,2,3,4-hydroxycarbamazepine | C15H12N2O2 | 4 | 9,909 ± 0,08 | 252.0898 | 253.0973 | -3,00 ± 1,35 |
| TP-OXA-22d / Carbamazepine 10,11 epoxide / oxcarbazepine | C15H12N2O2 | 3 | 9,201 ± 0,00 | 252.0898 | 253.0975 | -1,33 |
| ODV | C16H25NO2 | 2a | 4,005 ± 0,05 | 263.1885 | 264.1961 | -2,23 ± 1,14 |
| NODDV | C15H23NO2 | 2a | 3,697 ± 0,06 | 249.1728 | 250.1803 | -1,51 ± 1,41 |
| Venlafaxine-F1 | C17H25NO | 3 | 8,318 ± 0,02 | 259.1936 | 260.2009 | -0,02 ± 0,77 |
| Venlafaxine met 5 / Tramadol-N-Oxide | C16H25NO3 | 3 | 2,391 ± 0,08 | 279.1834 | 280.1909 | -1,34 ± 0,90 |
| Venlafaxine met 9 | C17H29NO4 | 4 | 10,648 ± 0,47 | 311.2096 | 312.2174 | -2,15 ± 1,40 |
| Venlafaxine TP16a | C14H18O4 | 4 | 22,776 ± 0,00 | 250.1205 | 251.1278 | -0,16 |
| Venlafaxine TP16b | C14H18O4 | 4 | 2,302 ± 0,00 | 250.1205 | 251.1284 | -1,47 |
| Venlafaxine TP16c | C14H18O4 | 4 | 8,387 ± 0,00 | 250.1205 | 251.1284 | -0,93 |
| Venlafaxine TP23 | C14H21NO2 | 4 | 2,584 ± 0,06 | 235.1572 | 236.1648 | -2,08 ± 1,02 |
| Venlafaxine TP26 | C6H10O | 4 | 0,818 ± 0,00 | 98.0731 | 116.1073 | -2,65 |
| Venlafaxine TP31 | C14H18O3 | 4 | 6,098 ± 0,00 | 234.1255 | 252.159 | 1,3 |
| Venlafaxine TP32 | C15H20O4 | 4 | 2,435 ± 0,00 | 264.1361 | 282.171 | -2,09 |
| VB3 | C17H25NO3 | 4 | 8,566 ± 0,02 | 291.1834 | 309.2165 | 1,31 ± 3,34 |
| VB4 | C17H23NO2 | 4 | 10,03 ± 0,33 | 273.1728 | 291.207 | -1,64 ± 1,04 |
| TP 216 | C10H17NO4 | 4 | 12,759 ± 0,07 | 215.1521 | 216.1595 | -1,47 ± 1,39 |
| TP-VFX-15 | C15H20O3 | 4 | 1,914 ± 0,00 | 248.1412 | 266.1754 | 1,75 ± 0,69 |
| TP-VFX-16 / Venlafaxine-N-Oxide | C17H27NO3 | 4 | 1,257 ± 0,00 | 293.1990 | 292.1901 | 0,63 |
| TP-VFX-17 | C17H27NO4 | 3 | 2,609 ± 0,00 | 309.1940 | 310.2011 | 0,31 |
| TP-VFX-25a / (Alprenolol (parent)) / O-desmethyltramadol | C15H23NO2 | 2a | 2,477 ± 0,08 | 249.1728 | 250.1805 | -2,04 ± 1,04 |
| TP-VFX-25b / O-desmethyltramadol / N-desmethyltramadol / NNDDV | C15H23NO2 | 4 | 5,573 ± 0,47 | 249.1728 | 250.1804 | -1,08 ± 2,32 |
| TP-VFX-25c / O-desmethyltramadol / N-desmethyltramadol / NNDDV | C15H23NO2 | 4 | 2,965 ± 0,01 | 249.1728 | 250.1808 | -0,84 ± 0,12 |
| TP-VFX-28 | C16H23NO | 4 | 3,994 ± 0,07 | 245.1779 | 246.1846 | 1,26 ± 1,38 |
| TP-VFX-29 | C16H27NO3 | 4 | 18,849 ± 0,00 | 281.1990 | 282.2052 | 4,26 ± 0,08 |
| TP-VFX-34 | C15H21NO2 | 4 | 2,042 ± 0,11 | 247.1572 | 248.1642 | 1,12 ± 0,87 |
| TP-VFX-35 | C15H21NO | 4 | 1,166 ± 0,11 | 231.1623 | 249.1962 | -0,55 ± 1,55 |
| TP-VFX-36 | C15H25NO3 | 4 | 5,306 ± 0,07 | 267.1834 | 268.1908 | -0,54 ± 0,19 |
| TP-VFX-42 | C17H23NO | 4 | 4,868 ± 0,06 | 257.1779 | 258.1851 | 0,08 ± 1,92 |

## Slide 35
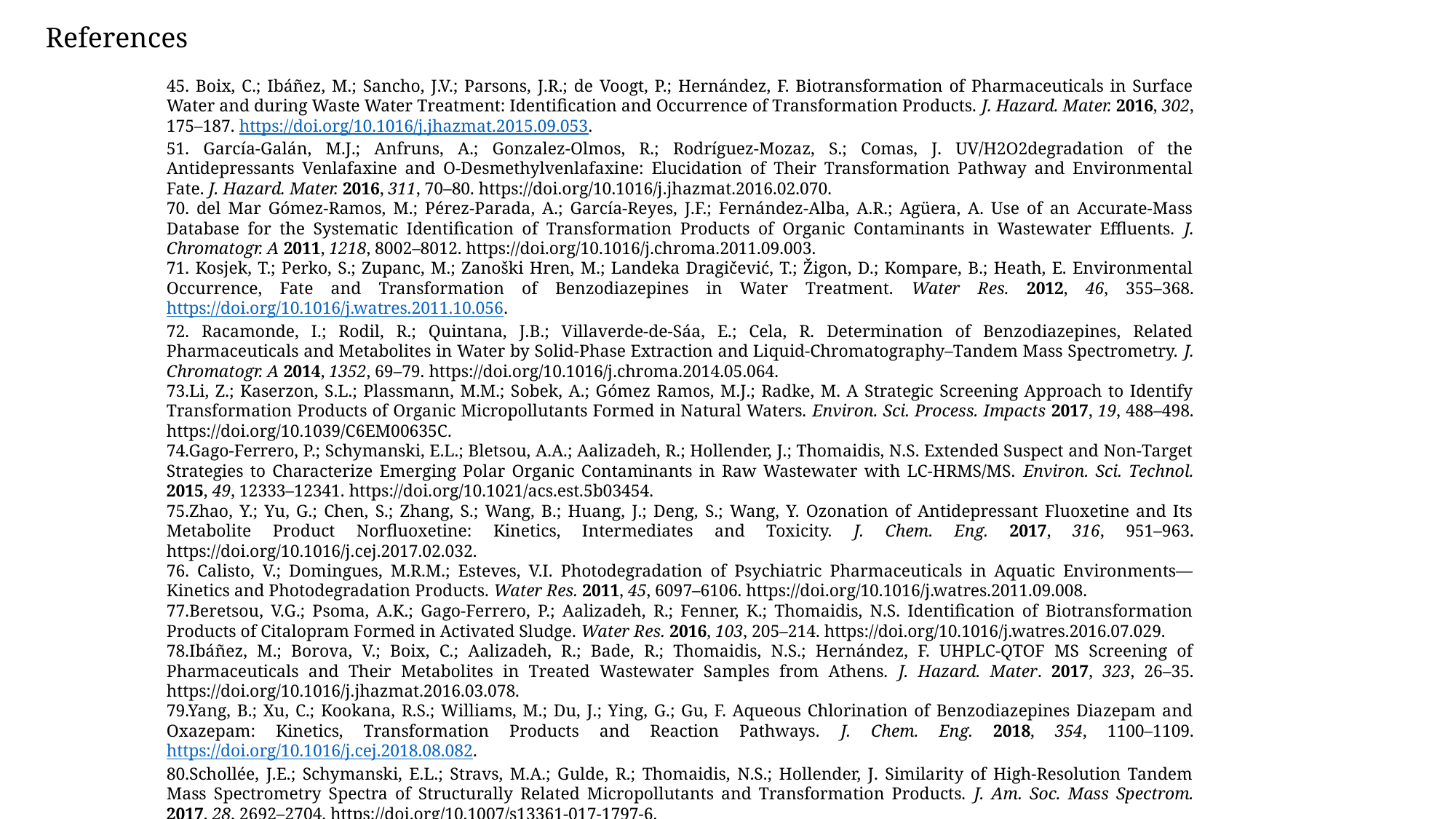

References
45. Boix, C.; Ibáñez, M.; Sancho, J.V.; Parsons, J.R.; de Voogt, P.; Hernández, F. Biotransformation of Pharmaceuticals in Surface Water and during Waste Water Treatment: Identification and Occurrence of Transformation Products. J. Hazard. Mater. 2016, 302, 175–187. https://doi.org/10.1016/j.jhazmat.2015.09.053.
51. García-Galán, M.J.; Anfruns, A.; Gonzalez-Olmos, R.; Rodríguez-Mozaz, S.; Comas, J. UV/H2O2degradation of the Antidepressants Venlafaxine and O-Desmethylvenlafaxine: Elucidation of Their Transformation Pathway and Environmental Fate. J. Hazard. Mater. 2016, 311, 70–80. https://doi.org/10.1016/j.jhazmat.2016.02.070.
70. del Mar Gómez-Ramos, M.; Pérez-Parada, A.; García-Reyes, J.F.; Fernández-Alba, A.R.; Agüera, A. Use of an Accurate-Mass Database for the Systematic Identification of Transformation Products of Organic Contaminants in Wastewater Effluents. J. Chromatogr. A 2011, 1218, 8002–8012. https://doi.org/10.1016/j.chroma.2011.09.003.
71. Kosjek, T.; Perko, S.; Zupanc, M.; Zanoški Hren, M.; Landeka Dragičević, T.; Žigon, D.; Kompare, B.; Heath, E. Environmental Occurrence, Fate and Transformation of Benzodiazepines in Water Treatment. Water Res. 2012, 46, 355–368. https://doi.org/10.1016/j.watres.2011.10.056.
72. Racamonde, I.; Rodil, R.; Quintana, J.B.; Villaverde-de-Sáa, E.; Cela, R. Determination of Benzodiazepines, Related Pharmaceuticals and Metabolites in Water by Solid-Phase Extraction and Liquid-Chromatography–Tandem Mass Spectrometry. J. Chromatogr. A 2014, 1352, 69–79. https://doi.org/10.1016/j.chroma.2014.05.064.
73.Li, Z.; Kaserzon, S.L.; Plassmann, M.M.; Sobek, A.; Gómez Ramos, M.J.; Radke, M. A Strategic Screening Approach to Identify Transformation Products of Organic Micropollutants Formed in Natural Waters. Environ. Sci. Process. Impacts 2017, 19, 488–498. https://doi.org/10.1039/C6EM00635C.
74.Gago-Ferrero, P.; Schymanski, E.L.; Bletsou, A.A.; Aalizadeh, R.; Hollender, J.; Thomaidis, N.S. Extended Suspect and Non-Target Strategies to Characterize Emerging Polar Organic Contaminants in Raw Wastewater with LC-HRMS/MS. Environ. Sci. Technol. 2015, 49, 12333–12341. https://doi.org/10.1021/acs.est.5b03454.
75.Zhao, Y.; Yu, G.; Chen, S.; Zhang, S.; Wang, B.; Huang, J.; Deng, S.; Wang, Y. Ozonation of Antidepressant Fluoxetine and Its Metabolite Product Norfluoxetine: Kinetics, Intermediates and Toxicity. J. Chem. Eng. 2017, 316, 951–963. https://doi.org/10.1016/j.cej.2017.02.032.
76. Calisto, V.; Domingues, M.R.M.; Esteves, V.I. Photodegradation of Psychiatric Pharmaceuticals in Aquatic Environments—Kinetics and Photodegradation Products. Water Res. 2011, 45, 6097–6106. https://doi.org/10.1016/j.watres.2011.09.008.
77.Beretsou, V.G.; Psoma, A.K.; Gago-Ferrero, P.; Aalizadeh, R.; Fenner, K.; Thomaidis, N.S. Identification of Biotransformation Products of Citalopram Formed in Activated Sludge. Water Res. 2016, 103, 205–214. https://doi.org/10.1016/j.watres.2016.07.029.
78.Ibáñez, M.; Borova, V.; Boix, C.; Aalizadeh, R.; Bade, R.; Thomaidis, N.S.; Hernández, F. UHPLC-QTOF MS Screening of Pharmaceuticals and Their Metabolites in Treated Wastewater Samples from Athens. J. Hazard. Mater. 2017, 323, 26–35. https://doi.org/10.1016/j.jhazmat.2016.03.078.
79.Yang, B.; Xu, C.; Kookana, R.S.; Williams, M.; Du, J.; Ying, G.; Gu, F. Aqueous Chlorination of Benzodiazepines Diazepam and Oxazepam: Kinetics, Transformation Products and Reaction Pathways. J. Chem. Eng. 2018, 354, 1100–1109. https://doi.org/10.1016/j.cej.2018.08.082.
80.Schollée, J.E.; Schymanski, E.L.; Stravs, M.A.; Gulde, R.; Thomaidis, N.S.; Hollender, J. Similarity of High-Resolution Tandem Mass Spectrometry Spectra of Structurally Related Micropollutants and Transformation Products. J. Am. Soc. Mass Spectrom. 2017, 28, 2692–2704. https://doi.org/10.1007/s13361-017-1797-6.
